# Enantiomerically Pure Quinoline‐Based κ‐Opioid Receptor Agonists: Chemoenzymatic Synthesis and Pharmacological Evaluation

**DOI:** 10.1002/cmdc.202000300

**Published:** 2020-07-02

**Authors:** Benedikt Martin, Dirk Schepmann, Freddy A. Bernal, Thomas J. Schmidt, Tao Che, Karin Loser, Bernhard Wünsch

**Affiliations:** ^1^ Institut für Pharmazeutische und Medizinische Chemie der Universität Münster Corrensstraße 48 48149 Münster Germany; ^2^ Institut für Pharmazeutische Biologie und Phytochemie der Universität Münster Corrensstraße 48 48149 Münster Germany; ^3^ Department of Anesthesiology Washington University School of Medicine 660 S. Euclid Ave. St. Louis MO 63110 USA; ^4^ Department of Dermatology University of Münster von-Esmarch-Street 58 48149 Münster Germany; ^5^ Cells-in-Motion Cluster of Excellence (EXC 1003-CiM) Westfälische Wilhelms-Universität Münster 48149 Münster Germany

**Keywords:** anti-inflammatory activity, diastereoselective synthesis, KOR agonists, lipase-catalyzed kinetic resolution, perhydroquinolines

## Abstract

Racemic _K_‐opioid receptor (KOR) agonist 2‐(3,4‐dichlorophenyl)‐1‐[(4a*RS*,8*SR*,8a*SR*)‐8‐(pyrrolidin‐1‐yl)‐3,4,4a,5,6,7,8,8a‐octahydroquinolin‐1(2*H*)‐yl]ethan‐1‐one ((±)‐**4**) was prepared in a diastereoselective synthesis. The first key step of the synthesis was the diastereoselective hydrogenation of the silyl ether of 1,2,3,4‐tetrahydroquinoin‐8‐ol ((±)‐**9**) to afford *cis*,*cis*‐configured perhydroquinoline derivative (±)‐**10**. Removal of the TBDMS protecting group led to a β‐aminoalcohol that reacted with SO_2_Cl_2_ to form an oxathiazolidine. Nucleophilic substitution with pyrrolidine resulted in the desired *cis*,*trans*‐configured perhydroquinoline upon inversion of the configuration. In order to obtain enantiomerically pure KOR agonists **4** (99.8 % *ee*) and *ent*‐**4** (99.0 % *ee*), 1,2,3,4‐tetrahydroquinolin‐8‐ols (*R*)‐**8** (99.1 % *ee*) and (*S*)‐**8** (98.4 % *ee*) were resolved by an enantioselective acetylation catalyzed by Amano lipase PS‐IM. The absolute configuration was determined by CD spectroscopy. The 4a*R*,8*S*,8a*S*‐configured enantiomer **4** showed sub‐nanomolar KOR affinity (*K*_i_=0.81 nM), which is more than 200 times higher than the KOR affinity of its enantiomer *ent*‐**4**. In the cAMP assay and the Tango β‐arrestin‐2 recruitment assay, **4** behaved as a KOR agonist. Upon incubation of human macrophages, human dendritic cells, and mouse myeloid immune cells with **4**, the number of cells expressing co‐stimulatory receptor CD86 and proinflammatory cytokines interleukin 6 and tumor necrosis factor α was significantly reduced; this indicates the strong anti‐inflammatory activity of **4**. The anti‐inflammatory effects correlated well with the KOR affinity: (4a*R*,8*S*,8a*S*)‐**4** was slightly more potent than the racemic mixture (±)‐**4**, and the distomer *ent*‐**4** was almost inactive.

## Graphical abstract

***cis,trans*** **‐Configured** perhydroquinoline‐based enantiomerically pure κ‐opioid receptor (KOR) agonists were prepared in a stereoselective synthesis. The 4a*R*,8*S*,8a*S*‐configured enantiomer (see graphic) showed sub‐nanomolar KOR affinity – more than 200 times higher than that of its enantiomer. In two different functional assays, it behaved as KOR agonist. The strong anti‐inflammatory effect was shown upon incubation of various human immune cells.

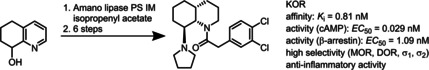

## 1 Introduction

The group of opioid receptors consists of four subtypes. They are termed according to their prototypical agonists μ‐opioid receptor (MOR, morphine), κ‐opioid receptor (KOR, ketocyclazocine) and opioid receptor‐like 1 (ORL1) receptor (NOR, nociceptine) or according to the tissue, in which the receptor was detected first, δ‐opioid receptor (DOR, vas deferens).^1^ Initially, the σ receptor was considered as an additional opioid receptor subtype, but this classification was later corrected and the σ receptor was reclassified as non‐opioid receptor type.^1^ All opioid receptors belong to the class A (rhodopsin‐like) γ subfamily of G protein‐coupled receptors (GPCRs).^2^ They share a common seven‐transmembrane architecture and are coupled predominantly to heterotrimeric G_i_/G_o_ proteins. Cloning of the four opioid receptors revealed a homology of more than 60 % with respect to their amino acid sequences.^3^

Activation of MOR, KOR and DOR leads to strong analgesia, but also to subtype specific side effects.^4^ Most of the clinically used analgesics are MOR agonists, such as morphine and methadone, which cause euphoria, constipation, respiratory depression, development of tolerance and addiction side effects.^5^ In contrast, KOR agonists do not elicit these typical MOR‐mediated side effects. Therefore, KOR represents an interesting target for the development of safer analgesics.^5^ However, KOR agonists are not devoid of side effects, since they induce centrally mediated dysphoria, sedation, and diuresis.^6^ A high density of KOR is not only found in the central nervous system, but also in peripheral cells (e. g., immune cells, including T cells and antigen presenting cells)^4^ and peripheral tissues (e. g., skin). KOR agonists are also of interest for the treatment of itching skin diseases, including pruritus, atopic dermatitis and psoriasis. In Japan, the morphinane derivative nalfurafine activating KOR in the sub‐nanomolar range (*K*_i_=0.17 nM)^7^ is approved for the treatment of uremic pruritus. Its antipruritic activity is linked to activation of peripheral KOR.^8^

**Figure 1:**
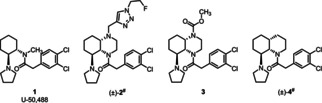
Important ethylenediamine‐based KOR agonists. ^#^ Only one enantiomer of racemic mixtures (±)‐**2** and (±)‐**4** is shown.

In a very recent study, the pharmacological effects of the prototypical KOR agonist U‐50,488 (**1**, *K*_i_=0.34 nM, Figure 1)^9^ in experimental autoimmune encephalomyelitis (EAE) were investigated. EAE is a commonly used animal model of multiple sclerosis (MS). KOR activation by U‐50,488 (**1**) alleviated the symptoms of EAE by preventing neuronal damage and promoting remyelination.^10^ The anti‐inflammatory properties of KOR agonists are mainly based on the downregulation of cell proliferation and the inhibition of immune cell activation, which leads to a reduced secretion of proinflammatory cytokines.^11^ Very recently, we have shown that the KOR agonist (±)‐**2** (*K*_i_=5.6 nM, EC_50_=2.8 nM, Figure 1), which we successfully synthesized according to a diastereoselective pathway, significantly decreased the expression of classical activation markers as well as interferon‐gamma (IFN‐γ), tumor‐necrosis‐factor‐alpha (TNF‐α), and interleukin 17 A (IL‐17 A). In parallel, the compound increased the release of anti‐inflammatory cytokines like IL‐10 in different mouse and human immune cell subsets pointing to an immunomodulatory effect of (±)‐**2**. In support of this, (±)‐**2** significantly reduced disease perpetuation in EAE. Experiments in KOR deficient mouse mutants confirmed that the observed beneficial effects of (±)‐**2** were indeed mediated by binding of the compound to KOR.^12^

The known KOR agonists belong to four structurally diverse classes of compounds: peptides derived from the endogenous KOR agonist dynorphin A,^13^ morphinoids,^14^ the non‐basic natural product salvinorin A,^15^ and ethylenediamines with U‐50,488 (**1**)^16^ being the first synthetic KOR agonist. Whereas one N‐atom of the ethylenediamine‐based KOR agonists should be connected with a (3,4‐dichlorophenyl)acetyl moiety, the second N‐atom should display basic properties. Usually, this N‐atom is part of a pyrrolidine ring, which after protonation forms a crucial ionic interaction with Asp138 in the KOR binding site.^17^ The (3,4‐dichlorophenyl)acetyl moiety at the other end of the ethylenediamine pharmacophore interacts with a hydrophobic pocket of KOR.

Recently, we have reported a series of conformationally constrained KOR agonists, in which the cyclohexane ring of U‐50,488 (**1**) is embedded in a perhydroquinoxaline scaffold.^18^, ^19^ A very high KOR affinity (*K*_i_=0.25 nM, EC_50_=2.0 nM) was found for the 4a*R*,5*S*,8a*S*‐configured perhydroquinoxaline **3** with a *cis*‐configured bicyclic ring system.^18^ (Figure 1) This result is in good accordance with the results of a patent describing racemic KOR agonists with a perhydroquinoline framework. In this series of compounds, the highest KOR affinity was found for racemic (±)‐**4** (IC_50_=2.7 nM) with a *cis*‐configured bicyclic framework and *trans*‐oriented pyrrolidinyl moiety (IC_50_=2.7 nM; Figure 1).^20^ As the *K*_i_ value of the KOR agonist (±)‐**4** was not reported in the patent, a direct comparison of the KOR affinity of enantiomerically pure **3** and racemic (±)‐**4** was not possible.

In the patent, the racemic KOR agonist (±)‐**4** was obtained by a non‐stereoselective synthesis and subsequent laborious and time‐consuming separation of racemic diastereomers. In this manuscript we would like to report an efficient diastereoselective synthesis of (±)‐**4**. A chemoenzymatic route should provide enantiomerically pure KOR agonists **4** and *ent*‐**4**, which will be thoroughly characterized in various biological assays.

## Results and Discussion

### Diastereoselective synthesis of racemic (±)‐4

**Scheme 1:**
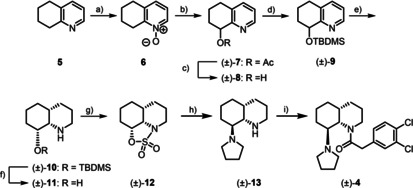
Synthesis of racemic KOR agonist (±)‐**4**. a) *m*CPBA, CH_2_Cl_2_, RT, 19 h, 81 %; b) (Ac)_2_O, 120 °C, 1 h, 88 %; c) aqu NaOH 1 M, CH_3_OH, RT, 1 h, 98 %; d) TBDMSCl, imidazole, DMF, RT, 16 h, 63 %; e) H_2_ (5 bar), Rh/Al_2_O_3_, AcOH, RT, 25 h, 94 %; f) TBAF, THF, RT, 18 h, 88 %; g) SO_2_Cl_2_, triethylamine, CH_2_Cl_2_, 0 °C→RT, 20 h, 34 %; h) pyrrolidine, CH_3_CN, 80 °C, 18 h, 82 %; i) 2‐(3,4‐dichlorophenyl)acetyl chloride, ethyldi(isopropyl)amine, CH_2_Cl_2_, RT, 1 h, 56 %. Only one enantiomer of the racemic mixtures is shown.

The diastereoselective synthesis of *cis,trans*‐configured racemic KOR agonist (±)‐**4** started with 5,6,7,8‐tetrahydroquinoline (**5**), which was oxidized with *m*‐chloroperoxybenzoic acid (*m*CPBA) to afford the N‐oxide **6** (Scheme 1). Boekelheide rearrangement^21^ of N‐oxide **6** was performed with Ac_2_O at 120 °C providing the acetate (±)‐**7**. After hydrolysis of acetate (±)‐**7** with NaOH, the alcohol (±)‐**8** was protected with a silyl group to give silyl ether (±)‐**9**. The bulky *tert*‐butyldimethylsilyl protective group was chosen, since it should direct the hydrogenation of the aromatic pyridine ring from the opposite side. Thus, hydrogenation (5 bar) of (±)‐**9** in the presence of catalyst Rh/Al_2_O_3_ in the solvent HOAc afforded diastereoselectively the *cis,cis*‐configured perhydroquinoline (±)‐**10** in 94 % yield.^22^ In addition to the control of the stereoselectivity, the silyl protective group should be stable during the hydrogenation conditions in pure HOAc used as solvent, which activates the pyridine ring and accelerates its hydrogenation^23^ providing increased yields of (±)‐**10**. The hydrogenation represents the key step in the nine‐step reaction sequence, since it establishes the configuration of all three centers of chirality with excellent diastereoselectivity.

**Figure 2:**
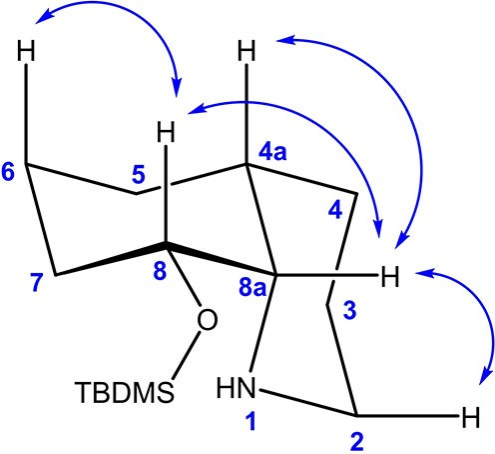
Neighborhood of protons of *cis,cis*‐configured perhydroquinoline (±)‐**10** determined by nuclear Overhauser effect (NOE).

The relative configuration of (±)‐**10** was determined by nuclear Overhauser effect. (Figure 2) Irradiation at the resonance frequency of 8a‐H (*δ*=2.85 ppm, t, *J*=3.5 Hz) led to increased signals of 2‐H_axial_ (*δ*=2.59 ppm), 4a‐H/7‐H_axial_ (*δ*=1.64 ppm) and 8‐H_axial_ (*δ*=3.27 ppm) in the recorded NOE difference spectrum. Additional interactions between 6‐H_axial_ (*δ*=1.31 ppm) and 8‐H (*δ*=3.27 ppm) were observed in a two‐dimensional NOESY spectrum. Both spectra are shown in the supporting information. These NOEs clearly prove the *cis*‐orientation of the protons 4a‐H, 8a‐H and 8‐H_axial_, which unequivocally confirms the *cis,cis*‐configuration of the perhydroquinoline ring of (±)‐**10**.

The TBDMS protective group of (±)‐**10** was removed with tetrabutylammonium fluoride (TBAF) to yield β‐aminoalcohol (±)‐**11**, which was treated with SO_2_Cl_2_ to give oxathiazolidine (±)‐**12** in 34 % yield. In the cyclic sulfuric ester amide (±)‐**12** the original OH moiety of the β‐aminoalcohol (±)‐**11** was activated for a S_N_2 reaction and the secondary amino moiety was protected. The nucleophilic substitution was carried out with pyrrolidine giving the diamine (±)‐**13** upon removal of the N‐protective group during work‐up under acidic conditions. This S_N_2 reaction of (±)‐**12** represents the second key step, as the inversion of the configuration at C‐8 during the nucleophilic attack led to the desired *cis*,*trans*‐configured perhydroquinoline system. Finally, the secondary amine (±)‐**13** was acylated with 2‐(3,4‐dichlorophenyl)acetyl chloride to afford the racemic KOR agonist (±)‐**4**.

### Chemoenzymatic synthesis of enantiomerically pure KOR agonists 4 and *ent*‐4

A lipase‐catalyzed kinetic resolution of racemic alcohol (±)‐**8** was planned as key step for the synthesis of the enantiomerically pure KOR agonists **4** and *ent*‐**4**. In a previous study, the lipase‐catalyzed kinetic resolution of alcohol (±)‐**8** had been carried out using *Candida antarctica* lipase as biocatalyst and vinyl acetate as acetylating reagent at 60 °C for 30 h.^24^ Under these conditions the acetate (*R*)‐**7** and the alcohol (*S*)‐**8** were obtained in 43 % and 44 % yield, respectively, with an enantiomeric purity >99 % *ee* in both cases.

**Figure 3:**
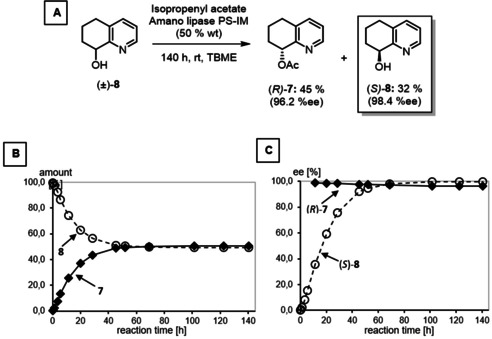
Lipase‐catalyzed kinetic resolution of racemic alcohol (±)‐**8**. A) Enantioselective acetylation of (±)‐**8** by using isopropenyl acetate in the presence of Amano lipase PS‐IM. B) Amounts [%] of remaining alcohol **8** and formed acetate **7** during the reaction period. C) Time course of the *ee* values of alcohol (*S*)‐**8** and acetate (*R*)‐**7** during the transformation.

Herein, Amano lipase PS‐IM was used for the kinetic resolution of racemic alcohol (±)‐**8**, which allowed milder reaction conditions, that is, reaction at room temperature, a longer reaction time, and isopropenyl acetate as acetylating agent. (Figure 3A) Although alcohol (*S*)‐**8** (97.9 % *ee*) and acetate (*R*)‐**7** (97.3 % *ee*) were present in the ratio 50 : 50 after a reaction time of 70 h (Figure 3B and C), excellent enantiomeric purity of remaining alcohol (*S*)‐**8** (98.4 % *ee*) was obtained after a very long reaction time of 140 h. (Figure 3C) As expected, the Amano lipase PS‐IM acetylated almost exclusively the *R*‐configured alcohol (*R*)‐**8**. Thus, a long reaction time led to conversion of even traces of alcohol (*R*)‐**8** into acetate (*R*)‐**7** providing excellent enantiomeric excess of the remaining alcohol (*S*)‐**8**. On the other hand, small amounts of alcohol (*S*)‐**8** were also acetylated by the lipase resulting in decreased enantiomeric purity of the acetate (*R*)‐**7**. After a reaction period of 140 h, acetate (*R*)‐**7** (96.2 % *ee*) and alcohol (*S*)‐**8** (98.4 % *ee*) were isolated in 45 and 32 % yield, respectively. Chiral HPLC of (*S*)‐**8** is shown in Figure S2 in the Supporting Information.

**Figure 4:**
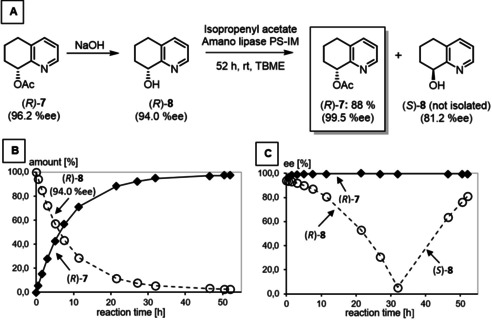
Saponification and second lipase‐catalyzed acetylation of enantiomerically enriched alcohol (*R*)‐**8** (94.0 % *ee*). A) Transformations of (*R*)‐**7** (96.2 % *ee*). B) Amounts [%] of remaining alcohol **8** and formed acetate **7** during the reaction period. C) Development of the *ee* values of alcohols (*R*)‐**8**/(*S*)‐**8** and acetate (*R*)‐**7** during the transformation. The decreased *ee* value of acetate (*R*)‐**7** at the beginning of the transformation is due to very small amounts of acetate **7** (0.04 %) in the starting material (*R*)‐**8**.

In order to increase the enantiomeric purity of *R*‐configured alcohol (*R*)‐**8**, acetate (*R*)‐**7** (96.2 % *ee*) was hydrolyzed with NaOH and the resulting alcohol (*R*)‐**8** (94.0 % *ee*) was used in a second lipase‐catalyzed acetylation reaction (Figure 4). As the lipase converted predominantly the *R*‐configured alcohol (*R*)‐**8** into its acetate (*R*)‐**7** (Figure 4A), the amount of (*R*)‐**8** decreased rapidly as the reaction proceeded (Figure 4B). However, the enantiomeric purity of the acetate (*R*)‐**7** was very high from the beginning. After a reaction period of 52 h, the transformation was stopped, and the acetate (*R*)‐**7** was obtained in 88 % yield and 99.5 % *ee* (Figure 4C). Finally, alcohol (*R*)‐**8** (99.1 % *ee*) was obtained by ester cleavage with NaOH (Figure S3).

Interestingly, the *ee* value of the *R*‐configured alcohol (*R*)‐**8** decreased continuously until it reached a minimum (<5 %*ee*) after 32 h. At this time point the amounts of desired (*R*)‐ and undesired *S*‐configured alcohols (*R*)‐**8** and (*S*)‐**8** are almost identical. After passing the minimum, *ee* values for *S*‐configured alcohol (*S*)‐**8** increased, as (*R*)‐**8** was transformed selectively.

**Figure 5:**
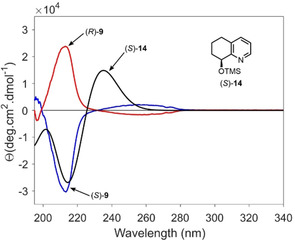
Recorded CD spectra of enantiomeric TBDMS ethers (*R*)‐**9** and (*S*)‐**9** and TDDFT calculated CD spectrum of *S*‐configured TMS‐ether (*S*)‐**14** as model compound.

In order to determine the absolute configuration of the enantiomeric alcohols (*R*)‐**8** and (*S*)‐**8**, CD spectra of the corresponding enantiomeric silyl ethers (*R*)‐**9** and (*S*)‐**9** were recorded in acetonitrile (Figure 5) Time‐dependent density functional theory (TDDFT) calculation at the B3LYP/6‐31G(d,p) level were used to correlate the observed Cotton effects with the corresponding absolute configuration. The trimethylsilyl ether (*S*)‐**14** was employed as model compound due its significantly higher conformational restrictions compared to (*R*)‐**9** and (*S*)‐**9** (but bearing the same chromophore), which translated in lower overall computational costs. As a result, the absolute configuration of both silyl ethers (*R*)‐**9** and (*S*)‐**9** was unequivocally assigned. (Figure 5) This configurational assignment perfectly agrees with the observation that Amano lipase PS‐IM preferably acetylates *R*‐configured secondary alcohols (in case the larger substituent has higher CIP priority than the smaller substituent).^24^

With both enantiomerically pure alcohols (*R*)‐**8** (99.1 % *ee*) and (*S*)‐**8** (98.4 % *ee*) in hand, the synthetic route already described (Scheme 1) was performed in order to obtain the KOR agonists **4** (4a*R*,8*S*,8a*S* configuration) and *ent*‐**4** (4a*S*,8*R*,8a*R* configuration). The corresponding reaction scheme is shown in Scheme S1. The final control of the enantiomeric purity showed some racemization of both enantiomers (**4**: 80.3 % *ee*, *ent*‐**4**: 66.3 % *ee*). We assume that partial racemization occurred during hydrogenation of the TBDMS ethers (*R*)‐**9** and (*S*)‐**9** under acidic conditions. Therefore, additional purification of the enantiomers **4** and *ent*‐**4** by chiral preparative HPLC using a Chiralpak® IA column was performed resulting in enantiomerically pure KOR agonists **4** (99.8 % *ee*) and *ent*‐**4** (99.0 % *ee*).

### KOR affinity, selectivity and agonistic activity

The KOR affinity of the synthesized quinoline derivatives and some reference compounds was investigated in competitive radioligand receptor binding assays using guinea pig brain homogenates as source of KOR and [^3^H]U‐69,593 as competitive radioligand.^25^, ^26^, ^27^ Table 1 summarizes the affinity data of perhydroquinolines, related perhydroquinoxalines and reference compounds.

**Table 1:** Affinities of racemic and enantiomerically pure perhydroquinolines and reference compounds for KOR and related receptors.

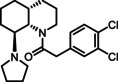						
Compd.	Configuration	*K*_i_±SEM [nM] ^[a,b]^				
		KOR	MOR	DOR	σ_1_	σ_2_
		[^3^H]U‐69,593	[^3^H]DAMGO	[^3^H]DPDPE	[^3^H](+)‐pentazocine	[^3^H]DTG
(±)‐**2** 12	racemate	5.6±0.6	573	413	2 %	0 %
**3** 19		0.25±0.08	43±9.2	58±8.4	0 %	8 %
(±)‐**4**	racemate	1.2±0.6	1200	1400	0 %	0 %
**4**	4a*R*,8*S*,8a*S*	0.81±0.32	0 %	0 %	676	3300
*ent* **‐4**	4a*S*,8*R*,8a*R*	195±67	0 %	0 %	0 %	12 %
U‐50,488	1*S*,2*S*	0.34±0.07	–	–	–	–
naloxone		7.3±0.40	2.3±1.1	103	–	–
morphine		–	5.2±1.6	–	–	–
SNC80		–	–	1.2±0.5	–	–
(+)‐pentazocine		–	–	–	5.4±0.5	–
haloperidol		–	–	–	6.6±0.9	78±2.3

[a] A value in % reflects the inhibition of the radioligand binding at a test compound concentration of 1 μM. *K*_i_ values without SEM values represent the mean of two experiments (*n*=2) and *K*_i_ values with SEM values represent the mean of three experiments (*n*=3). [b] Guinea pig brain membrane preparations were used in the KOR, MOR and σ_1_ assay. In the DOR assay rat brain and in the σ_2_ assay rat liver membrane preparations were used.

The KOR affinity of racemic (±)‐**4** (*K*_i_=1.2 nM) is approximately five times higher than the KOR affinity of triazole‐based KOR agonist (±)‐**2**, but five times lower than those of enantiomerically pure methyl carbamate **3**. The KOR affinity of (±)‐**4** resides almost exclusively in the 4a*R*,8*S*,8a*S*‐configured enantiomer **4** (*K*_i_=0.81 nM), which is more than 200‐fold more active than the distomer *ent*‐**4**. The *K*_i_ value of the eutomer **4** is in the same range as the *K*_i_ values of the prototypical KOR agonists U‐50,488 and the methyl carbamate **3**. (Table 1)

The enantiomerically pure KOR agonist **4** as well as the racemic mixture (±)‐**4** and the distomer *ent*‐**4** showed very low affinity towards MOR and DOR, indicating high selectivity for KOR over the other two opioid receptor subtypes MOR and DOR. Due to the historical and ligand structure relationship of σ and opioid receptors, in particular KOR, σ_1_ and σ_2_ receptor affinity of the ligands were also recorded.^28^ Very low σ_1_ and σ_2_ affinity was found for the 4a*R*,8*S*,8a*S*‐configured enantiomer **4**. However, the *K*_i_ values at both σ receptors indicate a more than 800‐ (σ_1_) and more than 4000‐fold (σ_2_) selectivity for KOR.

The KOR agonistic activity was investigated for the eutomer **4** with 4a*R*,8*S*,8a*S* configuration. For this purpose, two assays recording the reduction of cAMP production (human HEK293T cells) and β‐arrestin‐2 recruitment (Tango assay using human HTLA cells) were performed (Table 2).

**Table 2:** Correlation of KOR affinity and KOR activity of **4** and U‐50,488.

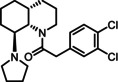					
Compd.	KOR^[a]^ [^3^H]U‐69,593	cAMP^[b]^		β‐Arrestin‐2^[c]^	
	*K*_i_±SEM [nM]	EC_50_ [nM]	*E*_max_ [%]^[d]^	EC_50_ [nM]	*E*_max_ [%]^[d]^
**4**	0.81±0.32	0.029	103	1.09	64
U‐50,488	0.34±0.07	0.16	100	7.85	100

[a] Guinea pig brain membrane preparations. [b] Human HEK 293T cells. [c] Human HTLA cells. [d] The *E*_max_ values refer to U‐50,488 (100 %).

In Table 2, the KOR affinity and agonistic activity of enantiomerically pure **4** and the prototypical KOR agonist U‐50,488 are compared. Whereas the KOR affinity of **4** is slightly lower than the KOR affinity of U‐50,488, its effect on the cAMP production is higher than that of U‐50,488. With an EC_50_ value of 0.029 nM **4** shows very high KOR agonistic activity. In the β‐arrestin‐2 recruitment assay **4** is also approximately seven times more active than U‐50,488. In the cAMP assay **4** reaches the same total effect as U‐50,488 (100 % intrinsic activity), whereas in the β‐arrestin‐2 recruitment assay **4** attains only 64 % of the U‐50,488 effect. These data indicate, that **4** does not prefer the activation of one of the two pathways (no biased activation).

In analogous MOR and DOR cAMP and β‐arretin‐2 recruitment assays, **4** did not exhibit significant activity, indicating the selective activation of these pathways via interaction with KOR.

### Effects of KOR agonists (±)‐4 and its enantiomers 4 and *ent*‐4 on immune cells

To further characterize the anti‐inflammatory and immunomodulatory properties of the newly synthesized compounds different immune cell subsets were isolated including T cells, dendritic cells (DC) or macrophages, which are known to be involved in the development and progression of various (systemic) inflammatory disorders.^29^ Immune cell populations were purified from lymph nodes and spleen of wild‐type (WT) mice or from peripheral blood of healthy human donors, stimulated with lipopolysaccharide (LPS; myeloid cells) or anti‐CD3 plus anti‐CD28 (T cells) to upregulate the expression of typical activation markers and induce the production of pro‐inflammatory cytokines. Upon activation, antigen‐presenting cells, such as DC or macrophages, upregulate co‐stimulatory markers of the tumor‐necrosis factor (TNF) or B7 families including CD40 or CD86, respectively and start to secrete INF‐γ, IL‐6, IL‐12 or TNF‐α. In systemic inflammation, these activated antigen‐presenting cells get into direct contact to naive T cells, resulting in the priming of effector T cells (T helper or cytotoxic T cells). Effector T cells including Th1 or Th17 cells migrate to the site of inflammation and thus, can be found in target organs. Under infectious conditions they are critically involved in the elimination of bacteria or virus‐containing cells. However, in chronic and complex inflammatory diseases, Th1 and Th17 are also considered to mediate tissue destruction as shown in psoriasis, atopic dermatitis, rheumatoid arthritis or multiple sclerosis.^30^

**Figure 6:**
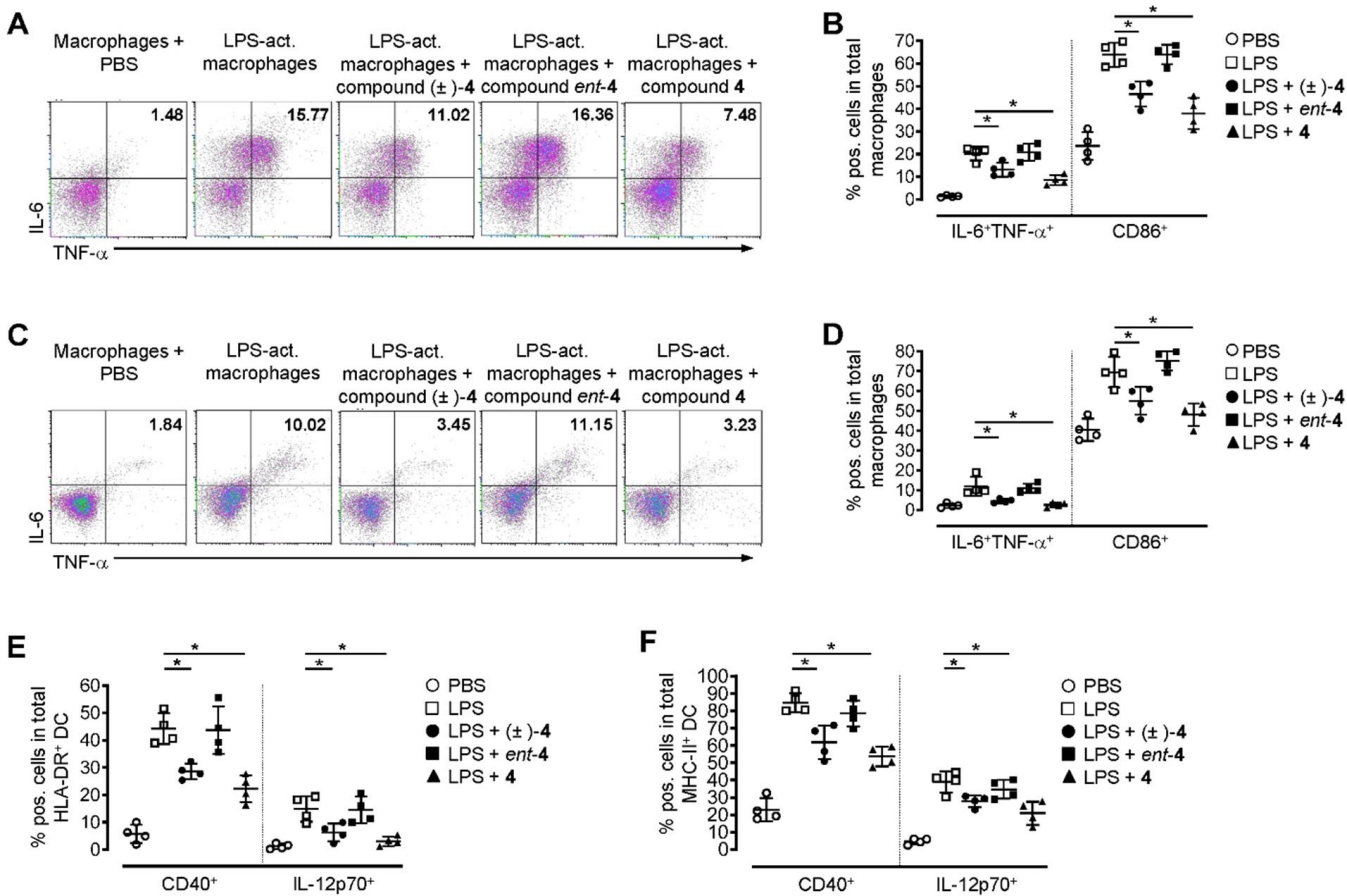
Compounds (±)‐**4** and **4** significantly reduced the activation and cytokine production in myeloid human and mouse immune cells. A), B) Human macrophages were sorted from peripheral blood mononuclear cells (PBMC) by magnetic beads and activated with LPS for 12 h as described (activated macrophages). Subsequently, cells were stimulated with compounds (±)‐**4**, **4** and *ent*‐**4** (1 μM each) for an additional 48 h. Control cells received an equal amount of PBS. A) Representative dot‐plots and B) percentages of cells expressing the pro‐inflammatory cytokines TNF‐α and IL‐6 or the co‐stimulatory surface marker CD86 from *n*=4 healthy human donors are shown. Cells are gated for CD11b^+^CD68^+^ macrophages, and IL‐6 as well as TNF‐α staining was performed after cell permeabilization. Data are presented as mean±SEM; * *p* <0.05. C), D) Mouse macrophages were isolated from lymph node and spleen tissue and stimulated with LPS for 12 h as described (activated macrophages). Subsequently, cells were cultured in the presence of compounds (±)‐**4**, **4** and *ent*‐**4** (1 μM each) for an additional 48 h. Control cells received an equal amount of PBS. C) Representative dot‐plots and D) percentages of cells expressing the pro‐inflammatory cytokines TNF‐α and IL‐6 or the co‐stimulatory surface marker CD86 from *n*=4 individual mice are shown. Cells are gated for CD11b^+^F4/80^+^ macrophages, and IL‐6 as well as TNF‐α staining was performed after cell permeabilization. Data are presented as mean±SEM; * *p* <0.05. E) Human or F) mouse dendritic cells were purified from peripheral blood or spleen tissue, respectively, and stimulated with LPS for 12 h. Subsequently, cells were incubated with compounds (±)‐**4**, **4** and *ent*‐**4** (1 μM each) for an additional 48 h or received an equal amount of PBS. Percentages of cells expressing the pro‐inflammatory cytokine IL‐12 or the co‐stimulatory surface marker CD40 from *n*=4 healthy human donors (E) or *n*=4 individual mice (F) are shown. Cells are gated for HLA‐DR^+^ (E) or MHC‐II^+^F4/80^−^CD19^−^ (F) and IL‐12 staining was performed after cell permeabilization. Data are presented as mean±SEM; * *p* <0.05.

To assess the anti‐inflammatory capacity of the KOR agonists (±)‐**4**, **4** and *ent*‐**4** pre‐activated T cells, macrophages, neutrophils and DC were stimulated with the KOR agonists in a concentration of 1 μM after having confirmed that this concentration is sufficient to markedly downregulate the expression of the early and transient activation marker CD69 (Figure S7). Interestingly, in activated human macrophages we observed significantly reduced numbers of cells expressing the co‐stimulatory receptor CD86 as well as the cytokines IL‐6 and TNF‐α after stimulation with the compounds (±)‐**4** and **4** (Figure 6A and B). In contrast, compound *ent*‐**4** did not show anti‐inflammatory properties since comparable levels of cells expressing co‐stimulatory receptors or pro‐inflammatory cytokines were found in *ent*‐**4** stimulated cultures and controls (Figure 6A and B). Worth mentioning that the potent anti‐inflammatory effect of (±)‐**4** and **4** was not limited to human macrophages since we observed a similar reduction in the expression of typical surface markers characteristic for cell activation and classical pro‐inflammatory cytokines in human DC and mouse myeloid immune cell subsets (Figure 6C–F).

It is well known that opioid receptor agonists not only control the phenotype and function of myeloid cells but also modulate lymphoid immune cell subsets and thereby, impact on the innate as well as the adaptive immune system. In line with this, it has been shown that besides impairing DC maturation, KOR agonists are able to prevent effector T cell priming.^12^, ^31^

**Figure 7:**
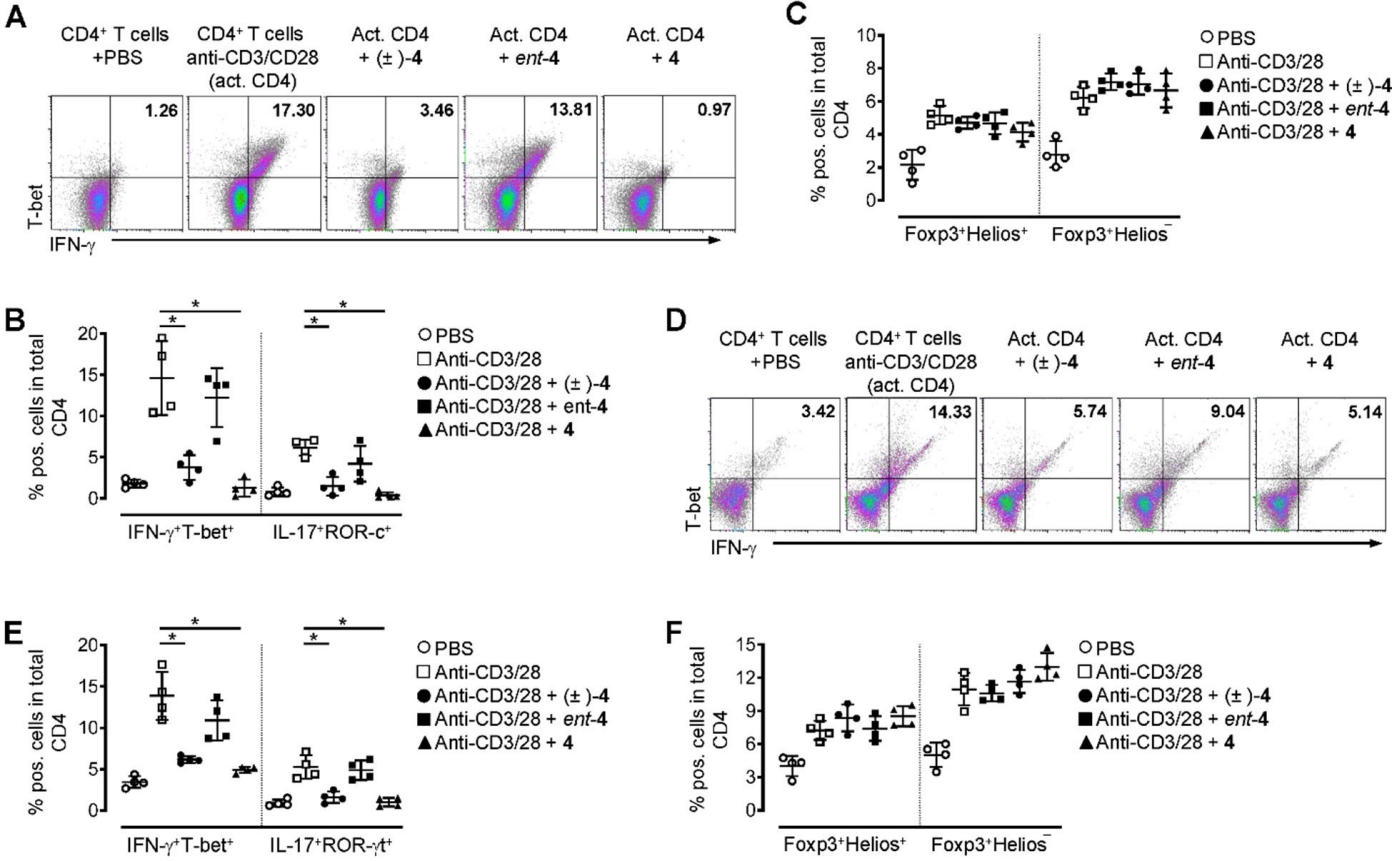
Compounds (±)‐**4** and **4** significantly reduced the cytokine production and transcription factor expression in Th1 and Th17 cells but did not exhibit immunomodulatory capacities. A)–C) Human CD4^+^ T cells were sorted from peripheral blood and activated with anti‐CD3 and anti‐CD28 for 48 h (activated CD4). Subsequently, cells were stimulated with compounds (±)‐**4**, **4** and *ent*‐**4** (1 μM each) for an additional 48 h. Control cells received an equal amount of PBS. A) Representative dot‐plots and percentages of cells expressing B) the Th1 markers IFN‐γ and T‐bet, the Th17 markers IL‐17 and ROR‐c from *n*=4 healthy human donors or C) the Treg markers Foxp3 and Helios are shown. Cytokine and transcription factor staining were performed after cell permeabilization. Data are presented as mean±SEM; * *p* <0.05. D)–F) Mouse CD4^+^ T cells were sorted from spleen and lymph node tissue of wild‐type mice, activated with anti‐CD3 and anti‐CD28 for 48 h (activated CD4), and stimulated with compounds (±)‐**4**, **4** and *ent*‐**4** (1 μM each) for an additional 48 h. D) Representative dot‐plots and percentages of cells expressing E) the Th1 markers IFN‐γ and T‐bet, the Th17 markers IL‐17 and ROR‐γt from *n*=4 individual mice or F) the Treg markers Foxp3 and Helios are shown. Cytokine and transcription factor staining were performed after cell permeabilization. Data are presented as mean±SEM; * *p* <0.05.

To assess the effects of the compounds (±)‐**4**, **4** and *ent*‐**4** during T cell activation and to characterize their potential anti‐inflammatory capacity in more detail we purified CD4^+^ T cells from peripheral blood of healthy human donors or spleen and lymph node tissue of WT mice, activated the cells with anti‐CD3 plus anti‐CD28 and stimulated them with the three KOR agonists. Notably, the numbers of both, Th1 and Th17 cells were significantly downregulated in cultures treated with (±)‐**4** or **4** whereas we did not observe reduced levels of CD4^+^ T cells expressing IFN‐γ and the transcription factor T‐bet (Th1 cells) or IL‐17 and the transcription factor ROR‐c (Th17 cells) in T cell cultures that were stimulated with compound *ent*‐**4** (Figure 7A and B). Of note, the anti‐inflammatory effect of compound **4** was even more pronounced compared to (±)‐**4** and was not limited to human CD4^+^ T cells since we observed a similar reduction of Th1 and Th17 cells in murine cultures (Figure 7D and E). However, although compound **4** (and to a lesser extend also compound (±)‐**4**) markedly downregulated the cytokine secretion and transcription factor expression in activated mouse and human CD4^+^ T cells, both compounds did not exhibit immunomodulatory capacities. Immunomodulation would for instance, require the switch from activated effector to immunosuppressive regulatory CD4^+^ T cells (Treg), which among other markers are characterized by the expression of the transcription factor Foxp3. Neither compound (±)‐**4** nor **4** was able to modulate Foxp3 expression in CD4^+^ T cells (Figure 7C and F). Two subsets of Treg are known; thymus‐derived Treg (tTreg) and peripherally induced Treg (pTreg) that can be generated from conventional CD4^+^ T cells and are converted in peripheral tissues to cells that express Foxp3 and acquire suppressive ability. The transcription factor Helios, a member of the Ikaros transcription factor family, is believed to be a marker of tTreg.^32^ Therefore, we analyzed the Helios^+^ and Helios^−^ Treg subsets separately in T cell cultures stimulated with the compounds (±)‐**4, 4** and *ent*‐**4** but did not detect any significant difference in cell numbers compared to non‐stimulated controls (Figure 7C and F), confirming that (±)‐**4** and **4** show broad and potent anti‐inflammatory effects but none of the κ‐agonists seems to have immunomodulatory properties.

## Conclusion

A diastereoselective synthesis of the *cis*,*trans*‐configured perhydroquinoline‐based KOR agonist (±)‐**4** was developed. Since the racemate (±)‐**4** exhibited high KOR affinity (*K*_i_=1.2 nM), enantiomerically pure KOR agonists **4** and *ent*‐**4** were synthesized by a lipase‐catalyzed kinetic resolution of racemic alcohol (±)‐**8**. The 4a*R*,8*S*,8a*S*‐configured ligand **4** represents the eutomer with a KOR affinity of 0.81 nM. Moreover, **4** shows high selectivity for KOR over MOR, DOR, σ_1_ and σ_2_ receptors. Whereas **4** has slightly lower KOR affinity than the prototypical KOR agonist U‐50,488, it shows higher KOR agonistic activity than U‐50,488 in the cAMP and β‐arrestin‐2 assay. A bias for the cAMP or β‐arrestin‐2 pathway could not be observed.

The 4a*R*,8*S*,8a*S*‐configured KOR agonist **4** showed strong anti‐inflammatory activity. After activation of human macrophages, human dendritic cells and mouse myeloid immune cells, the number of cells expressing co‐stimulatory receptor CD86 and proinflammatory cytokines IL6 and TNF‐α was significantly reduced. The numbers of Th1 and Th17 cells was significantly reduced by incubation of activated CD4^+^ T cells with KOR agonist **4**. Moreover, **4** led to downregulation of the cytokine secretion and transcription factor expression (anti‐inflammatory activity), but did not induce immunomodulatory effects. The enantiomerically pure KOR agonist **4** showed broad and potent anti‐inflammatory activity, but did not induce immunomodulatory effects.

Very interestingly, all these different anti‐inflammatory effects correlate nicely with the KOR affinity of the KOR agonists **4**, (±)‐**4**, and *ent*‐**4**. In general, the racemate (±)‐**4** was slightly less potent than the eutomer **4** and the distomer ent‐**4** did not exhibit anti‐inflammatory effects.

## Experimental Section

### Chemistry, General Methods

Oxygen and moisture sensitive reactions were carried out under nitrogen, dried with silica gel with moisture indicator (orange gel, VWR, Darmstadt, Germany) and in dry glassware (Schlenk flask or Schlenk tube). Thin layer chromatography (TLC): TLC silica gel 60 F_254_ on aluminum sheets (VWR). Flash chromatography (fc): Silica gel 60, 40–63 μm (VWR); parentheses include: diameter of the column (Ø), length of the stationary phase (l), fraction size (v) and eluent. Melting point: Melting point system MP50 (Mettler Toledo, Gießen, Germany), open capillary, uncorrected. MS: MicroTOFQII mass spectrometer (Bruker Daltonics, Bremen, Germany); deviations of the found exact masses from the calculated exact masses were 5 ppm or less; the data were analyzed with DataAnalysis® (Bruker Daltonics). NMR: NMR spectra were recorded in deuterated solvents on Agilent DD2 400 MHz and 600 MHz spectrometers (Agilent, Santa Clara CA, USA); chemical shifts (*δ*) are reported in parts per million (ppm) against the reference substance tetramethylsilane and calculated from the solvent residual peak of the undeuterated solvent; coupling constants are given with 0.5 Hz resolution; assignment of ^1^H and ^13^C NMR signals was supported by 2‐D NMR techniques where necessary.IR: FT/IR IR Affinity®‐1 spectrometer (Shimadzu, Düsseldorf, Germany) using ATR technique. Optical rotation: Polarimeter 341 (Perkin Elmer); 1.0 dm tube; concentration *c* in g/100 mL; *T*=20 °C; wavelength 589 nm (D‐line of Na light); the unit of the specific rotation ([α]TD grad mL dm^−1.^g^−1^) is omitted for clarity. CD spectroscopy: Jasco (Pfungstadt, Germany) J‐815 spectropolarimeter; concentration 1 mg/mL in acetonitrile.

### HPLC methods

HPLC method 1 to determine the purity of compounds: Equipment 1: Pump: L‐7100, degasser: L‐7614, autosampler: L‐7200, UV detector: L‐7400, interface: D‐7000, data transfer: D‐line, data acquisition: HSM‐Software (all from LaChrom, Merck Hitachi); Equipment 2: Pump: LPG‐3400SD, degasser: DG‐1210, autosampler: ACC‐3000T, UV detector: VWD‐3400RS, interface: DIONEX UltiMate 3000, data acquisition: Chromeleon 7 (Thermo Fisher Scientific); column: LiChropher® 60 RP‐select B (5 μm), LiChroCART® 250–4 mm cartridge; flow rate: 1.0 mL/min; injection volume: 5.0 μL; detection at *λ*=210 nm; solvents: A: demineralized water with 0.05 % (*v*/*v*) trifluoroacetic acid, B: acetonitrile with 0.05 % (*v*/*v*) trifluoroacetic acid; gradient elution (% A): 0–4 min: 90 %; 4–29 min: gradient from 90 to 0 %; 29–31 min: 0 %; 31–31.5 min: gradient from 0 to 90 %; 31.5–40 min: 90 %. The purity of all test compounds is greater than 95 %.

HPLC method 2 to determine the enantiomeric purity of alcohols (*R*)‐**8** and (*S*)‐**8**: Equipment: UV/Vis detector: L‐7400, pump: L‐7150 A, autosampler: L‐7200, data acquisition: HSM‐software (LaChrom, Merck‐Hitachi); column: Daicel Chiralpack® AD−H (250x4.6 mm, 5 μm particle size); flow rate: 1.0 mL min^−1^; injection volume: 10 μL; detection at *λ*=266 nm; solvent: isohexane/CH_3_OH (95 : 5), isocratic.

HPLC method 3 to determine the enantiomeric purity of acetates (*R*)‐**7** and (*S*)‐**7**: Equipment: UV/Vis detector: L‐7400, pump: L‐7150 A, autosampler: L‐7200, data acquisition: HSM‐software (LaChrom, Merck‐Hitachi); column: Daicel Chiralpack® IA (250x4.6 mm, 5 μm particle size); flow rate: 1.0 mL min^−1^; injection volume: 10 μL; detection at *λ*=266 nm; solvent: isohexane/CH_3_OH (95 : 5), isocratic.

HPLC method 4 to determine the enantiomeric purity of KOR‐agonists **4** and *ent*‐**4**: Equipment: DAD detector: L‐7455, pump: L‐6200 A, injector: Rheodyne 7725i, data acquisition: HSM‐software (LaChrom, Merck‐Hitachi); column: Daicel Chiralpack® IA (250x4.6 mm, 5 μm particle size); flow rate: 1.0 mL min^−1^; injection volume: 5 μL; detection at *λ*=275 nm; solvent: isohexane/isopropyl alcohol (90 : 10)+0.1 % diethylamine, isocratic.

HPLC method 5 to separate the KOR‐agonists **4** and *ent*‐**4** on a preparative scale: Equipment: UV/Vis detector: L‐7400, pump: L‐7150A, autosampler: L‐7200, data acquisition: HSM‐software (LaChrom, Merck‐Hitachi); column: Daicel Chiralpack® IA (250×20 mm, 5 μm particle size); flow rate: 15 mL min^−1^; injection volume: 400 μL; detection at *λ*=275 nm; solvent: isohexane/ ethanol (90 : 10)+0.1 % diethylamine, isocratic.

### Synthetic procedures

*(4a*RS*,8*SR*,8a*SR*)‐8‐(Pyrrolidin‐1‐yl)‐decahydroquinoline* 33 *((±)‐**13**)*

Pyrrolidine (0.34 mL, 4.14 mL, 6.0 equiv) was added to a solution of (±)‐**12** (150 mg, 0.69 mmol, 1.0 equiv) in dry acetonitrile (1.5 mL). After stirring for 18 h at 80 °C, additional pyrrolidine (0.17 mL, 2.07 mmol, 3.0 equiv) was added and the mixture was stirred for another 2 h at 80 °C. The solvent was removed *in vacuo*, toluene was added and evaporated *in vacuo* several times (azeotropic evaporation of excess pyrrolidine). After addition of HCl (1 M, 1 mL), the mixture was washed with Et_2_O (2×2 mL). The pH value of the aqueous layer was then set to pH 10 by addition of NaOH (1 M, 1 mL) and extracted with Et_2_O (2×2 mL). After additional extraction with EtOAc (5×2 mL) the combined organic layers were dried (Na_2_SO_4_), filtered and evaporated *in vacuo*. The residue was purified by flash column chromatography (*d*=2 cm, *h*=20 cm, *V*=10 mL, CH_2_Cl_2_/CH_3_OH (98 : 2→95 : 5) and then CH_2_Cl_2_/CH_3_OH/Et_3_N (94 : 5 : 1)), *R*_f_=0.11 (CH_2_Cl_2_/CH_3_OH 95 : 5). Yellow viscous oil, yield 118 mg (82 %). Chemical formula: C_13_H_24_N_2_ (208.35 g/mol).

*(4a*R,*8*S,*8a*S*)‐8‐(Pyrrolidin‐1‐yl)‐decahydroquinoline (**13**)*

Oxathiazole **12** (188 mg, 0.97 mmol, 1.0 equiv) was treated with pyrrolidine (0.64 mL, 7.79 mmol, 8.0 equiv) in CH_3_CN (2 mL) as described for (±)‐**13**. Dark yellow oil, yield 107 mg (53 %). Specific rotation: [α]20D =+26.6 (*c*=3.2 mg/mL, CH_3_OH).

*(4a*S*,8*R*,8a*R*)‐8‐(Pyrrolidin‐1‐yl)‐decahydroquinoline (ent‐**13**)*

Oxathiazole *ent*‐**12** (205 mg, 0.94 mmol, 1.0 equiv) was treated with pyrrolidine (0.47 mL, 5.72 mmol, 6.1 equiv) in CH_3_CN (2 mL) as described for (±)‐**13**. Dark yellow oil, yield 90 mg (46 %). Specific rotation: [α]20D =−16.9 (*c*=3.4 mg/mL, CH_3_OH).

*2‐(3,4‐Dichlorophenyl)‐1‐[(4aRS,8*SR*,8a*SR*)‐8‐(pyrrolidin‐1‐yl)‐3,4,4a,5,6,7,8,8a‐octahydroquinolin‐1(2*H*)‐yl]ethan‐1‐one* 33 *((±)‐**4**)*

Ethyldiisopropylamine (0.09 mL, 0.53 mmol, 2.0 equiv) was added to a solution of (±)‐**13** (57 mg, 0.27 mmol, 1.0 equiv) in dry CH_2_Cl_2_ (2.5 mL). 2‐(3,4‐Dichlorophenyl)acetyl chloride (122 mg, 0.55 mmol, 2.0 equiv) dissolved in dry CH_2_Cl_2_ (0.5 mL) was added dropwise at 0 °C. The mixture was stirred for 1 h at room temperature. After addition of H_2_O (3.0 mL), the mixture was extracted with CH_2_Cl_2_ (3×5 mL). The combined organic layers were dried (Na_2_SO_4_), filtered and concentrated *in vacuo*. The residue was purified by flash column chromatography (*d*=1 cm, *h*=15 cm, *V*=5 mL, CH_2_Cl_2_/CH_3_OH (95 : 5), *R*_f_=0.31 (CH_2_Cl_2_/CH_3_OH 95 : 5)). Yellow solid, mp 83 °C, yield 61 mg (56 %). Chemical formula: C_21_H_28_Cl_2_N_2_O (395.4 g/mol).

*2‐(3,4‐Dichlorophenyl)‐1‐[(4a*R*,8*S*,8a*S*)‐8‐(pyrrolidin‐1‐yl)‐3,4,4a,5,6,7,8,8a‐octahydroquinolin‐1(2*H*)‐yl]ethan‐1‐one (**4**)*

Secondary amine **4** (81 mg, 0.39 mmol, 1.0 equiv) was treated with ethyldiisopropylamine (0.13 mL, 0.76 mmol, 1.9 equiv) and 2‐(3,4‐dichlorophenyl)acetyl chloride (174 mg, 0.78 mmol, 2.0 equiv) in dry CH_2_Cl_2_ (3 mL) as described for (±)‐**4**. The product was obtained as a yellow oil with a yield of 110 mg (71 %, 80.3 %*ee* (HPLC method 4)). Therefore, a purification by chiral HPLC (HPLC method 5) was performed. Yellow oil, yield 63 mg (41 %). Purity (HPLC method 1): 96.8 % (*t*_R_=18.96 min). Enantiomeric purity (HPLC, method 4): 99.8 % *ee* (*t*_R_=7.15 min). Specific rotation: [α]20D =−22.6 (*c*=6.4 mg/mL, CH_3_OH).

*2‐(3,4‐Dichlorophenyl)‐1‐[(4a*S*,8*R*,8a*R*)‐8‐(pyrrolidin‐1‐yl)‐3,4,4a,5,6,7,8,8a‐octahydroquinolin‐1(2*H*)‐yl]ethan‐1‐one (ent‐**4**)*

Secondary amine *ent*‐**13** (67 mg, 0.32 mmol, 1.0 equiv) was treated with ethyldiisopropylamine (0.11 mL, 0.65 mmol, 2.0 equiv) and 2‐(3,4‐dichlorophenyl)acetyl chloride (144 mg, 0.64 mmol, 2.0 equiv) in dry CH_2_Cl_2_ (3 mL) as described for (±)‐**4**. The product was obtained as a yellow oil with a yield of 71 mg (56 %, 66.3 % *ee* (HPLC method 4)). Therefore, a purification by chiral HPLC (HPLC method 5) was performed. Yellow oil, yield 37 mg (29 %). Purity (HPLC method 1): 97.2 % (*t*_R_=19.02 min). Enantiomeric purity (HPLC method 4): 99.0 % *ee* (*t*_R_=8.75 min). Specific rotation: [α]20D =+23.3 (*c*=3.5 mg/mL, CH_3_OH).

### Receptor binding studies

#### Materials

Guinea pig brains and rat brains were commercially available (Harlan‐Winkelmann, Borchen, Germany). Homogenizers: Elvehjem Potter (B. Braun Biotech International, Melsungen, Germany) and Soniprep® 150 (MSE, London, UK). Centrifuges: Cooling centrifuge model Eppendorf 5427R (Eppendorf, Hamburg, Germany) and High‐speed cooling centrifuge model Sorvall® RC‐5 C plus (Thermo Fisher Scientific, Langenselbold, Germany). Multiplates: standard 96 well multiplates (Diagonal, Muenster, Germany). Shaker: self‐made device with adjustable temperature and tumbling speed (scientific workshop of the institute). Harvester: MicroBeta® FilterMate 96 Harvester. Filter: Printed Filtermat Typ A and B. Scintillator: Meltilex® (Typ A or B) solid state scintillator. Scintillation analyzer: MicroBeta® Trilux (all Perkin Elmer LAS, Rodgau‐Jügesheim, Germany).

#### Preparation of membrane homogenates from guinea pig brain

Five guinea pig brains were homogenized with the potter (500–800 rpm, 10 up and down strokes) in 6 volumes of cold 0.32 M sucrose. The suspension was centrifuged at 1200 *g* for 10 min at 4 °C. The supernatant was separated and centrifuged at 23 500 *g* for 20 min at 4 °C. The pellet was resuspended in 5–6 volumes of buffer (50 mM Tris, pH 7.4) and centrifuged again at 23 500 *g* (20 min, 4 °C). This procedure was repeated twice. The final pellet was resuspended in 5–6 volumes of buffer and frozen (−80 °C) in 1.5 mL portions containing about 1.5 mg protein/mL.

#### Preparation of membrane homogenates from rat brain

Five rat brains (species: Sprague Dawley rats) were homogenized with the potter (500–800 rpm, 10 up and down strokes) in 6 volumes of cold 0.32 M sucrose. The suspension was centrifuged at 1200 *g* for 10 min at 4 °C. The supernatant was separated and centrifuged at 23 500 *g* for 20 min at 4 °C. The pellet was resuspended in 5–6 volumes of buffer (50 mM Tris, pH 7.4) and centrifuged again at 23 500 *g* (20 min, 4 °C). This procedure was repeated twice. The final pellet was resuspended in 5–6 volumes of buffer and frozen (−80 °C) in 1.5 mL portions containing about 1.5 mg protein/mL.

#### Protein determination

The protein concentration was determined by the method of Bradford,^34^ modified by Stoscheck.^35^ The Bradford solution was prepared by dissolving 5 mg of Coomassie Brilliant Blue G 250 in 2.5 mL of EtOH (95 %, *v*/*v*). 10 mL deionized H_2_O and 5 mL phosphoric acid (85 %, *m*/*v*) were added to this solution, the mixture was stirred and filled to a total volume of 50 mL with deionized water. The calibration was carried out using bovine serum albumin as a standard in 9 concentrations (0.1, 0.2, 0.4, 0.6, 0.8, 1.0, 1.5, 2.0 and 4.0 mg /mL). In a 96‐well standard multiplate, 10 μL of the calibration solution or 10 μL of the membrane receptor preparation were mixed with 190 μL of the Bradford solution, respectively. After 5 min, the UV absorption of the protein‐dye complex at *λ*=595 nm was measured with a plate reader (Tecan Genios®, Tecan, Crailsheim, Germany).

#### General procedures for the binding assays

The test compound solutions were prepared by dissolving approximately 10 μmol (usually 2–4 mg) of test compound in DMSO so that a 10 mM stock solution was obtained. To obtain the required test solutions for the assay, the DMSO stock solution was diluted with the respective assay buffer. The filter mats were presoaked in 0.5 % aqueous polyethylenimine solution for 2 h at RT before use. All binding experiments were carried out in duplicates in the 96 well multiplates. The concentrations given are the final concentration in the assay. Generally, the assays were performed by addition of 50 μL of the respective assay buffer, 50 μL of test compound solution in various concentrations (10^−5^, 10^−6^, 10^−7^, 10^−8^, 10^−9^ and 10^−10^ mol/L), 50 μL of the corresponding radioligand solution and 50 μL of the respective receptor preparation into each well of the multiplate (total volume 200 μL). The receptor preparation was always added last. During the incubation, the multiplates were shaken at a speed of 500–600 rpm at the specified temperature. Unless otherwise noted, the assays were terminated after 120 min by rapid filtration using the harvester. During the filtration, each well was washed five times with 300 μL of water. Subsequently, the filter mats were dried at 95 °C. The solid scintillator was melted on the dried filter mats at a temperature of 95 °C for 5 min. After solidifying of the scintillator at RT, the trapped radioactivity in the filter mats was measured with the scintillation analyzer. Each position on the filter mat corresponding to one well of the multiplate was measured for 5 min with the [^3^H]‐counting protocol. The overall counting efficiency was 20 %. The IC_50_ values were calculated with the program GraphPad Prism® 3.0 (GraphPad Software) by nonlinear regression analysis. Subsequently, the IC_50_ values were transformed into *K*_i_ values using the equation of Cheng and Prusoff.^36^ The *K*_i_ values are given as mean value±SEM from three independent experiments.

#### KOR assay

The assay was performed with the radioligand [^3^H]U‐69,593 (55 Ci/mmol, BIOTREND). The thawed guinea pig brain membrane preparation (about 100 μg of the protein) was incubated with various concentrations of test compounds, 1 nM [^3^H]U‐69,593, and Tris‐MgCl_2_ buffer (50 mM Tris, 8 mM MgCl_2_, pH 7.4) at 37 °C. The nonspecific binding was determined with 10 μM unlabeled U‐69,593. The *K*_d_ value of U‐69,593 is 0.69 nM.^27^

#### MOR assay

The assay was performed with the radioligand [^3^H]DAMGO (51 Ci/mmol, Perkin Elmer). The thawed guinea pig brain membrane preparation (about 100 μg of the protein) was incubated with various concentrations of test compounds, 3 nM [^3^H]DAMGO, and Tris‐MgCl_2_ buffer (50 mM Tris, 8 mM MgCl_2_, pH 7.4) at 37 °C. The nonspecific binding was determined with 10 μM unlabeled naloxone. The *K*_d_ value of DAMGO is 0.57 nM.^26^

#### DOR assay

The assay was performed with the radioligand [^3^H]DPDPE (69 Ci/mmol, BIOTREND). The thawed rat brain membrane preparation (about 75 μg of the protein) was incubated with various concentrations of test compounds, 3 nM [^3^H]DPDPE, and Tris‐MgCl_2_ buffer (50 mM Tris, 8 mM MgCl_2_, pH 7.4) supplemented with SIGMAFAST® protease inhibitor mix (Sigma Aldrich Biochemicals, Hamburg, Germany; 1 tablet dissolved in 100 mL of buffer) at 37 °C. The nonspecific binding was determined with 10 μM unlabeled morphine. The *K*_d_ value of DPDPE is 0.65 nM.^25^

#### σ_1_ receptor assay

The assay was performed with the radioligand [^3^H]‐(+)‐Pentazocine (22.0 Ci/mmol; Perkin Elmer). The thawed membrane preparation of guinea pig brain cortex (about 100 μg of protein) was incubated with various concentrations of test compounds, 2 nM [^3^H]‐(+)‐Pentazocine, and Tris buffer (50 mM, pH 7.4) at 37 °C. The nonspecific binding was determined with 10 μM unlabeled (+)‐Pentazocine. The *K*_d_ value of (+)‐Pentazocine is 2.9 nM.^37^

#### σ_2_ receptor assay

The assays were performed with the radioligand [^3^H]DTG (specific activity 50 Ci/mmol; ARC, St. Louis, MO, USA). The thawed membrane preparation of rat liver (about 100 μg of protein) was incubated with various concentrations of the test compound, 3 nM [^3^H]DTG and buffer containing (+)‐pentazocine (500 nM (+)‐pentazocine in 50 mM Tris, pH 8.0) at room temperature. The nonspecific binding was determined with 10 μM unlabeled DTG. The *K*_d_ value of [^3^H]DTG is 17.9 nM.^38^

### *In vitro* functional assays

#### cAMP inhibition assay

To measure KOR G_αi_‐mediated cAMP inhibition, HEK 293T (ATCC CRL‐11268) cells were co‐transfected with human KOR along with a luciferase‐based cAMP biosensor (GloSensor; Promega) and assays were performed similar to previously described.^39^ After 16 h, transfected cells were plated into Poly‐lysine coated 384‐well white clear bottom cell culture plates with DMEM+1 % dialyzed FBS at a density of 15 000–20 000 cells per 40 μL per well and incubated at 37 °C with 5 % CO_2_ overnight. The next day, drug solutions were prepared in fresh drug buffer (20 mM HEPES, 1× HBSS, 0.3 % bovine serum albumin (BSA), pH 7.4) at 3× drug concentration. Plates were decanted and received 20 μL per well of drug buffer (20 mM HEPES, 1× HBSS) followed by addition of 10 μL of drug solution (3 wells per condition) for 15 min in the dark at room temperature. To stimulate endogenous cAMP via β adrenergic‐G_s_ activation, 10 μL luciferin (4 mM final concentration) supplemented with isoproterenol (400 nM final concentration) were added per well. Cells were again incubated in the dark at room temperature for 15 min, and luminescence intensity was quantified using a Wallac TriLux microbeta (Perkin Elmer) luminescence counter. Results (relative luminescence units) were plotted as a function of drug concentration, normalized to % SalA stimulation, and analyzed by using “log(agonist) vs. response” in GraphPad Prism 5.0.

#### Tango β‐arrestin‐2 recruitment assay

The KOR Tango constructs were designed and assays were performed as previously described.^40^ HTLA cells expressing TEV fused‐β‐arrestin2 (kindly provided by Dr. Richard Axel, Columbia University) were transfected with the KOR Tango construct. The next day, cells were plated in DMEM supplemented with 1 % dialyzed FBS in poly‐L‐lysine coated 384‐well white clear bottom cell culture plates at a density of 10 000–15 000 cells/well in a total of 40 μL. The cells were incubated for at least 6 h before receiving drug stimulation. Drug solutions were prepared in drug buffer (20 mM HEPES, 1× HBSS, 0.3 % BSA, pH 7.4) at 3× and added to cells (20 μL per well) for overnight incubation. Drug solutions used for the Tango assay were exactly the same as used for the cAMP assay. The next day, media and drug solutions were removed and 20 μL per well of BrightGlo reagent (purchased from Promega, after 1 : 20 dilution) was added. The plate was incubated for 20 min at room temperature in the dark before being counted using a luminescence counter. Results (relative luminescence units) were plotted as a function of drug concentration, normalized to % SalA stimulation, and analyzed using “log(agonist) vs. response” in GraphPad Prism 5.0.

### Immune cell isolation and multicolor flow cytometry

#### Purification and stimulation of mouse immune cell populations

C57BL/6 mice (wild‐type, WT; purchased from Janvier Labs, Le Genest‐Saint‐Isle, France) were used at the age of 8 to 12 weeks and housed under specific pathogen–free (SPF) conditions in microisolator cages. Mice were given chow and water *ad libitum* and animal experiments were performed with the approval of the State Review Board of North Rhine‐Westphalia according to the German law for animal welfare (reference number 81–02.05.50.17.015). After sacrifice of mice spleen and peripheral lymph nodes were removed to isolate T cells, macrophages or DC. For this purpose, single cell suspensions of the tissues were prepared according to standard methods. Subsequently, total T cells were isolated from using the Pan T Cell Isolation Kit II, DC were purified using the Pan Dendritic Cell Isolation Kit mouse, macrophages were separated from single cell suspensions with anti‐F4/80 microbeads (all purchased from Miltenyi Biotech, Bergisch Gladbach, Germany), activated, stimulated with the compounds (±)‐**4**, **4** and *ent*‐**4**, and subjected to flow cytometry analyses (see below). After isolation, mouse macrophages or DC were activated for 12 h with **LPS** (10 μg/mL) and cultured for additional 48 h in the presence of compounds (±)‐**4**, **4** and *ent*‐**4** at indicated concentrations or PBS as a control. Mouse T cells were activated with plate‐bound anti‐CD3 and soluble anti‐CD28 (clones 145–2 C11 and 37.51; 0.5 μg/mL each antibody, both purchased from Biolegend, San Diego, CA). Finally, cells were subjected to flow cytometry (see below).

#### Purification and stimulation of human immune cells

Human macrophages, DC and T cells were isolated from peripheral blood mononuclear cells (PBMC). Therefore, PBMC were purified from fully anonymized leukapheresis reduction chambers, obtained from the blood bank with the informed consent of healthy donors by Ficoll gradient centrifugation according to standard methods (Ficoll reagent was purchased from Merck). Total macrophages, DC or T cells were selected using CD14 microbeads (macrophages) or negatively enriched using the Pan‐DC Enrichment Kit or the Pan T Cell Isolation Kit (Miltenyi Biotech). All experiments were carried out according to the declaration of Helsinki and were approved by the ethical committee of the University of Münster Medical School (2008‐180‐f‐S). After isolation, human macrophages or DC were activated for 12 h with **LPS** (10 μg/mL) and cultured for additional 48 h in the presence of compounds (±)‐**4**, **4** and *ent*‐**4** at indicated concentrations or PBS. Human T cells were activated with plate‐bound anti‐CD3 and soluble anti‐CD28 (clones UCHT1 and CD28.2; 1 μg/mL each antibody, both purchased from Biolegend, San Diego, CA). Finally, cells were subjected to flow cytometry.

#### Multicolor flow cytometry analyses

The expression of cell surface and intracellular markers was analyzed by multicolor flow cytometry on a Gallios^TM^ flow cytometer (Beckman Coulter) using the Kaluza software. For flow cytometry, mouse cells were stained in PBS using antibodies against CD4 (clone RM4‐5), CD11b (clone M1/ 70), CD86 (clone GL1), MHC II (major histocompatibility complex II; clone M5/114), CD69 (clone H1.2F3), F4/80 (clone BM8), CD40 (clone 3/23), and CD19 (clone 6D5; all purchased from Biolegend). Intracellular staining of Foxp3 (clone FJK‐16s), Helios (clone 22F6), IFN‐γ (clone XMG1.2), IL‐12 (clone C17.8), IL‐17 (clone TC11‐18H10.0), ROR‐γt (clone REA278), T‐bet (clone 4B10), IL‐6 (clone MP5‐20F3), and TNF‐α (clone MP6‐XT22; all purchased from Biolegend or Miltenyi Biotech) was performed after cell permeabilization using the Fix/Perm Buffer Set (Biolegend) according to the manufacturer's instructions.

Human cells were stained in PBS using antibodies against CD4 (clone OKT4), CD11b (clone M1/ 70), CD86 (clone BU63), CD40 (clone 5 C3), and HLA‐DR (clone L243; all purchased from Biolegend). Intracellular staining of Foxp3 (clone 206D), Helios (clone 22F6), IFN‐γ (clone 4S.B3), IL‐12 (clone C11.5), IL‐17 (clone BL168), ROR‐c (clone REA278), T‐bet (clone 4B10), IL‐6 (clone MQ2‐13 A5), and TNF‐α (clone Mab11; all purchased from Biolegend or Miltenyi Biotech) was performed after cell permeabilization using the Fix/Perm Buffer Set (Biolegend) according to the manufacturer's instructions. Isotype‐matched controls were included in each staining, and apoptotic cells were identified using an annexin V apoptosis detection kit.

#### Statistics

All values are expressed as means±SEM. Statistically significant differences were assessed by one‐way analysis of variance (ANOVA) test, comparing more than two groups. The alpha‐level was set at a value of <0.05 in all cases. SigmaPlot 14 or GraphPad Prism 8 was used to analyze, plot, and illustrate data.

#### Computational methods – Simulation of electronic CD spectra

A 3D molecular model of (*S*)‐**14** was built with the Molecular Operating Environment (MOE) software (version 2018.0101).^41^ The structure was then energy minimized using the MMFF94x force field. Compound (*S*)‐**14** was then subjected to a conformational search using the low mode molecular dynamics (LowMD) method and the MMFF94x force field, as implemented in MOE, with an energy window of 5 kcal/mol. The structures of the generated conformers (six low‐energy conformers) were stepwise‐optimized in Gaussian 03.^42^ During the first step, the AM1 semi‐empirical method was employed. Calculation at higher level of theory using the B3LYP density functional and a 6–31G(d,p) basis set constituted the second optimization step. The population of the resulting conformers were calculated from their final total energies by a Boltzmann distribution. TDDFT calculations were performed for the first two conformers (representing 95 % of the population) using the same density functional and basis set as above, considering the 30 lowest‐energy electronic transitions for each conformer. All calculations were run in vacuo. The ECD curve, expressed as the differential molar extinction, Δϵ (in M^−1^ cm^−1^) as function of the energy, *E* (in eV), was obtained by applying a Gaussian‐type function^43^ in Microsoft Excel over the corresponding rotatory strength vectors (length). A width of the band at 1/e height of 0.15 eV was chosen. The final spectrum was obtained as a weighted average of the spectra of two conformers according to the calculated Boltzmann distribution, applying a blue‐shift of 0.55 eV.

## Supporting Information

The Supporting Information contains the scheme for the synthesis of enantiomerically pure KOR agonists **4** and *ent*‐**4**, procedures and analytical data of synthesized compounds, HPLC chromatograms of enantiomerically pure acetates **7**, alcohols **8** and KOR agonists **4**, effects of (±)‐**4** on CD4^+^ T cells and ^1^H and ^13^C NMR spectra of all prepared compounds.

## Conflict of interest

The authors declare no conflict of interest.

## Supporting information

As a service to our authors and readers, this journal provides supporting information supplied by the authors. Such materials are peer reviewed and may be re‐organized for online delivery, but are not copy‐edited or typeset. Technical support issues arising from supporting information (other than missing files) should be addressed to the authors.

Supplementary
