# Supplementary material for: Enantiomerically Pure Quinoline‐Based κ‐Opioid Receptor Agonists: Chemoenzymatic Synthesis and Pharmacological Evaluation
Source: ChemMedChem. 2020 Jul 2;15(15):1408–20. doi: 10.1002/cmdc.202000300 (PMC7496650; doi:10.1002/cmdc.202000300)
Supplement: Supplementary file 1 — Supplementary [file CMDC-15-1408-s001.pdf]

ChemMedChem

Supporting Information

Enantiomerically Pure Quinoline-Based κ-Opioid Receptor Agonists: Chemoenzymatic Synthesis and Pharmacological Evaluation

Benedikt Martin, Dirk Schepmann, Freddy A. Bernal, Thomas J. Schmidt, Tao Che, Karin Loser, and Bernhard Wünsch*

Content

1. Synthesis of enantiomerically pure KOR agonists **4** and *ent*-**4** S2

2. Procedures and analytical data of synthesized compounds S3

3. Chiral HPLC chromatograms of **7**, **8** and KOR agonists **4** S12

4. Effect of (±)-**4** on $CD4^+$ T cell S18

5. ^1H and ^{13}C NMR spectra S19

1. Synthesis of enantiomerically pure KOR agonists 4 and *ent*-4

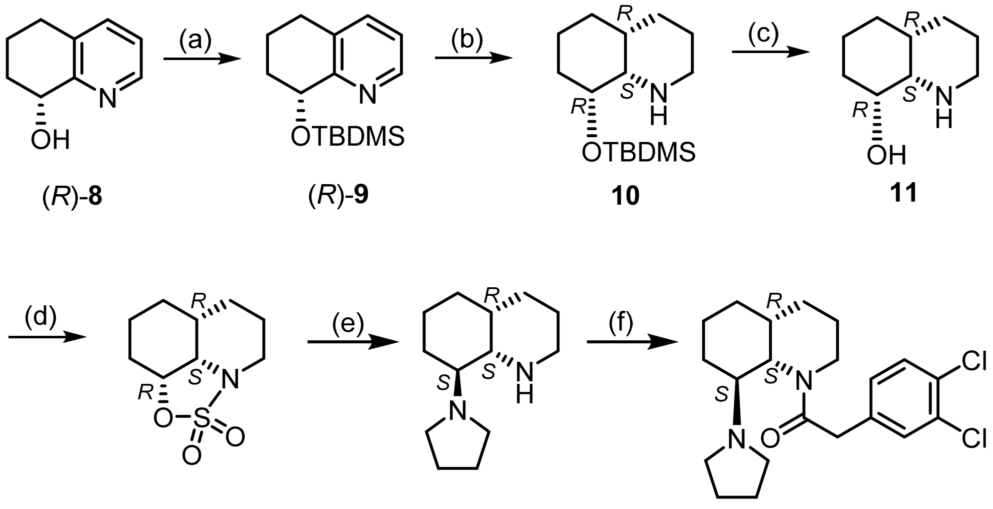

(*R*)-**8** (*R*)-**9** **10** **11**

12 **13** **4**

Scheme S1: Synthesis of enantiomerically pure KOR agonist **4**. Reagents and reaction conditions: (a) TBDMSCl, imidazole, DMF, rt, 18 h, 91 %; (b) H_2 (5 bar), Rh/Al_2O_3, AcOH, rt, 23 h, 120 %; (c) TBAF, THF, rt, 20 h, 92 %; (d) SO_2Cl_2, triethylamine, CH_2Cl_2, 0 °C → rt, 19 h, 30 %; (e) pyrrolidine, CH_3CN, 80 °C, 18 h, 53 %; (f) 2-(3,4-dichlorophenyl)acetyl chloride, ethyldi(isopropyl)amine, CH_2Cl_2, rt, 1 h, 41 %.

The enantiomer *ent*-**4** was prepared in the same manner starting with (*S*)-configured alcohol (*S*)-**8**. The following yields were obtained: (*S*)-**9**: 91 %, *ent*-**10**: 96 %; *ent*-**11**: 86 %; *ent*-**12**: 35 %; *ent*-**13**: 46 %; *ent*-**4**: 29 %.

2. Procedures and analytical data of synthesized compounds

5,6,7,8-Tetrahydroquinolin-1-oxide[35] (6)

5,6,7,8-Tetrahydroquinoline (1.5 mL, 11.6 mmol, 1.0 eq) was dissolved in dry CH_2Cl_2 (95 mL) and *m*-chloroperoxybenzoic acid (77 %, 3.0 g, 13.4 mmol, 1.2 eq) was added. The mixture was stirred for 19 h at room temperature. Saturated solutions of $Na_2S_2O_3$ (20 mL) and $NaHCO_3$ (20 mL) and H_2O (20 mL) were added. The layers were separated, and the aqueous layer was extracted with CH_2Cl_2 (3 x 60 mL). The combined organic layers were dried (Na_2SO_4) and the solvent was removed *in vacuo*. The crude product was purified by flash column chromatography (d = 5 cm, h = 17 cm, V = 30 mL, CH_2Cl_2/MeOH = 98:2 → 95:5, R_f = 0.09 (CH_2Cl_2/CH_3OH = 98:2)). Colorless solid, mp 75 °C, yield 1.4 g (81 %). Chemical formula: $C_9H_{11}NO$ (149.2 g/mol). ^{1}H NMR (600 MHz, $CDCl_3$)**:** δ [ppm] = 1.76 - 1.81 (m, 2 H, 7-*H*), 1.88 - 1.94 (m, 2 H, 6-*H*), 2.80 (t, *J* = 6.3 Hz, 2 H, 8-*H*), 2.99 (t, *J* = 6.7 Hz, 2 H, 5-*H*), 7.09 - 7.16 (m, 2 H, 3-H, 4-*H*), 8.28 (d, *J* = 6.0 Hz, 1 H, 2-*H*).^{13}C NMR (151 MHz, $CDCl_3$)**:** δ [ppm] = 21.7 (1 C, C-7), 21.8 (1 C, C-6), 24.9 (1 C, C-5), 28.7 (1 C, C-8), 122.4 (1 C, C-4), 128.9 (1 C, C-3), 137.1 (1 C, C-4a), 137.3 (1 C, C-2), 149.4 (1 C, C-8a). IR (neat)**:** $\tilde{\nu}$ [cm^{-1}] = 2940 (C-H, aliphatic), 787 and 671 (1,2-disubst. arom.). Exact mass (APCI)**:** *m/z* = 150.0927, calculated 150.0913 for $C_9H_{12}NO$ $[M+H]^+$. Purity (HPLC method 1): 96.9 % (t_R = 12.45 min).

5,6,7,8-Tetrahydroquinolin-8-yl acetate ((±)-7)[36]

N-Oxide **6** (11.61 g, 77.8 mmol, 1.0 eq) was dissolved in Ac_2O (200 mL) and the solution was stirred for 1 h at 120 °C. After removal of Ac_2O *in vacuo*, the crude product was purified by flash column chromatography (d = 8 cm, h = 14 cm, V = 60 mL, CH_2Cl_2/MeOH = 98:2 → 91:9, R_f = 0.43 (CH_2Cl_2/CH_3OH = 95:5)). Yellow oil, yield 13.1 g (88 %). Chemical formula: $C_{11}H_{13}NO_2$ (191.2 g/mol).^{1}H NMR (600 MHz, $CDCl_3$)**:** δ [ppm] = 1.80 - 1.87 (m, 1 H, 6-*H*), 1.90 - 1.98 (m, 1 H, 6-*H*), 2.05 (dddd, *J* = 14.0/10.9/4.6/3.1 Hz, 1 H, 7-*H*), 2.11 (s, 3 H, C*H*$_3$), 2.12 - 2.18 (m, 1 H, 7-*H*), 2.76 (ddd, *J* = 16.8/9.4/5.6 Hz, 1 H, 5-*H*), 2.86 (dt, *J* = 16.9/5.3 Hz, 1 H, 5-*H*), 5.97 (t, *J* = 4.7 Hz, 1 H, 8-*H*), 7.16 (dd, *J* = 7.7/4.7 Hz, 1 H, 3-*H*), 7.45 (d, *J* = 7.8 Hz, 1 H, 4-*H*), 8.50 (dd, *J* = 4.7/0.9 Hz, 1 H, 2-*H*). ^{13}C NMR (151 MHz, $CDCl_3$)**:** δ [ppm] = 18.5 (1 C, C-6), 21.6 (1 C, C*H*$_3$), 28.5 (1 C, C-5), 29.1 (1 C, C-7), 71.0 (1 C, C-8), 123.2 (1 C, C-3), 133.8 (1 C, C-4a), 137.4 (1 C, C-4), 147.9 (1 C, C-2), 153.4 (1 C, C-8a), 170.6 (*C*=O). IR (neat)**:** $\tilde{\nu}$ [cm^{-1}] = 2940 (C-H, aliphatic), 1728 (C=O). Exact mass

(APCI): *m/z* = 192.1043, calculated 192.1019 for $C_{11}H_{14}NO_2$ $[M+H]^+$. Purity (HPLC method 1): 86.7 % (t_R = 9.49 min).

5,6,7,8-Tetrahydroquinolin-8-ol ((±)-8)[37]

(±)-**7** (12.4 g, 65 mmol, 1.0 eq) was dissolved in CH_3OH (450 mL) and NaOH (1 M, 250 mL) was added. The mixture was stirred for 1 h at room temperature. MeOH was removed *in vacuo* and the solution was neutralized with HCl (1 M). The layers were separated and the aqueous layer was extracted with EtOAc (9 x 200 mL). The combined organic layers were dried (Na_2SO_4) and the solvent was removed *in vacuo*. The crude product was purified by flash column chromatography (d = 8 cm, h = 15 cm, V = 60 mL, CH_2Cl_2/CH_3OH = 98:2 → 95:5, R_f = 0.32 (CH_2Cl_2/MeOH = 98:2)). Yellow oil, yield 9.5 g (98 %). Chemical formula: $C_9H_{11}NO$ (149.2 g/mol). ^{1}H NMR (400 MHz, $CDCl_3$): δ [ppm] = 1.77 - 1.89 (m, 1 H, 6-*H*), 1.90 - 2.00 (m, 1 H, 7-*H*), 2.00 - 2.09 (m, 1 H, 6-*H*), 2.17 - 2.30 (m, 1 H, 7-*H*), 2.78 - 2.95 (m, 2 H, 2 x 5-*H*), 4.96 (dd, *J* = 7.6/5.3 Hz, 1 H, 8-*H*), 5.53 (s (broad), 1 H, O*H*), 7.35 (dd, *J* = 7.7/4.9 Hz, 1 H, 3-*H*), 7.68 (d, *J* = 8.0 Hz, 1 H, 4-*H*), 8.44 (d, *J* = 4.8 Hz, 1 H, 2-*H*). ^{13}C NMR (101 MHz, $CDCl_3$): δ [ppm] = 19.2 (1 C, C-6), 28.5 (1 C, C-5), 30.5 (1 C, C-7), 67.0 (1 C, C-8), 123.4 (1 C, C-3), 134.5 (1 C, C-4a), 140.5 (1 C, C-4), 143.6 (1 C, C-2), 157.0 (1 C, C-8a). IR (neat): $\tilde{\nu}$ [cm^{-1}] = 3213 (O-H), 2936 (C-H, aliphatic). Exact mass (APCI): *m/z* = 150.0923, calculated 150.0913 for $C_9H_{12}NO$ $[M+H]^+$. Purity (HPLC method 1): 79.9 % (t_R = 4.23 min).

(*S*)-5,6,7,8-Tetrahydroquinolin-8-ol[38] ((*S*)-8) and (*R*)-5,6,7,8-tetrahydroquinolin-8-yl acetate ((*R*)-7)[38]

Freshly distilled isopropenyl acetate (100 mL, 1.0 mol, 11 eq) was added to a suspension of (±)-**8** (13.8 g, 92 mmol, 1.0 eq) and Amano Lipase PS-IM (6.9 g, 50 wt%) in TBME (1.6 L). The mixture was stirred at room temperature for 140 h. After filtration and removal of the solvent *in vacuo*, the residue was purified by flash column chromatography (d = 8 cm, h = 22 cm, V = 60 mL, C_6H_{12}/EtOAc = 25:75 → 20:80, then 100 % EtOAc, then EtOAc/CH_3OH = 91:9, R_f ((*R*)-**7**) = 0.45 (C_6H_{12}/EtOAc = 25:75), R_f ((*S*)-**8**) = 0.23 (C_6H_{12}/EtOAc = 25:75)). Acetate (*R*)-**7**: Yellow oil, yield 7.9 g (45 %, 96.2 %ee (method 3)). Crude alcohol (*S*)-**8** was purified again by flash column chromatography (d = 6 cm, h = 18 cm, V = 40 mL, C_6H_{12}/EtOAc = 25:75). Alcohol (*S*)-

8: Yellow oil, yield 4.4 g (32 %, 99.7 %ee (HPLC method 2)). Specific rotation: $[\alpha]_D^{20}$ ((*S*)-**8**) = -15.9 (c = 2.9 mg/mL, CH_3OH).

(*R*)-5,6,7,8-Tetrahydroquinolin-8-ol ((*R*)-8)[38] and (*R*)-5,6,7,8-tetrahydroquinolin-8-yl acetate[38] ((*R*)-7)

Acetate (*R*)-**7** (7.8 g, 41 mmol, 1.0 eq, 96.2 %ee) was hydrolyzed with NaOH (1 M, 150 mL) and CH_3OH (280 mL) according to the procedure for (±)-**8**. The crude product was purified by flash column chromatography (d = 8 cm, h = 20 cm, V = 60 mL, C_6H_{12}/EtOAc = 25:75). Alcohol (*R*)-**8**: Yellow oil, yield 4.75 g (78 %, 94.0 %ee (HPLC method 2)).

Freshly distilled isopropenyl acetate (35 mL, 321 mol, 10 eq) was added to a suspension of the obtained alcohol (4.75 g, 32 mmol, 1.0 eq, 94.0 %ee) and Amano Lipase PS-IM (2.38 g, 50 wt%) in TBME (550 mL). The mixture was stirred at room temperature for 52 h. After filtration and removal of the solvent *in vacuo*, the residue was purified by flash column chromatography (d = 8 cm, h = 17 cm, V = 60 mL, CH_2Cl_2/CH_3OH = 98:2). Crude acetate (*R*)-**7** was purified again by flash column chromatography (d = 8 cm, h = 18 cm, V = 60 mL, C_6H_{12}/EtOAc = 25:75). Acetate (*R*)-**7**: Yellow oil, yield 5.34 g (87 %, 99.5 %ee (HPLC method 3).

Acetate (*R*)-**7** (5.30 g, 28 mmol, 1.0 eq, 99.5 %ee) was hydrolyzed with NaOH (1 M, 110 mL) and CH_3OH (190 mL) and the product was purified according to the procedure for (±)-**8**. Alcohol (*R*)-**8**: Yellow oil, yield 4.06 g (97 %, 99.1 %ee (HPLC method 2). Specific rotation: $[\alpha]_D^{20}$ ((*R*)-**8**) = +13.3 (c = 3.1 mg/mL, CH_3OH).

8-[(*tert*-Butyldimethylsilyl)oxy]-5,6,7,8-tetrahydroquinoline[39] ((±)-9)

Alcohol (±)-**8** (2.7 g, 18.1 mmol, 1.0 eq) was dissolved in DMF (50 mL) and imidazole (1.7 g, 25.0 mmol, 1.4 eq) was added. After cooling the solution to 0 °C, *tert*-butyldimethylsilyl chloride (4.4 g, 29.2 mmol, 1.6 eq) dissolved in DMF (25 mL), was added. The mixture was stirred for 16 h at room temperature. The solvent was removed *in vacuo* and the residue was dissolved in EtOAc. Water was added, the layers were separated, and the aqueous layer was extracted with EtOAc (4 x 50 mL). The combined organic layers were dried (Na_2SO_4) and the solvent was removed *in vacuo*. The crude product was purified with flash column chromatography (d = 5 cm, h = 15 cm, V = 60 mL, C_6H_{12}/EtOAc = 95:5, R_f = 0.35 (Cy/EtOAc = 95:5)). Colorless oil, yield 3.0 g (63 %). Chemical formula: $C_{15}H_{25}NOSi$ (263.4 g/mol). ^1H NMR (400 MHz, $CDCl_3$):

δ [ppm] = 0.12 (s, 3 H, C*H*3), 0.24 (s, 3 H, C*H*3), 0.88 (s, 9 H, C(C*H*3)3), 1.72 - 1.81 (m, 1 H, 6-*H*), 1.86 (ddt, *J* = 13.5/11.9/3.4 Hz, 1 H, 7-*H*), 2.02-2.10 (m, 1 H, 7-*H*), 2.10 - 2.20 (m, 1 H, 6-*H*), 2.73 (ddd, *J* = 16.9/10.3/6.0 Hz, 1 H, 5-*H*), 2.88 (dt, *J* = 17.2/4.3 Hz, 1 H, 5-*H*), 4.97 (s (broad), 1 H, 8-*H*), 7.23 (dd, *J* = 7.4/5.0 Hz, 1 H, 3-*H*), 7.54 (d, *J* = 7.7 Hz, 1 H, 4-*H*), 8.47 (d, *J* = 4.8 Hz, 1 H, 2-*H*). ^{13}C NMR (101 MHz, $CDCl_3$)**:** δ [ppm] = -4.69 (1 C, *C*H3), -3.91 (1 C, CH3), 17.1 (1 C, C-6), 18.4 (1 C, *C*(CH3)3), 26.0 (3 C, C(*C*H3)3), 28.2 (1 C, C-5), 31.9 (1 C, C-7), 68.7 (1 C, C-8), 123.1 (1 C, C-3), 133.7 (1 C, C-4a), 139.2 (1 C, C-4), 145.1 (1 C, C-2), 156.3 (1 C, C-8a). IR (neat)**:** $\tilde{\nu}$ [cm^{-1}] = 2931 (C-H, aliphatic), 1084 (C-O, ether). Exact mass (APCI)**:** *m/z* = 264.1785, calculated 264.1783 for $C_{15}H_{26}NOSi$ [M+H]$^+$. Purity (HPLC method 1)**:** 99.4 % (t_R = 19.4 min).

(*R*)-8-[(*tert*-Butyldimethylsilyl)oxy]-5,6,7,8-tetrahydroquinoline ((*R*)-9)

Alcohol (*R*)-**8** (4.0 g, 26.8 mmol, 1.0 eq) was reacted with *tert*-butyldimethylsilyl chloride (6.5 g, 43.1 mmol, 1.6 eq) and imidazole (2.6 g, 38.2 mmol, 1.4 eq) in DMF (110 mL) as described for (±)-**9**. Colorless oil, yield 6.4 g (91 %). Specific rotation: $[\alpha]_D^{20}$ = +26.2 (c = 3.6 m/mL, CH_3OH).

(*S*)-8-[(*tert*-Butyldimethylsilyl)oxy]-5,6,7,8-tetrahydroquinoline ((*S*)-9)

Alcohol (*S*)-**8** (4.4 g, 29.5 mmol, 1.0 eq) was reacted with *tert*-butyldimethylsilyl chloride (7.1 g, 47.1 mmol, 1.6 eq) and imidazole (2.8 g, 41.1 mmol, 1.4 eq) in DMF (130 mL) as described for (±)-**9**. Colorless oil, yield 7.1 g (91 %). Specific rotation: $[\alpha]_D^{20}$ = -25.2 (c = 3.0 mg/mL, CH_3OH).

(4a*RS*,8*RS*,8a*SR*)-8-[(*tert*-Butyldimethylsilyl)oxy]decahydroquinoline ((±)-10)

Silyl ether (±)-**9** (5.2 g, 19.7 mmol, 1.0 eq) was dissolved in acetic acid (5 mL) and Rh/Al_2O_3 (5 %, 0.5 g, 0.24 mmol, 0.01 eq) was added. The mixture was stirred at room temperature under H_2 (5 bar) for 25 h. Afterwards, saturated K_2CO_3 solution was added until pH 8 and the mixture was extracted with EtOAc (8 x 30 mL). The organic layer was dried (Na_2SO_4) and filtered. After removal of the solvent *in vacuo*, the residue was purified by flash column chromatography (d = 8 cm, h = 14 cm, V = 60 mL, CH_2Cl_2/MeOH = 95:5 → 91:9 then CH_2Cl_2/MeOH/Et_3N = 90:9:1, R_f = 0.30 (CH_2Cl_2/CH_3OH = 83:17)). Yellow oil, yield 5.0 g (94 %). Chemical formula: $C_{15}H_{31}NOSi$ (269.5 g/mol). ^{1}H NMR (600 MHz, CD_3OD)**:** δ [ppm] = 0.08 (s, 3 H, C*H*3),

0.09 (s, 3 H, C*H*3), 0.91 (s, 9 H, C(C*H*3)3), 1.13 - 1.18 (m, 1 H, 5-*H*), 1.31 (qt, *J* = 13.8/3.7 Hz, 1 H, 6-*H*ax), 1.40 - 1.46 (m, 1 H, 3-*H*), 1.50 - 1.55 (m, 1 H, 7-*Heq*), 1.55 - 1.61 (m, 2 H, 4-*H*ax, 4-*H*eq), 1.61 - 1.67 (m, 2 H, 4a-*H*, 7-*Hax*), 1.67 - 1.72 (m, 1 H, 3-*H*), 1.72 - 1.77 (m, 2 H, 5-*H*, 6-*H*eq), 2.59 (ddd, *J* = 12.5/11.2/2.8 Hz, 1 H, 2-*H*ax), 2.85 (t, *J* = 3.5 Hz, 1 H, 8a-*H*), 3.09 - 3.13 (m, 1 H, 2-*H*eq), 3.72 (ddd, *J* = 11.4/5.0/4.0 Hz, 1 H, 8-*H*). A signal for the N*H* proton is not seen in the spectrum. ^{13}C NMR (151 MHz, CD_3OD)**:** δ [ppm] = -4.70 (1 C, *C*H3), -4.47 (1 C, *C*H3), 18.9 (1 C, *C*(CH3)3), 22.4 (1 C, C-3), 24.9 (1 C, C-6), 25.1 (1 C, C-5), 26.3 (3 C, C(*C*H3)3), 30.3 (1 C, C-7), 31.0 (1 C, C-4), 36.6 (1 C, C-4a), 48.3 (1 C, C-2), 62.4 (1 C, C-8a), 74.6 (1 C, C-8). NOE difference spectrum (8a-*H*, δ = 2.85 ppm, CD_3OD): 1.64 (4a-*H*, 7-*H*ax) 2.59 ppm (2-*H*ax), 3.27 (8-*H*ax). IR (neat)**:** $\tilde{\nu}$ [cm-1] = 2928 (C-H, aliphatic), 1088 (C-O, ether). Exact mass (APCI)**:** *m/z* = 270.2237, calculated 270.2248 for $C_{15}H_{32}NOSi$ $[M+H]^+$.

(4a*R*,8*R*,8a*S*)-8-[(*tert*-Butyldimethylsilyl)oxy]decahydroquinoline (10)

Silyl ether (*R*)-**9** (6.3 g, 23.9 mmol, 1.0 eq) was hydrogenated (5 bar) using Rh/Al_2O_3 (5 %, 0.6 g, 0.29 mmol, 0.01 eq) in acetic acid (13 mL) as described for (±)-**10**.
Grey oil, yield 7.7 g (120 %, residual catalyst and SiO_2 in product). Specific rotation: $[\alpha]_D^{20}$ = +10.1 (c = 3.4 mg/mL, CH_3OH).

(4a*S*,8*S*,8a*R*)-8-[(*tert*-Butyldimethylsilyl)oxy]decahydroquinoline (*ent*-10)

Silyl ether (*S*)-**9** (7.0 g, 26.6 mmol, 1.0 eq) was hydrogenated (5 bar) using Rh/Al_2O_3 (5 %, 0.7 g, 0.34 mmol, 0.01 eq) in acetic acid (13 mL) as described for (±)-**10**.
Yellow oil, yield 6.9 g (96 %). Specific rotation: $[\alpha]_D^{20}$ = -12.9 (c = 3.1 mg/mL, CH_3OH).

(4a*RS*,8*RS*,8a*SR*)-Decahydroquinolin-8-ol[40] ((±)-11)

A solution of TBAF·3H_2O (2.2 g, 6.97 mmol, 3.0 eq) in THF (4 mL) was added to a solution of silyl ether (±)-**10** (626 mg, 2.32 mmol, 1.0 eq) in THF (10 mL). The mixture was stirred for 18 h at room temperature. After addition of H_2O (15 mL) and brine (5 mL), the mixture was extracted with EtOAc (6 x 20 mL). The combined organic layers were dried (Na_2SO_4), filtered and the solvent was removed *in vacuo*. The residue was purified by flash column chromatography (d = 3 cm, h = 3 cm, V = 30 mL, CH_2Cl_2/CH_3OH = 91:9 and then $CH_2Cl_2/CH_3OH/Et_3N$ = 90:9:1, R_f = 0.27 ($CH_2Cl_2/CH_3OH/Et_3N$ = 90:9:1)). Yellow oil, yield 319 mg (88 %). Chemical formula: $C_9H_{17}NO$ (155.2 g/mol). ^{1}H NMR (400 MHz, $CDCl_3$)**:** δ [ppm] = 1.23 - 1.32 (m, 2 H, 5-

H, 6-*H*), 1.41 - 1.56 (m, 1 H, 3-*H*), 1.56 - 1.78 (m, 6 H, 4a-*H*, 4-H_{ax}, 4-H_{eq}, 5-*H*, 7-H_{ax}, 7-$H_{eq,}$), 1.78 - 1.92 (m, 2 H, 3-*H*, 6-*H*), 2.70 (td, *J* = 12.7/3.1 Hz, 1 H, 2-H_{ax}), 3.20 (t, *J* = 3.5 Hz, 1 H, 8a-*H*), 3.30 - 3.40 (m, 1 H, 2-H_{eq}), 3.74 (ddd, *J* = 11.3/5.6/4.3 Hz, 1 H, 8-*H*), 4.36 (s (broad), 1 H, O*H*). A signal for the N*H* proton is not seen in the spectrum. ^{13}C-NMR (101 MHz, $CDCl_3$)**:** δ [ppm] = 20.4 (1 C, C-3), 23.6 (1 C, C-6), 24.0 (1 C, C-5), 28.7 (1 C, C-7), 29.0 (1 C, C-4), 34.2 (1 C, C-4a), 46.3 (1 C, C-2), 60.0 (1 C, C-8a), 70.7 (1 C, C-8). IR (neat)**:** $\tilde{\nu}$ [cm^{-1}] = 3156 (OH/NH), 2924 (C-H, aliphatic). Exact mass (APCI)**:** *m/z* = 156.1397, calculated 156.1383 for $C_9H_{18}NO$ $[M+H]^+$.

(4a*R*,8*R*,8a*S*)-Decahydroquinolin-8-ol (11)

Silyl ether **10** (7.7 g, 28.6 mmol, 1.0 eq) was reacted with TBAF·3H_2O (31.4 g, 99.5 mmol, 3.5 eq) in THF (155 mL) as described for (±)-**11**. Yellow oil, yield 4.1 g (92 %). Specific rotation: $[\alpha]_D^{20}$ = +16.2 (c = 3.3 mg/mL, CH_3OH).

(4a*S*,8*S*,8a*R*)-Decahydroquinolin-8-ol (*ent*-11)

Silyl ether *ent*-**10** (6.9 g, 25.6 mmol, 1.0 eq) was reacted with TBAF·3H_2O (16.2 g, 51.3 mmol, 2.0 eq) in THF (140 mL) as described for (±)-**11**. Yellow oil, yield 3.4 g (86 %). Specific rotation: $[\alpha]_D^{20}$ = -20.4 (c = 3.1 mg/mL, CH_3OH).

(6a*RS*,9a*RS*,9b*SR*)-2,2-Dioxo-5,6,6a,7,8,9,9a,9b-octahydro-4*H*-[1,2,3]oxathiazolo[5,4,3-ij]quinoline ((±)-12)

Under a N_2 atmosphere, (±)-**11** (319 mg, 2.05 mmol, 1.0 eq) was dissolved in dry CH_2Cl_2 (5 mL) and Et_3N (0.78 mL, 5.63 mmol, 2.7 eq) was added. At 0 °C SO_2Cl_2 (0.32 mL, 4.49 mmol, 2.2 eq) dissolved in dry CH_2Cl_2 (5 mL) was added dropwise. The mixture was then allowed to reach room temperature and was stirred for 20 h. After addition of H_2O (10 mL), the mixture was extracted with EtOAc (4 x 10 mL), the combined organic layers were dried (Na_2SO_4), filtered and the solvent was removed *in vacuo*. The residue was purified by flash column chromatography (d = 3 cm, h = 15 cm, V = 30 mL, C_6H_{12}/EtOAc = 98:2 → 95:5 → 90:10 → 80:20, R_f = 0.24 (C_6H_{12}/EtOAc = 80:20)). Yellow oil, yield 150 mg (34 %). Chemical formula: $C_9H_{15}NO_3S$ (217.3 g/mol). ^{1}H NMR (400 MHz, $CDCl_3$)**:** δ [ppm] = 1.15 - 1.25 (m, 1 H, 8-*H*), 1.33 - 1.44 (m, 1 H, 7-*H*), 1.48 - 1.60 (m, 1 H, 6-*H*), 1.61 - 1.74 (m, 3 H, 5-*H*, 6-*H*, 7-*H*), 1.75 - 1.92 (m, 3 H, 5-*H*, 8-*H*, 9-H_{ax}), 1.92 - 2.01 (m, 1 H, 6a-*H*), 2.09 - 2.16 (m, 1 H, 9-H_{eq}), 2.69 (ddd, *J* = 12.3/10.9/3.0 Hz, 1 H, 4-H_{ax}), 3.50 - 3.58 (m, 2 H, 4-H_{eq}, 9b-*H*), 4.74 (ddd,

J = 10.5/6.6/5.3 Hz, 1 H, 9a-*H*). ^{13}C-NMR (101 MHz, $CDCl_3$)**:** δ [ppm] = 19.4 (1 C, C-5), 22.0 (1 C, C-8), 23.7 (1 C, C-7), 27.3 (1 C, C-6), 27.9 (1 C, C-9), 34.2 (1 C, C-6a), 44.1 (1 C, C-4), 58.7 (1 C, C-9b), 82.9 (1 C, C-9a). IR (neat)**:** $\tilde{\nu}$ [cm^{-1}] = 2936 (C-H, aliphatic), 1331 and 1180 (O=S=O). Exact mass (APCI)**:** *m/z* = 218.0833, calculated 218.0845 for $C_9H_{16}NO_3S$ [M+H]$^+$.

(6a*R*,9a*R*,9b*S*)-2,2-Dioxo-5,6,6a,7,8,9,9a,9b-octahydro-4*H*-[1,2,3]oxathiazolo[5,4,3-ij]quinoline (12)

β-Amino alcohol **11** (443 mg, 2.85 mmol, 1.0 eq) was reacted with SO_2Cl_2 (0.44 mL, 6.18 mmol, 2.2 eq) and Et_3N (1.09 mL, 7.86 mmol, 2.8 eq) in dry CH_2Cl_2 (13 mL) as described for (±)-**12**. Yellow oil, yield 188 mg (30 %). Specific rotation: $[\boldsymbol{\alpha}]_{\boldsymbol{D}}^{\mathbf{20}}$ = +36.8 (c = 3.3 mg/mL, CH_3OH).

(6a*S*,9a*S*,9b*R*)-2,2-Dioxo-5,6,6a,7,8,9,9a,9b-octahydro-4*H*-[1,2,3]oxathiazolo[5,4,3-ij]quinoline (*ent*-12)

β-Amino alcohol *ent*-**11** (480 mg, 3.09 mmol, 1.0 eq) was reacted with SO_2Cl_2 (0.48 mL, 6.74 mmol, 2.2 eq) and Et_3N (1.18 mL, 8.51 mmol, 2.8 eq) in dry CH_2Cl_2 (15 mL) as described for (±)-**12**. Yellow oil, yield 232 mg (35 %). Specific rotation: $[\boldsymbol{\alpha}]_{\boldsymbol{D}}^{\mathbf{20}}$ = -33.1 (c = 3.1 mg/mL, CH_3OH).

(4a*RS*,8*SR*,8a*SR*)-8-(Pyrrolidin-1-yl)-decahydroquinoline[41] ((±)-13)

^{1}H NMR (600 MHz, 26 °C, CD_3OD)**:** δ [ppm] = 1.42 - 1.57 (m, 5 H, 3-*H* + 4 H out of 4-*H*, 5-*H*, 6-H and 7-*H*), 1.60 - 1.72 (m, 4 H, 4a-H + 3 H out of 4-*H*, 5-*H*, 6-H and 7-*H*), 1.73 - 1.81 (m, 6 H, 3-*H*, $N(CH_2CH_2)_2$ + 1 H out of 4-*H*, 5-*H*, 6-H and 7-*H*), 2.08 (s (broad), 1 H, 8a-*H*), 2.60 - 2.69 (m, 4 H, $N(CH_2CH_2)_2$), 2.72 - 2.78 (m, 1 H, 2-*H*), 2.91 - 2.97 (m, 1 H, 2-*H*), 2.98 - 3.01 (m, 1 H, 8-*H*). ^{13}C NMR (151 MHz, 26 °C, CD_3OD)**:** δ [ppm] = 21.3 (1 C, C-3), 24.5 (2 C, $N(CH_2CH_2)_2$), 58.0 (1 C, C-8). ^{13}C NMR (151 MHz, 50 °C, CD_3OD)**:** δ [ppm] = 21.3 (1 C, C-3), 24.4 (1 C, C-4, C-5, C-6 or C-7), 24.5 (1 C, C-4, C-5, C-6 or C-7), 24.6 (2 C, $N(CH_2CH_2)_2$), 27.6 (1 C, C-4, C-5, C-6 or C-7), 29.5 (1 C, C-4, C-5, C-6 or C-7), 34.0 (1 C, C-8a), 43.4 (1 C, C-2), 50.2 (2 C, $N(CH_2CH_2)_2$), 58.1 (1 C, C-8). A signal for the C-4a carbon is not seen in the spectrum. IR (neat)**:** $\tilde{\nu}$ [cm^{-1}] = 3356 (N-H), 2924 (C-H, aliphatic), 1115 (N-C). Exact mass (APCI)**:** *m/z* = 209.2013, calculated 209.2012 for $C_{13}H_{25}N_2$ [M+H]$^+$.

2-(3,4-Dichlorophenyl)-1-[(4a*RS*,8*SR*,8a*SR*)-8-(pyrrolidin-1-yl)-3,4,4a,5,6,7,8,8a-octahydroquinolin-1(2*H*)-yl]ethan-1-one[41] ((±)-4)

^{1}H NMR (600 MHz, CD_3OD): δ [ppm] = 1.28 - 1.43 (m, 3 H, 3-*H,* 3-*H**, 5-*H*, 5-*H**, 7-*H,* 7-*H**), 1.48 - 1.64 (m, 4 H, 4-H_{ax}, 4-H_{eq}, 4-H_{ax}*, 4-H_{eq}*, 6-H_{ax}, 6-H_{eq}, 6-H_{ax}*, 6-H_{eq}*), 1.65 - 1.76 (m, 5.2 H, 3-*H*, 3-*H**, 4a-*H**, $N(CH_2CH_2)_2$, $N(CH_2CH_2)_2$*), 1.81 - 1.93 (m, 1.8 H, 4a-*H*, 5-*H*, 5-*H**), 1.93 - 1.99 (m, 0.8 H, 7-*H*), 2.02 - 2.07 (m, 0.2 H, 7-*H**), 2.55 - 2.66 (m, 1.6 H, $N(CH_2CH_2)_2$), 2.66 - 2.70 (m, 0.8 H, $N(CH_2CH_2)_2$*), 2.70 - 2.75 (m, 0.2 H, 2-H_{ax}*), 2.73 - 2.85 (m, 1.6 H, $N(CH_2CH_2)_2$), 3.08 (td, *J* = 13.7/2.8 Hz, 0.8 H, 2-H_{ax}), 3.18 (td, *J* = 11.4/3.9 Hz, 0.2 H, 8-*H**), 3.65 - 3.72 (m, 0.8 H, 2-H_{eq}), 3.71 (d, *J* = 15.5 Hz, 0.2 H, O=C-CH_2-aryl*), 3.72 (d, *J* = 15.8 Hz, 0.8 H, O=C-CH_2-aryl), 3.82 - 3.86 (m, 0.2 H, 8a-*H**), 3.87 (d, *J* = 15.7 Hz, 0.8 H, O=C-CH_2-aryl), 3.90 - 3.96 (m, 0.2 H, O=C-CH_2-aryl*), 4.39 - 4.44 (m, 0.2 H, 2-H_{eq}*), 4.68 (dd, *J* = 11.6/4.2 Hz, 0.8 H, 8a-*H*), 7.20 (dd, *J* = 8.2/2.1 Hz, 0.2 H, 6-H_{arom}*), 7.25 (dd, *J* = 8.3/2.1 Hz, 0.8 H, 6-H_{arom}), 7.44 (d, *J* = 8.3 Hz, 0.8 H, 5-H_{arom}), 7.44 (d, *J* = 1.9 Hz, 0.2 H, 2-H_{arom}*) 7.46 (d, *J* = 8.1 Hz, 0.2 H, 5-H_{arom}*), 7.47 (d, *J* = 1.9 Hz, 0.8 H, 2-H_{arom}). The signal for 8-H of the major rotamer is overlapping with the signal of CD_3OD at 3.31 ppm. The ratio of the rotamers is 80 : 20. ^{13}C NMR (151 MHz, CD_3OD): δ [ppm] = 21.4, 21.5* (C-6), 24.6*, 24.8 (2 C, $N(CH_2CH_2)_2$), 25.1, 28.6* (C-7), 25.7, 26.0* (C-5), 26.7*, 27.6 (C-3), 32.0*, 32.3 (C-4), 37.9, 38.9* (C-4a), 38.6*, 43.0 (C-2), 40.2*, 40.9 (O=C-CH_2-aryl), 48.7, 50.9* (2 C, $N(CH_2CH_2)_2$), 54.6, 56.3* (C-8), 55.6, 62.3* (C-8a), 130.0, 130.2* (C-6_{arom}), 131.49, 131.51* (C-5_{arom}), 131.6*, 132.0 (C-2_{arom}), 132.3 (C-4_{arom}), 133.2*, 133.3 (C-3_{arom}), 137.8, 138.2* (C-1_{arom}), 171.0, 172.1* (C=O). A signal for C-4_{arom}* of the minor rotamer is not seen in the spectrum. Signals of the minor rotamer are marked with *. IR (neat): $\tilde{\nu}$ [cm^{-1}] = 2924 (C-$H_{aliphatic}$), 1628 (C=O), 1130 (C-N). Exact mass (APCI): *m/z* = 395.1647, calculated 395.1651 for $C_{21}H_{29}{}^{35}Cl_2N_2O$ $[M+H]^+$. Purity (HPLC method 1): 97.5 % (t_R = 19.00 min).

2-(3,4-Dichlorophenyl)-1-[(4a*R*,8*S*,8a*S*)-8-(pyrrolidin-1-yl)-3,4,4a,5,6,7,8,8a-octahydroquinolin-1(2*H*)-yl]ethan-1-one (4)

^{1}H NMR (600 MHz, CD_3OD): δ [ppm] = 1.27 - 1.44 (m, 3 H, 3-*H,* 3-*H**, 5-*H*, 5-*H**, 7-*H,* 7-*H**), 1.47 - 1.65 (m, 4 H, , 4-H_{ax}, 4-H_{eq}, 4-H_{ax}*, 4-H_{eq}*, 6-H_{ax}, 6-H_{eq}, 6-H_{ax}*, 6-H_{eq}*), 1.64 - 1.79 (m, 5.2 H, 3-*H*, 3-*H**, 4a-*H**, $N(CH_2CH_2)_2$, $N(CH_2CH_2)_2$*), 1.82 - 1.93 (m, 1.8 H, 4a-*H*, 5-*H*, 5-*H**), 1.94 - 1.99 (m, 0.8 H, 7-*H*), 2.03 - 2.07 (m, 0.2 H, 7-*H**), 2.54 - 2.67 (m, 1.6 H, $N(CH_2CH_2)_2$), 2.67 - 2.70 (m, 0.8 H, $N(CH_2CH_2)_2$*), 2.70 - 2.75 (m,

0.2 H, 2-H_{ax}*), 2.76 - 2.85 (m, 1.6 H, N(CH_2CH$_2$)$_2$), 3.08 (td, J = 13.7/2.8 Hz, 0.8 H, 2-H_{ax}), 3.15 - 3.20 (m, 0.2 H, 8-H*), 3.67 - 3.73 (m, 0.8 H, 2-H_{eq}), 3.70 (d, J = 15.5 Hz, 0.2 H, O=C-CH_2-aryl*), 3.72 (d, J = 15.7 Hz, 0.8 H, O=C-CH_2-aryl), 3.82 - 3.89 (m, 0.2 H, 8a-H*), 3.86 (d, J = 15.6 Hz, 0.8 H, O=C-CH_2-aryl), 3.93 (d, J = 15.5 Hz, 0.2 H, O=C-CH_2-aryl*), 4.39 - 4.44 (m, 0.2 H, 2-H_{eq}*), 4.68 (dd, J = 11.6/4.2 Hz, 0.8 H, 8a-H), 7.20 (dd, J = 8.2/2.1 Hz, 0.2 H, 6-H_{arom}*), 7.25 (dd, J = 8.2/2.0 Hz, 0.8 H, 6-H_{arom}), 7.44 (d, J = 8.2 Hz, 0.8 H, 5-H_{arom}), 7.44 (d, J = 2.0 Hz, 0.2 H, 2-H_{arom}*), 7.46 (d, J = 8.2 Hz, 0.2 H, 5-H_{arom}*), 7.47 (d, J = 2.1 Hz, 0.8 H, 2-H_{arom}). The signal for 8-H of the major rotamer is overlapping with the signal of CD_3OD at 3.31 ppm. The ratio of the rotamers is 80 : 20. ^{13}C NMR (151 MHz, CD_3OD): δ [ppm] = 21.4, 21.5* (C-6), 24.6*, 24.8 (2 C, N(CH$_2$$CH_2$)$_2$), 25.1, 28.6* (C-7), 25.7, 26.0* (C-5), 26.7*, 27.6 (C-3), 32.0*, 32.3 (C-4), 37.9, 38.9* (C-4a), 38.6*, 43.0 (C-2), 40.2*, 40.9 (O=C-CH$_2$-aryl), 48.7, 50.9* (2 C, N(CH$_2$CH$_2$)$_2$), 54.8, 56.3* (C-8), 55.6, 62.3* (C-8a), 130.0, 130.2* (C-6_{arom}), 131.49, 131.51* (C-5_{arom}), 131.6*, 132.0 (C-2_{arom}), 132.3 (C-4_{arom}), 133.2*, 133.3 (C-3_{arom}), 137.8, 138.2* (C-1_{arom}), 171.0, 172.1* (C=O). A signal for C-4_{arom}* of the minor rotamer is not seen in the spectrum. Signals of the minor rotamer are marked with *.

3. Chiral HPLC chromatograms of 7, 8 and KOR agonists 4

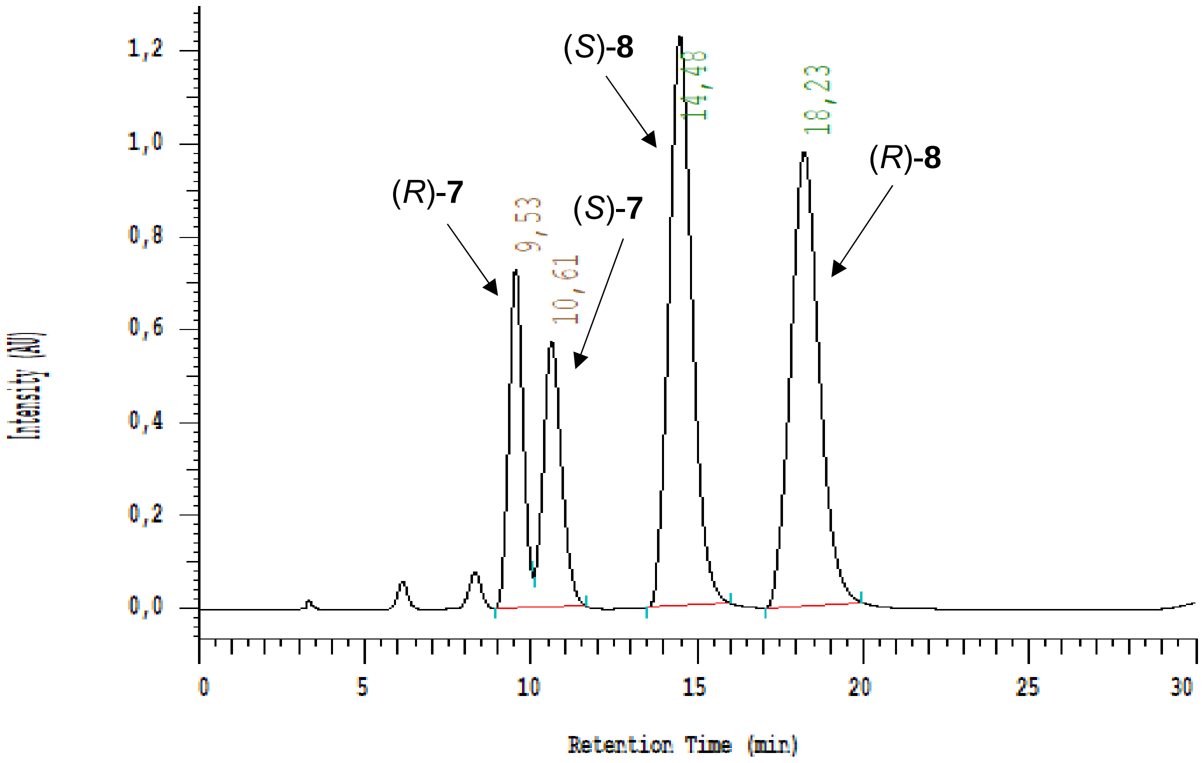

Figure S1: Chiral HPLC-UV chromatogram of racemic mixtures of acetate (±)-**7** and alcohol (±)-**8**. A 50:50 mixture of (±)-**7** and (±)-**8** was prepared and injected. DaicelChiralpak® AD-H, iso-hexane/methanol 95:5, 1 mL·min^{-1}, detection at λ 266 nm.

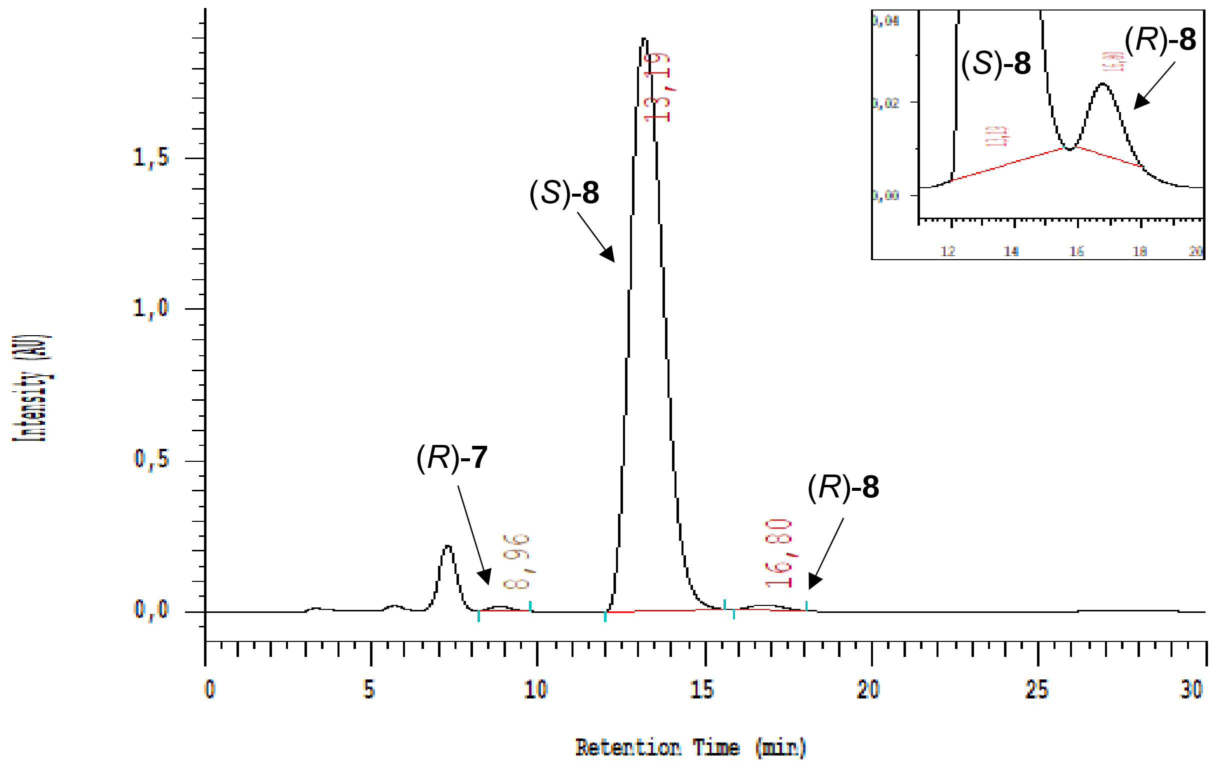

Figure S2: Chiral HPLC-UV chromatogram of alcohol (*S*)-**8** (98.4 %ee) after first lipase-catalyzed acetylation. An impurity of 0.509 % of acetate (*R*)-**7** (96.2 %ee) at 8.96 ppm was detected. DaicelChiralpak® AD-H, iso-hexane/methanol 95:5, 1 mL·min^{-1}, detection at λ 266 nm.

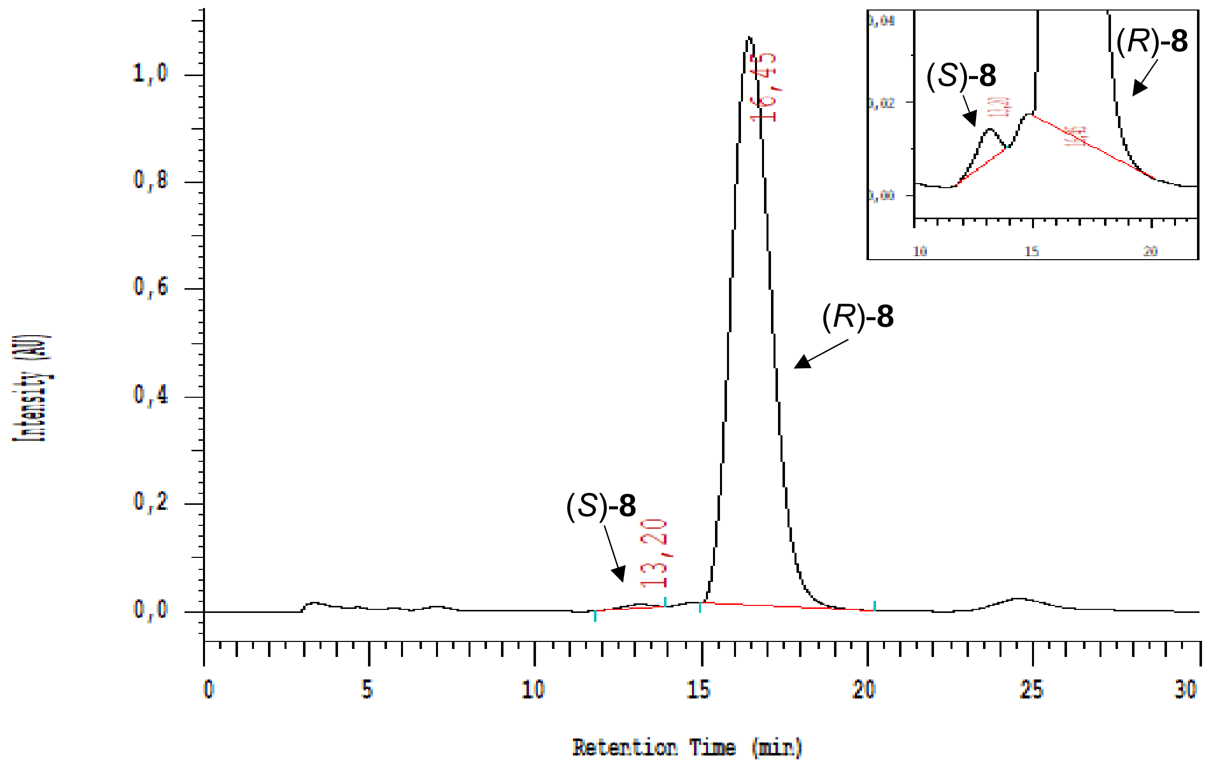

Figure S3: Chiral HPLC-UV chromatogram of alcohol (*R*)-**8** (99.1 %ee) after second lipase-catalyzed acetylation and ester hydrolysis. DaicelChiralpak® AD-H, iso-hexane/methanol 95:5, 1 mL·min^{-1}, detection at λ 266 nm.

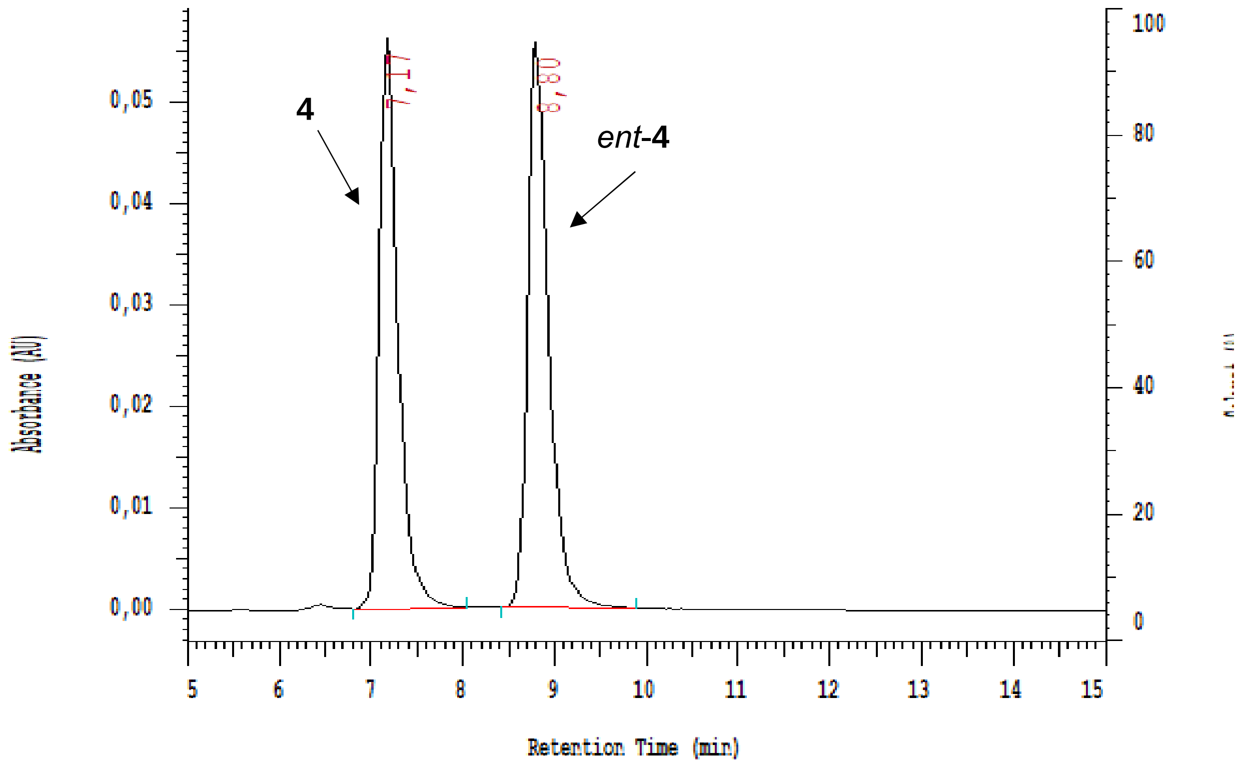

Figure S4: Chiral HPLC-UV chromatogram of racemic KOR-agonist (±)-**4**. DaicelChiralpak® IA, iso-hexane/isopropyl alcohol 90:10 + 0.1 % diethylamine, 1 mL·min^{-1}, detection at λ 275 nm.

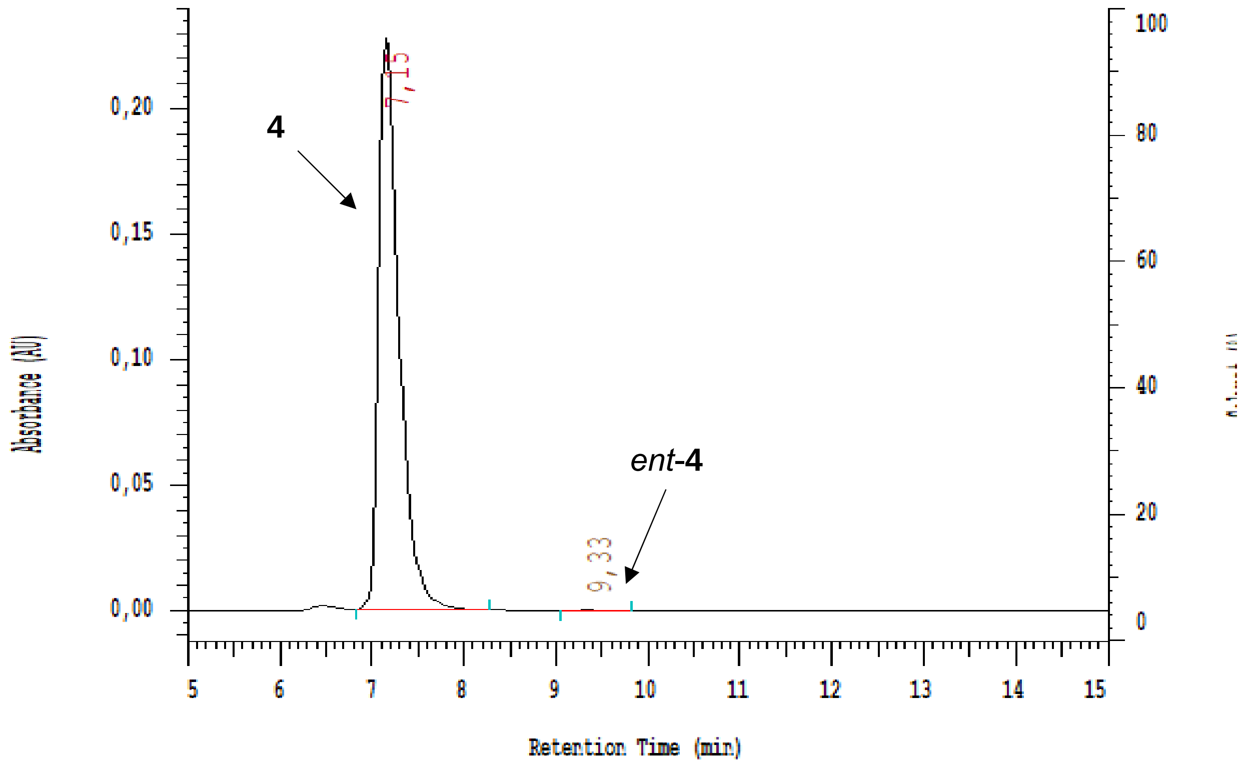

Figure S5: Chiral HPLC-UV chromatogram of KOR-agonist **4** (99.8 %ee). DaicelChiralpak® IA, iso-hexane/isopropyl alcohol 90:10 + 0.1 % diethylamine, 1 mL·min^{-1}, detection at λ 275 nm.

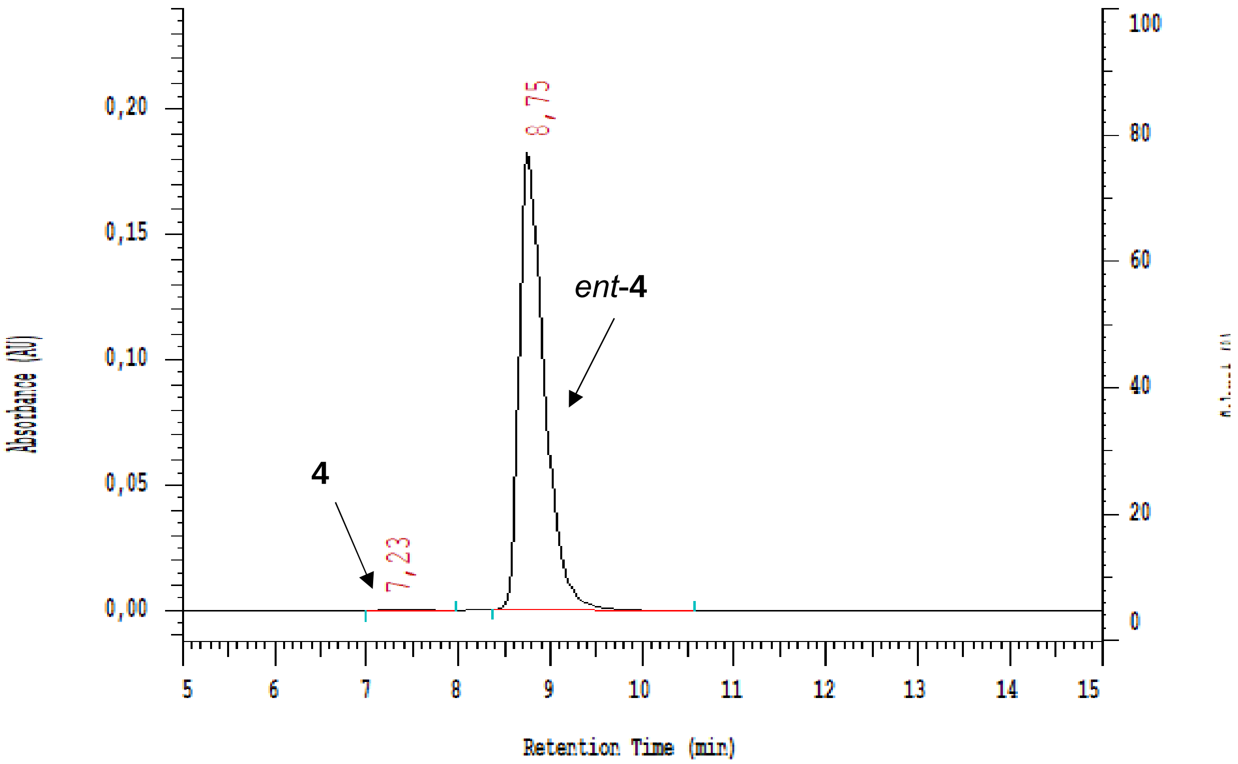

Figure S6: Chiral HPLC-UV chromatogram of KOR-agonist *ent*-**4** (99.0 %ee). DaicelChiralpak® IA, iso-hexane/isopropyl alcohol 90:10 + 0.1 % diethylamine, 1 mL·min^{-1}, detection at λ 275 nm.

4. Effect of (±)-4 on CD4$^+$ T cells

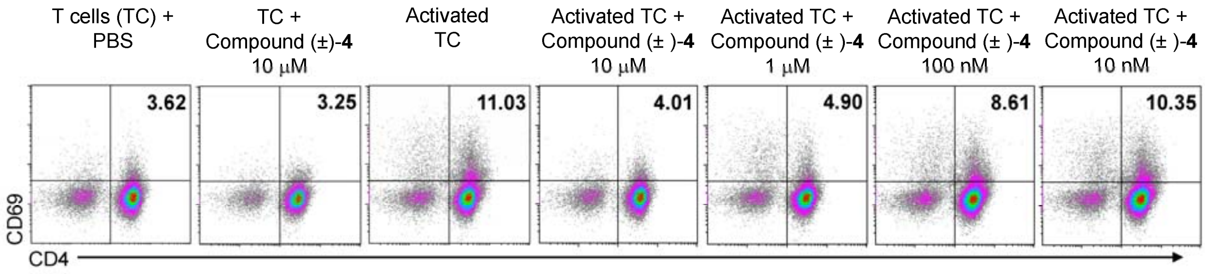

Figure S7: Compound (±)-**4** significantly reduced the expression of the early and transient activation marker CD69 in CD4$^+$ T cells when applied in an concentration of 10 µM or 1 µM. Total T cells were sorted from human peripheral blood mononuclear cells (PBMC) by magnetic beads, acitvated for 12 h with anti-CD3 and anti-CD28 as described (activated TC), and stimulated with different concentrations of compound (±)-**4** for additional 48 h. Control cells received an equal amount of PBS and exemplary dot-plots showing the percentages of CD69$^+$ cells within the CD4$^+$ T cell population are depicted. Cells are gated on CD3.

5. ^{1}H and ^{13}C NMR spectra

5,6,7,8-Tetrahydroquinolin-1-oxide (6)

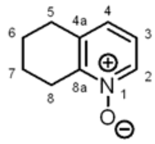

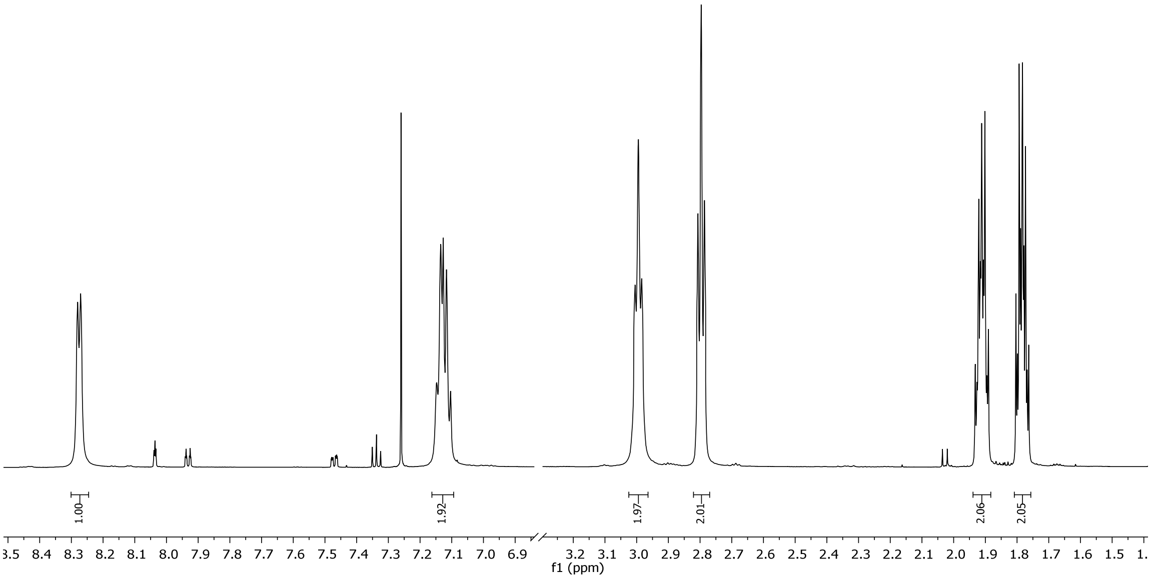

^{1}H NMR spectrum ($CDCl_3$) of **6**.

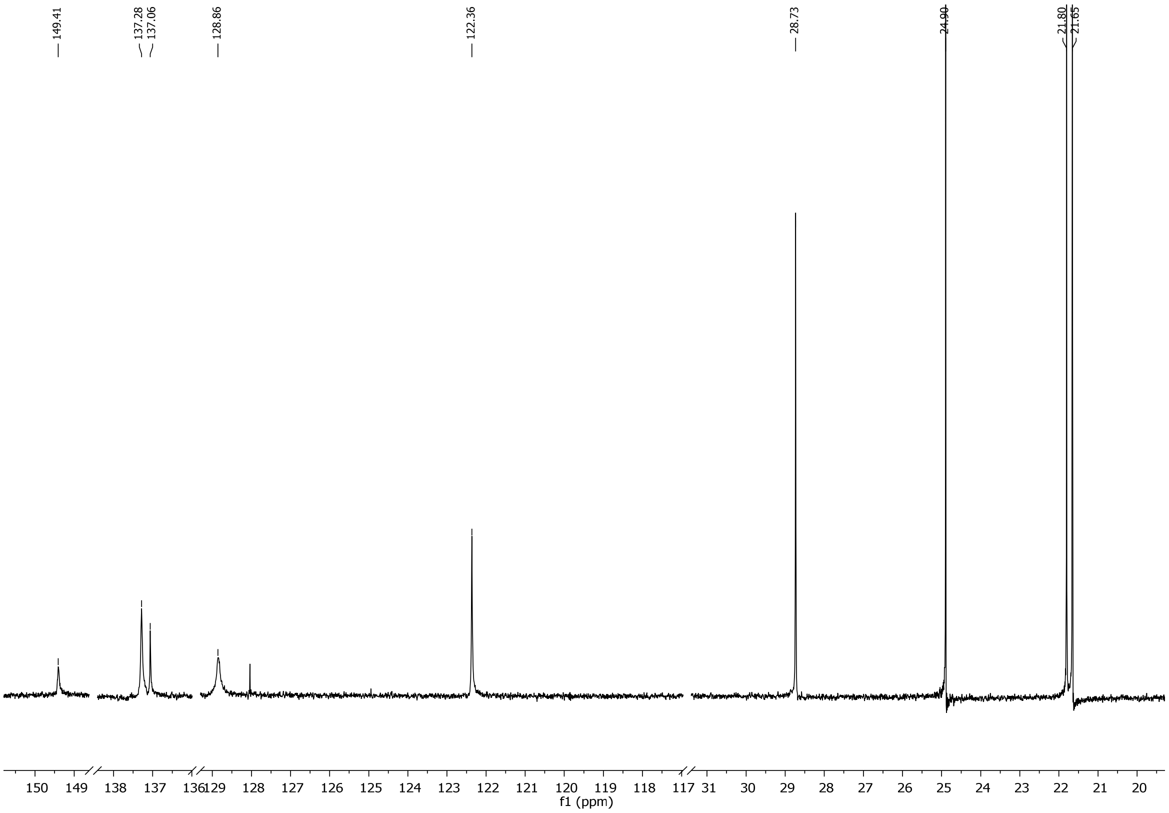

^{13}C NMR spectrum ($CDCl_3$) **6**.

5,6,7,8-Tetrahydroquinolin-8-yl acetate ((±)-7)

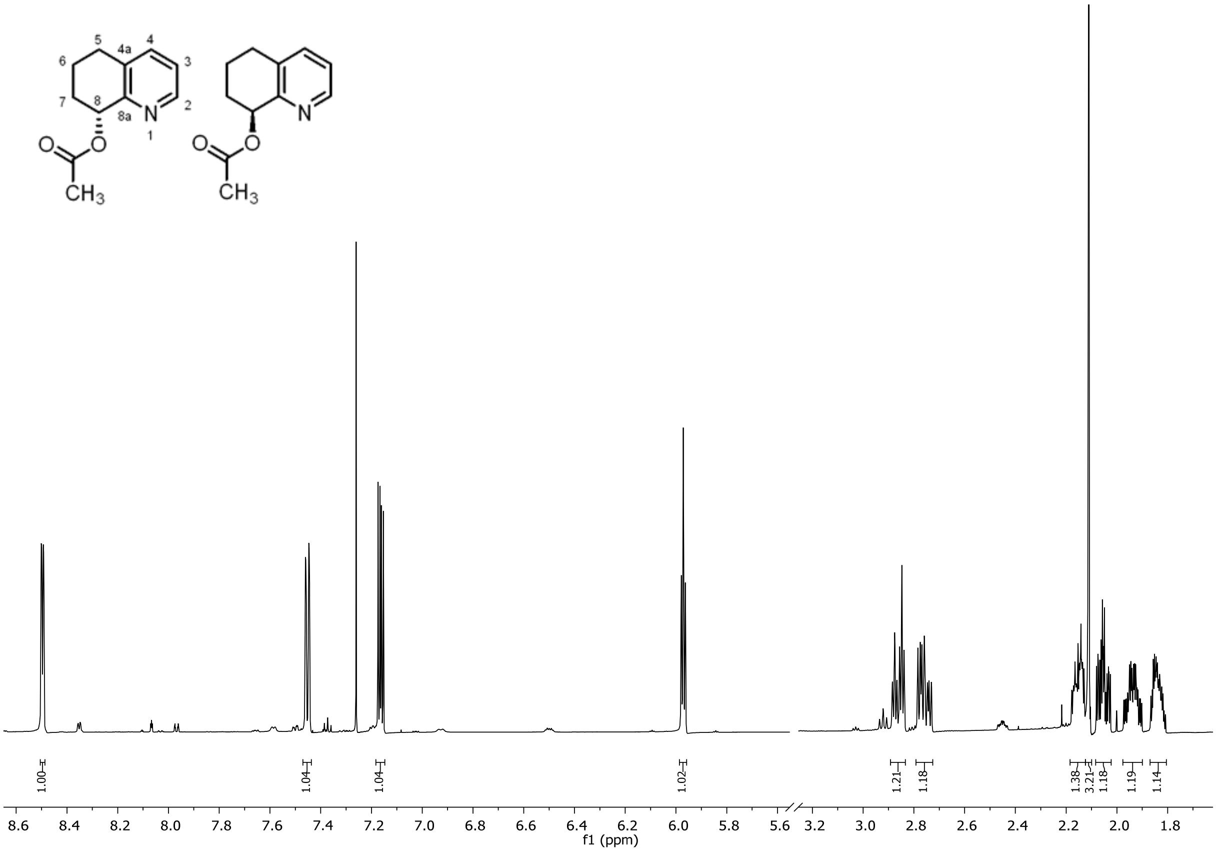

^{1}H NMR spectrum (CDCl$_3$) of (±)-**7**.

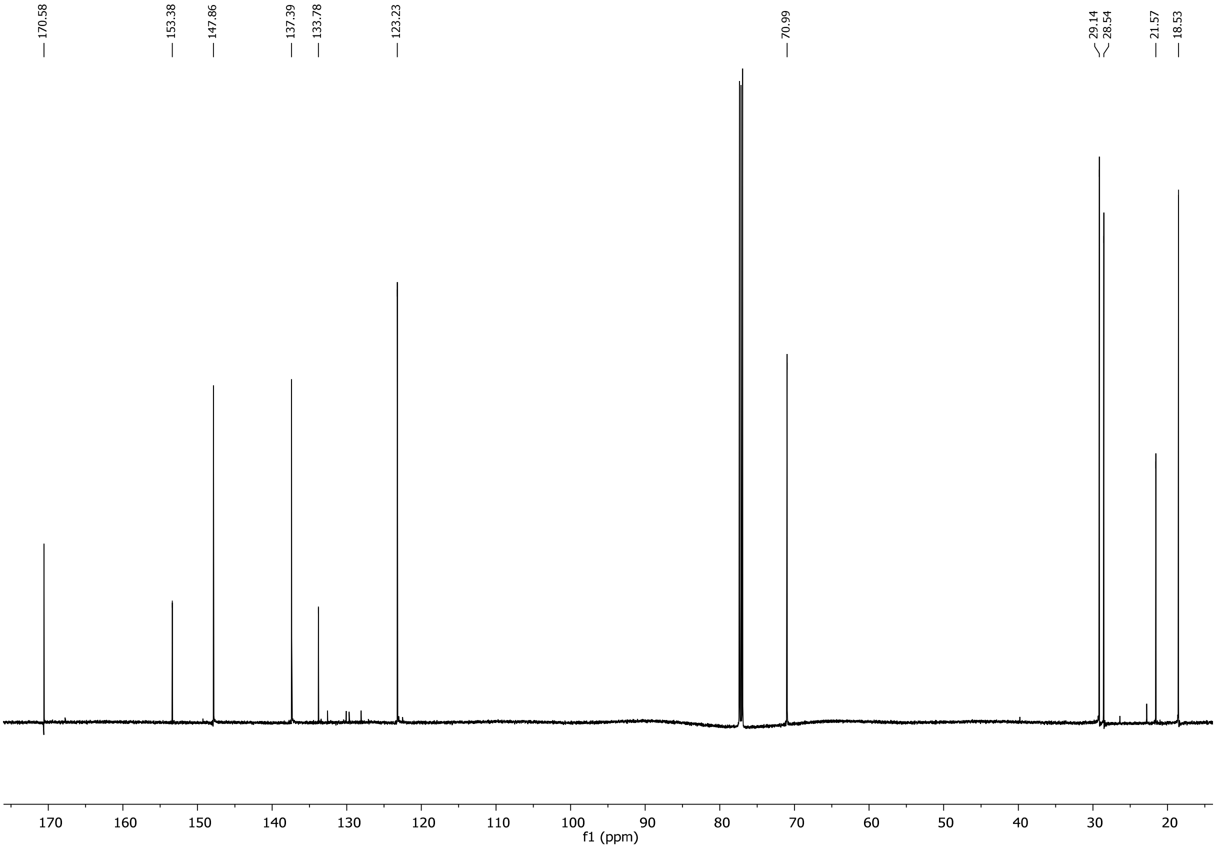

^{13}C NMR spectrum (CDCl$_3$) of (±)-**7**.

5,6,7,8-Tetrahydroquinolin-8-ol ((±)-8)

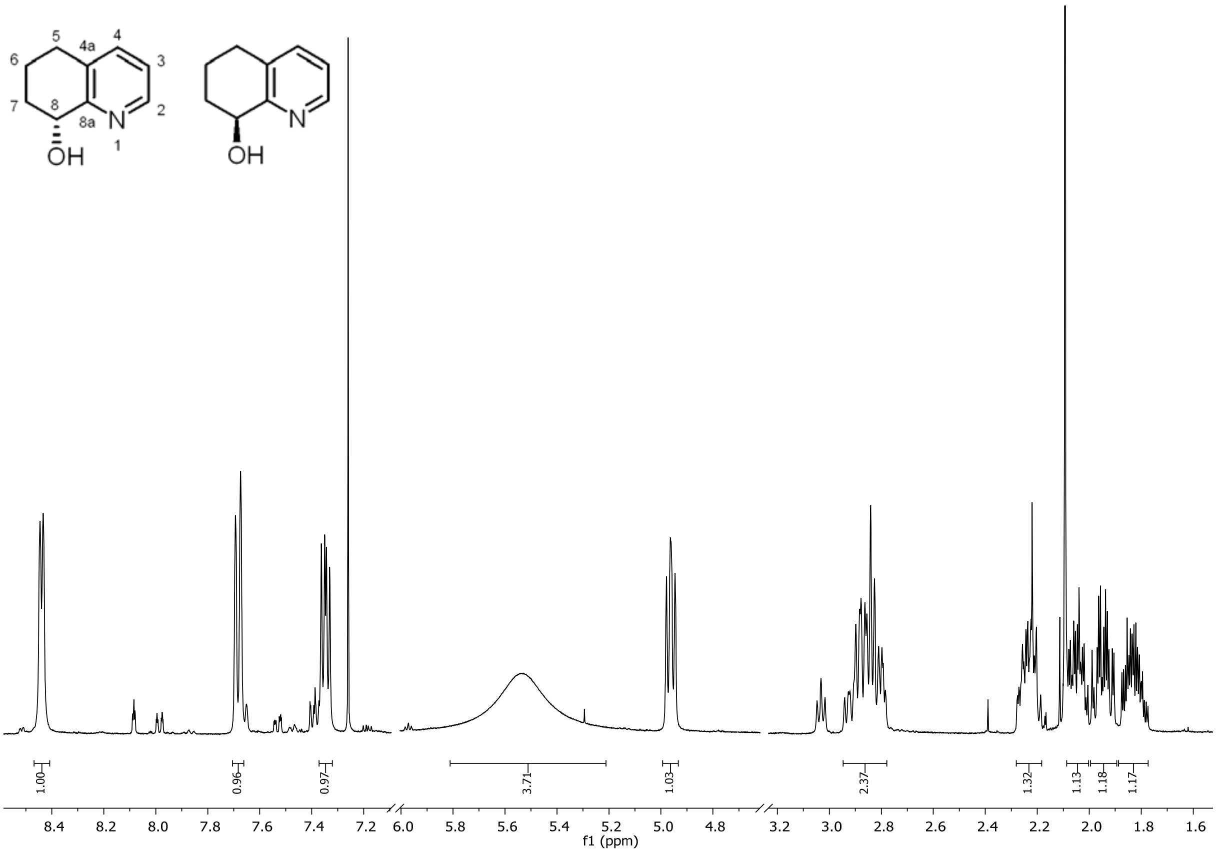

^{1}H NMR spectrum ($CDCl_3$) of (±)-**8**.

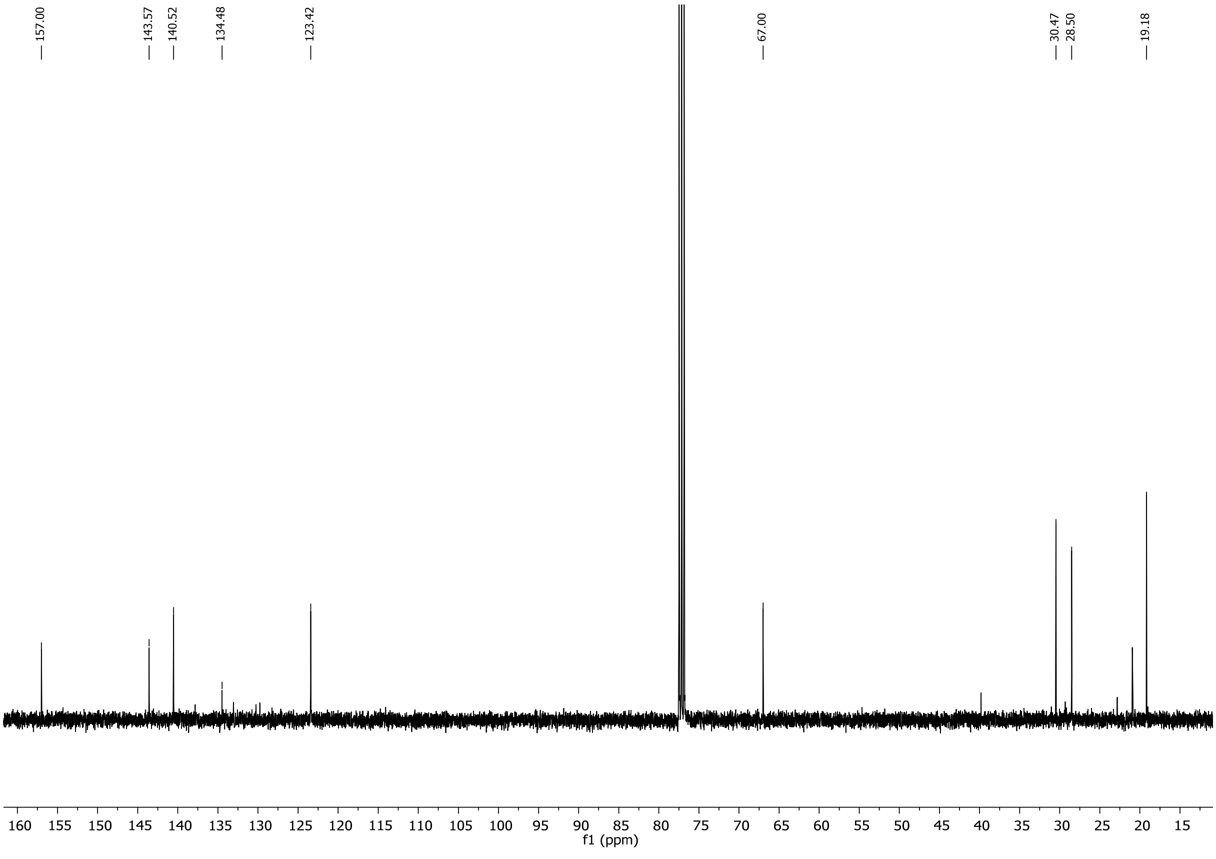

^{13}C NMR spectrum ($CDCl_3$) of (±)-**8**.

8-[(*tert*-Butyldimethylsilyl)oxy]-5,6,7,8-tetrahydroquinoline ((±)-9)

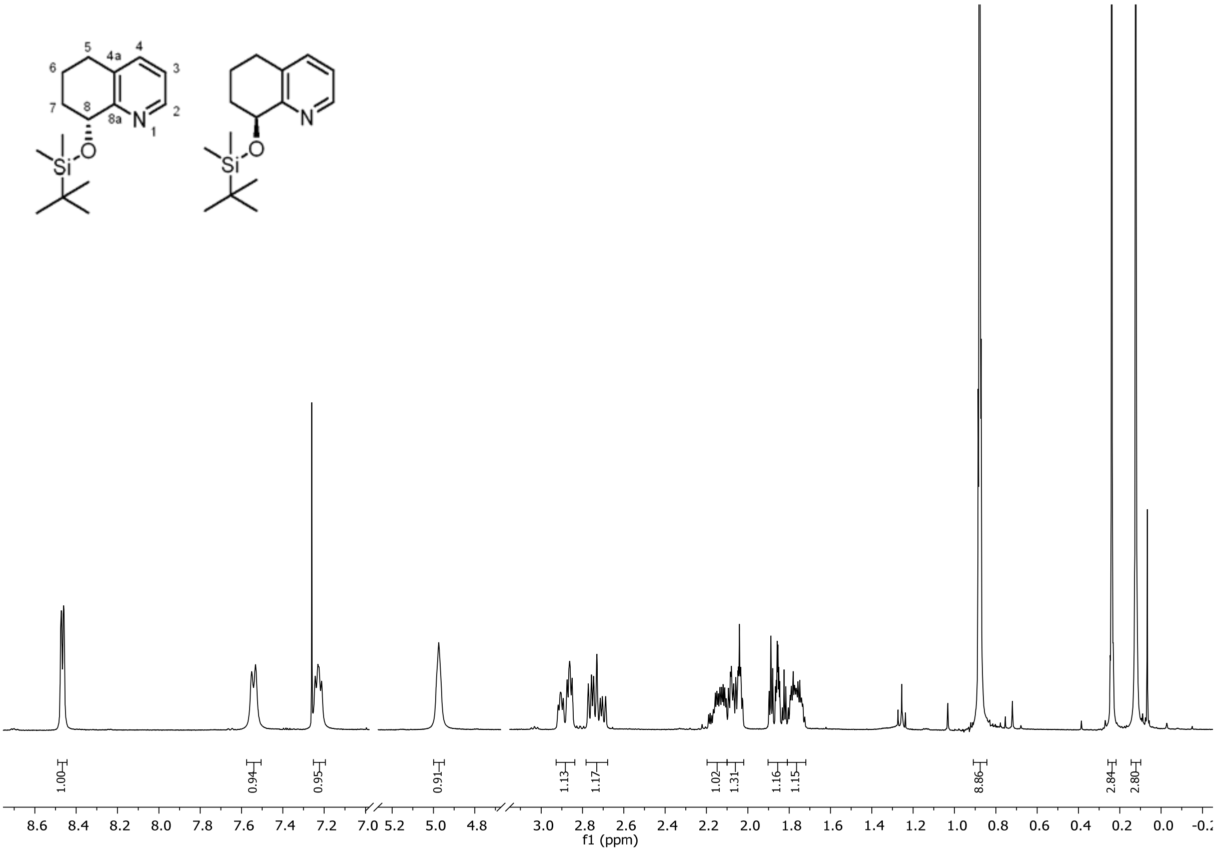

^{1}H NMR spectrum ($CDCl_3$) of (±)-**9**.

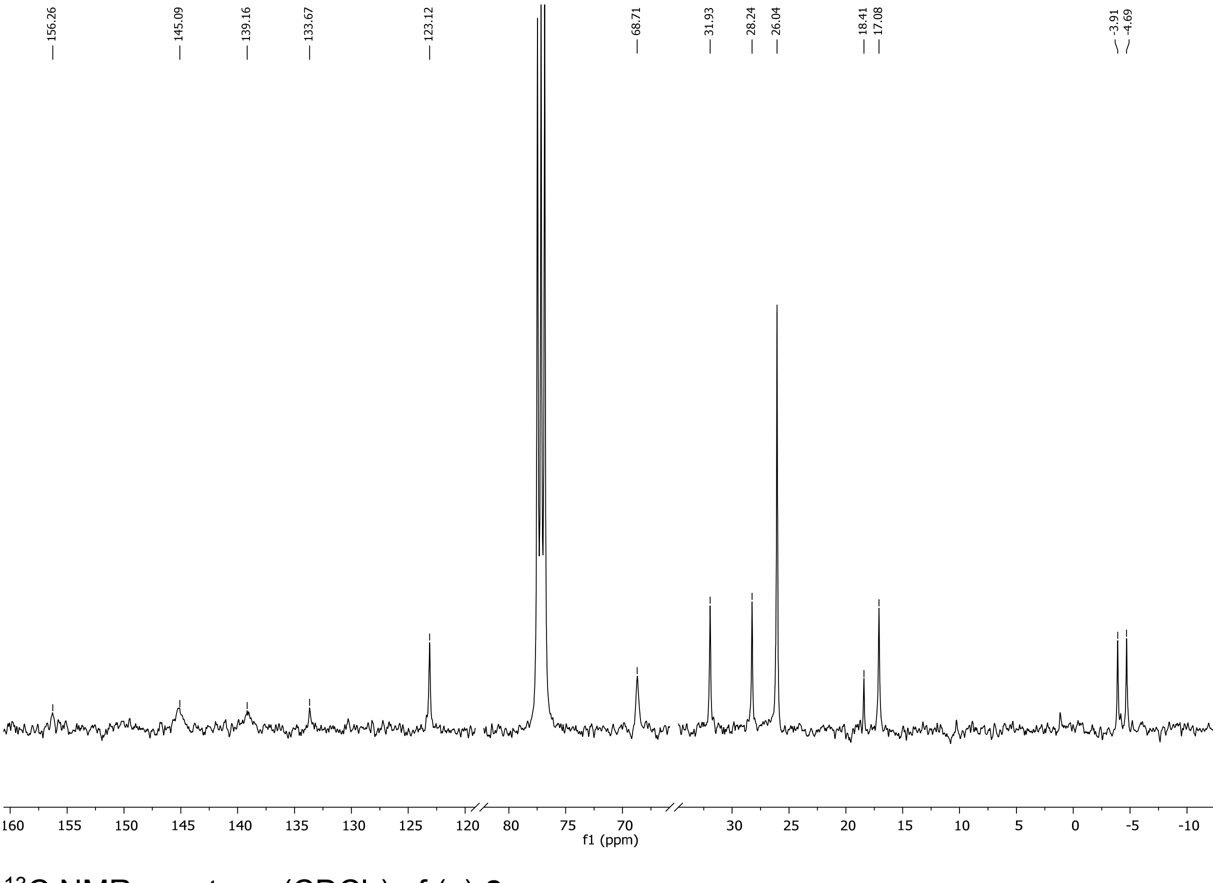

^{13}C NMR spectrum ($CDCl_3$) of (±)-**9**.

(4a*RS*,8*RS*,8a*SR*)-8-[(*tert*-Butyldimethylsilyl)oxy]decahydroquinoline ((±)-10)

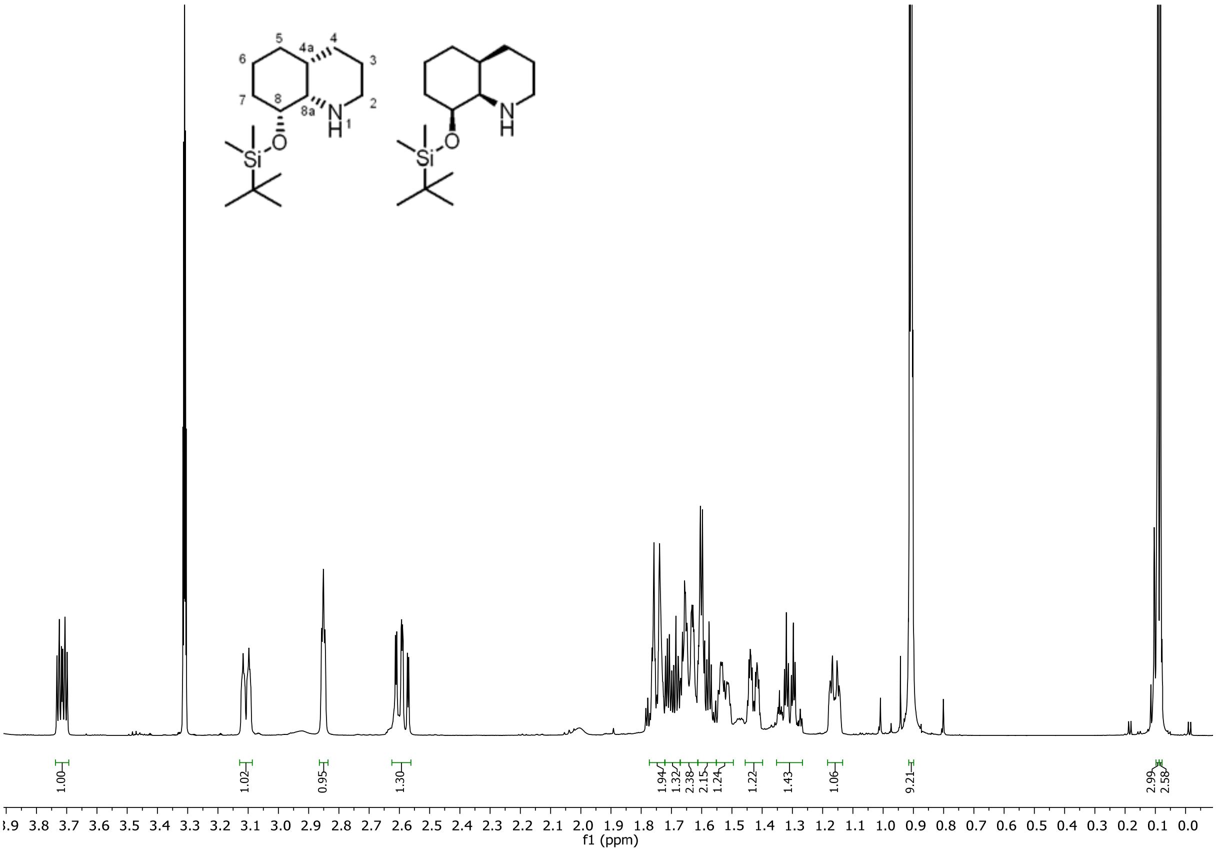

^{1}H NMR spectrum (CD_3OD) of (±)-**10**.

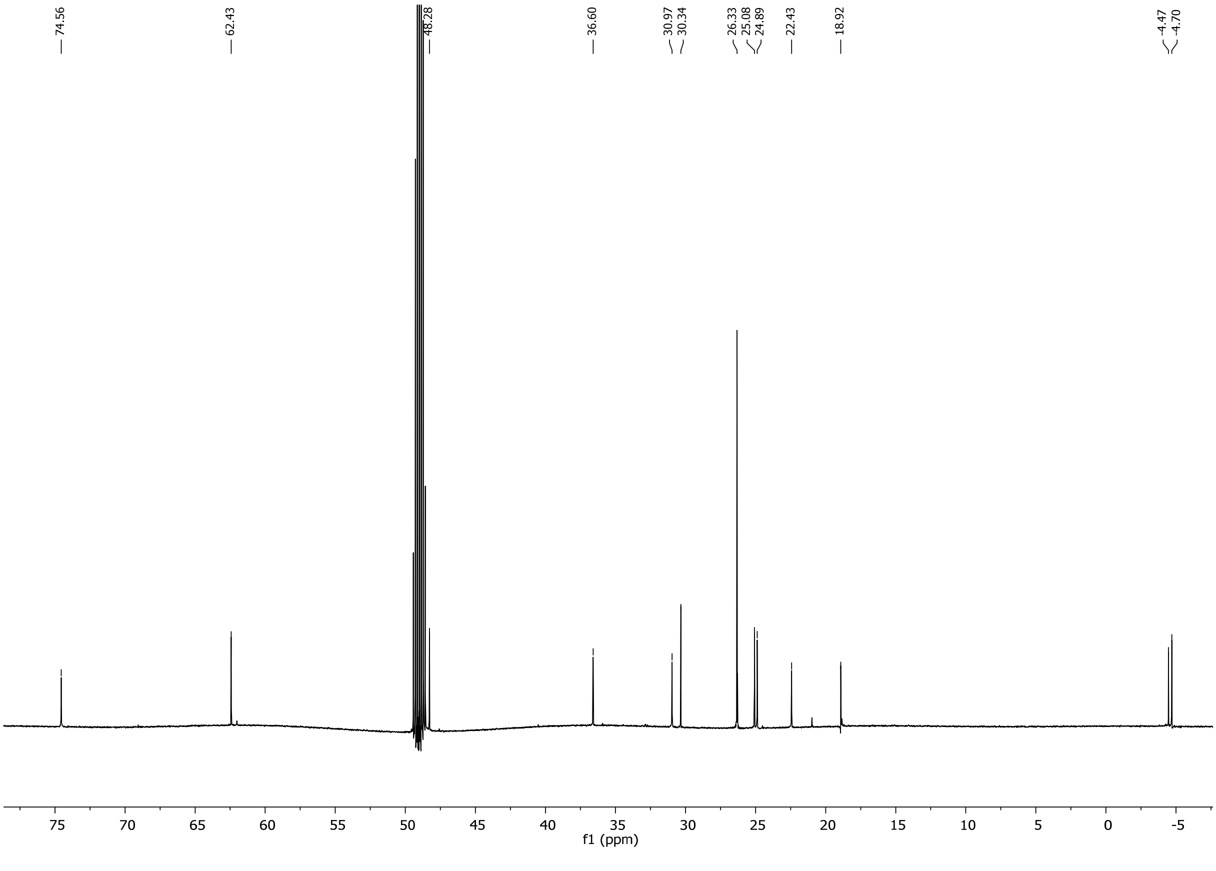

^{13}C NMR spectrum (CD_3OD) of (±)-**10**.

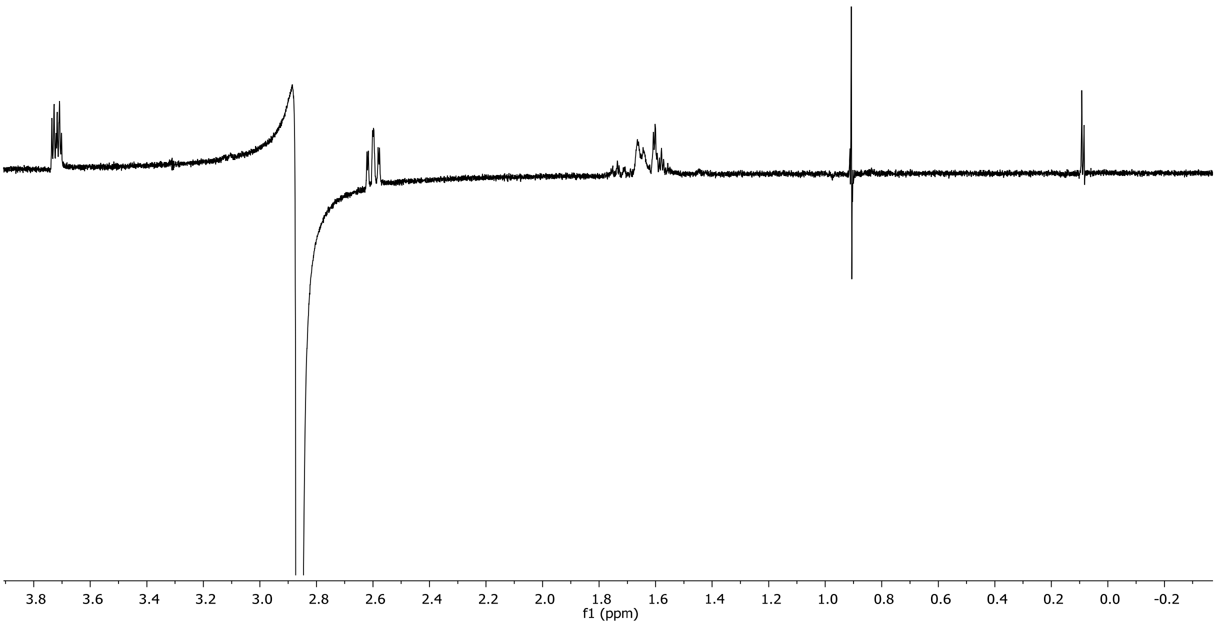

1D-NOESY NMR spectrum (CD_3OD) of (±)-**10** (8a-H).

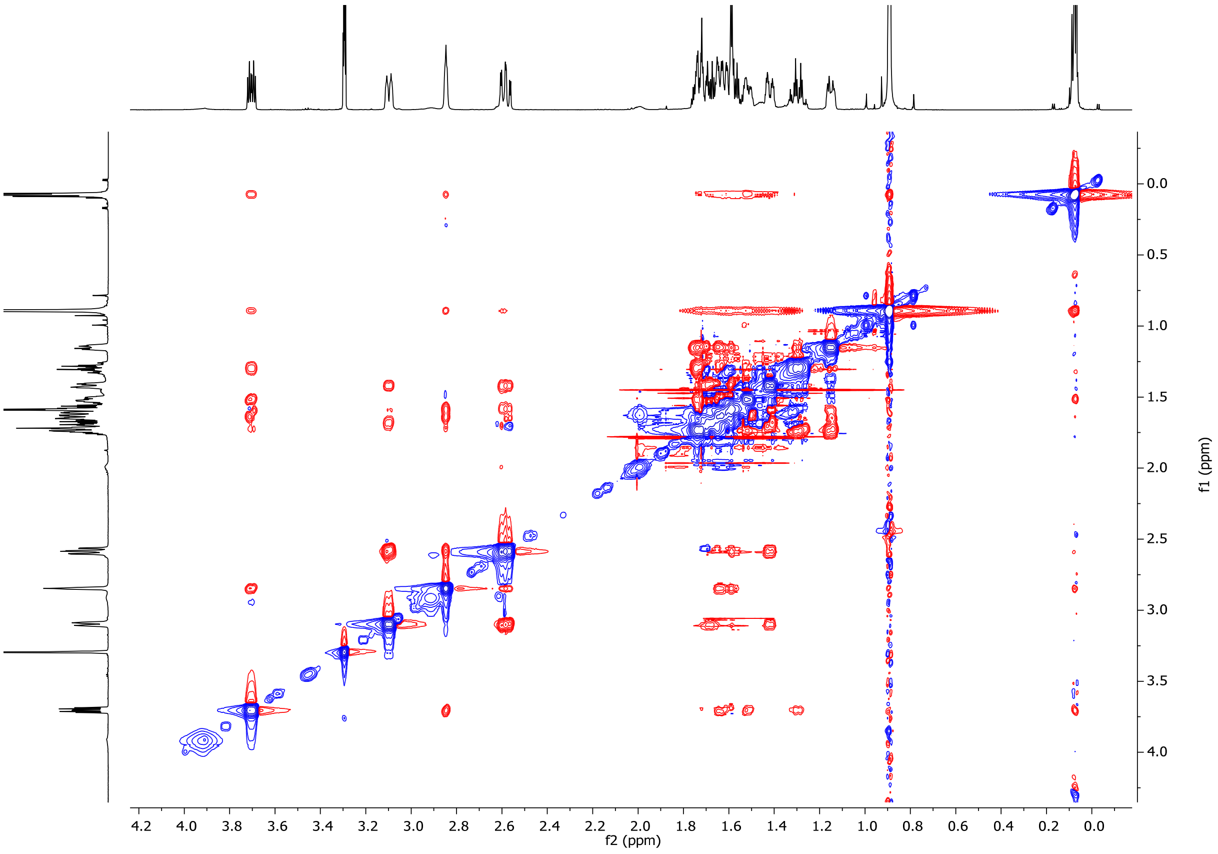

2D-NOESY NMR spectrum (CD_3OD) of (±)-**10**.

(4a*RS*,8*RS*,8a*SR*)-Decahydroquinolin-8-ol ((±)-11)

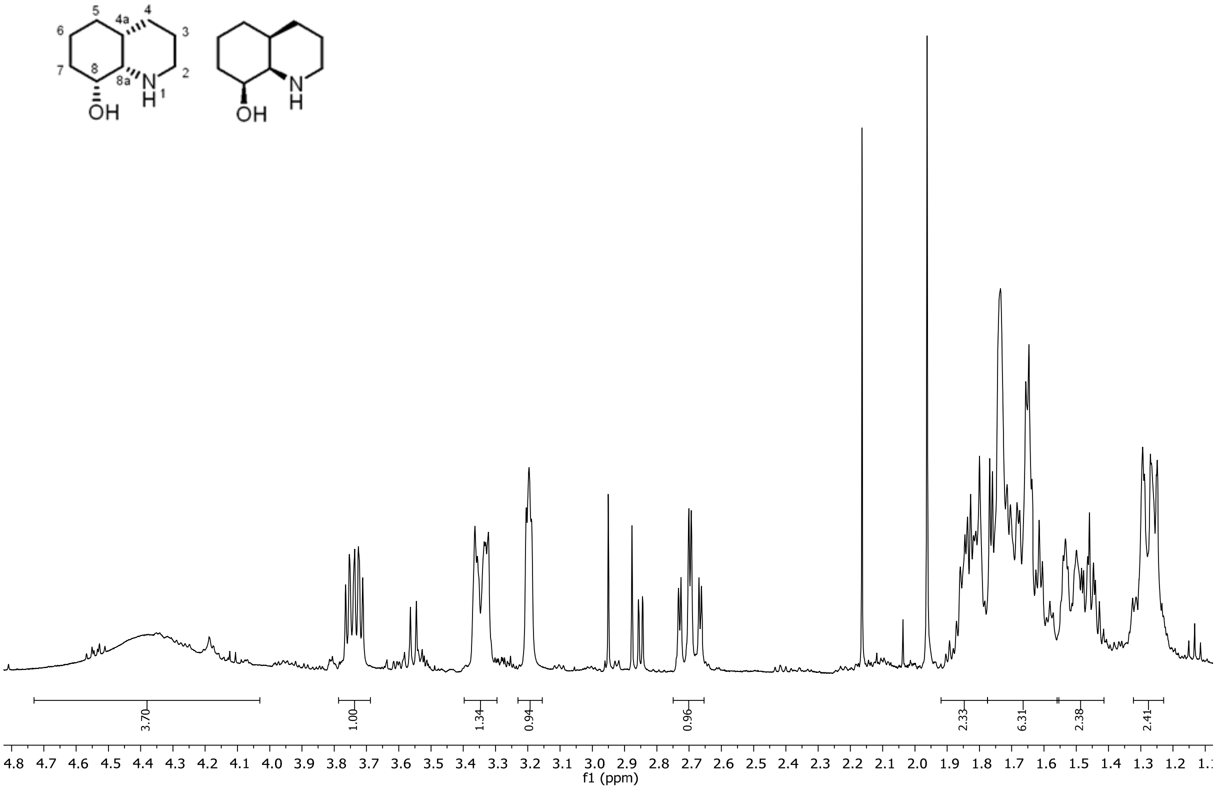

^{1}H NMR spectrum ($CDCl_3$) of (±)-**11**.

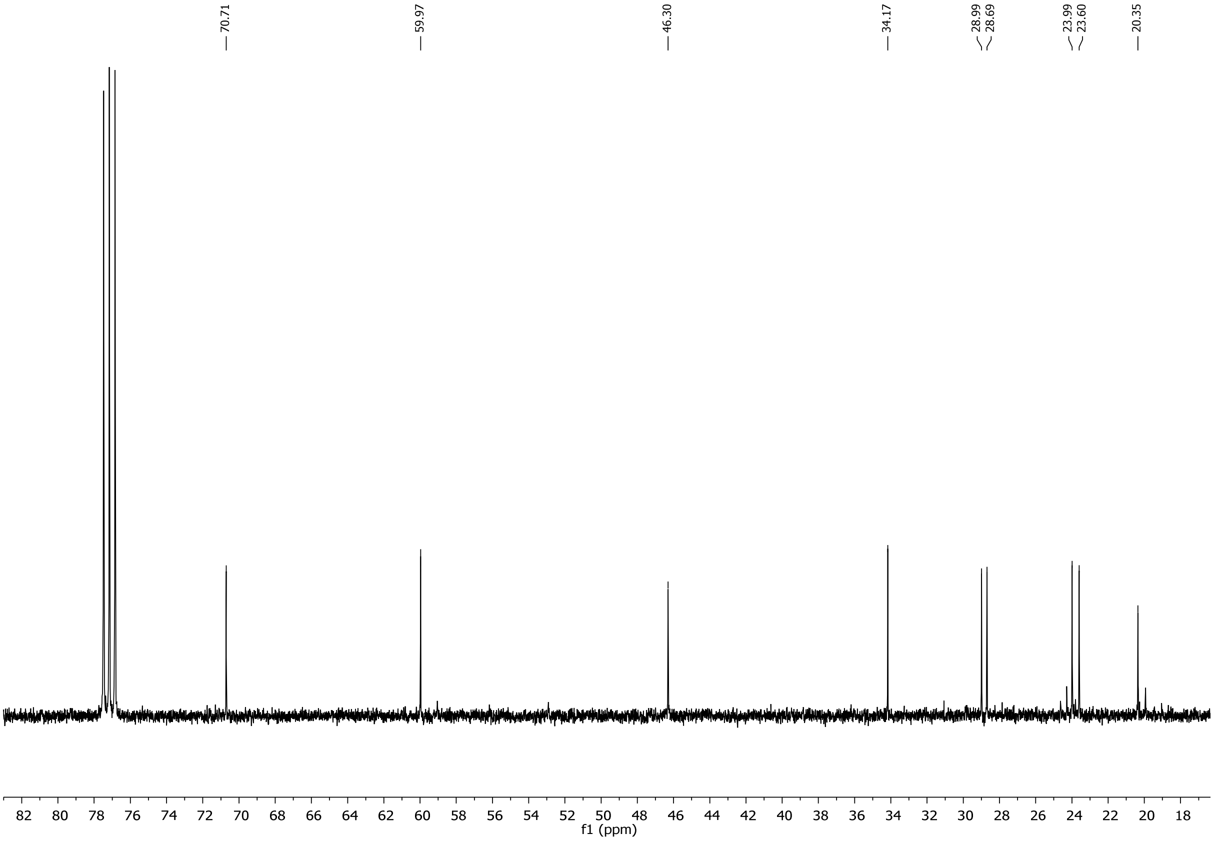

^{13}C NMR spectrum ($CDCl_3$) of (±)-**11**.

(6a*RS*,9a*RS*,9b*SR*)-2,2-Dioxo-5,6,6a,7,8,9,9a,9b-octahydro-4*H*-[1,2,3]oxathiazolo[5,4,3-ij]quinoline ((±)-12)

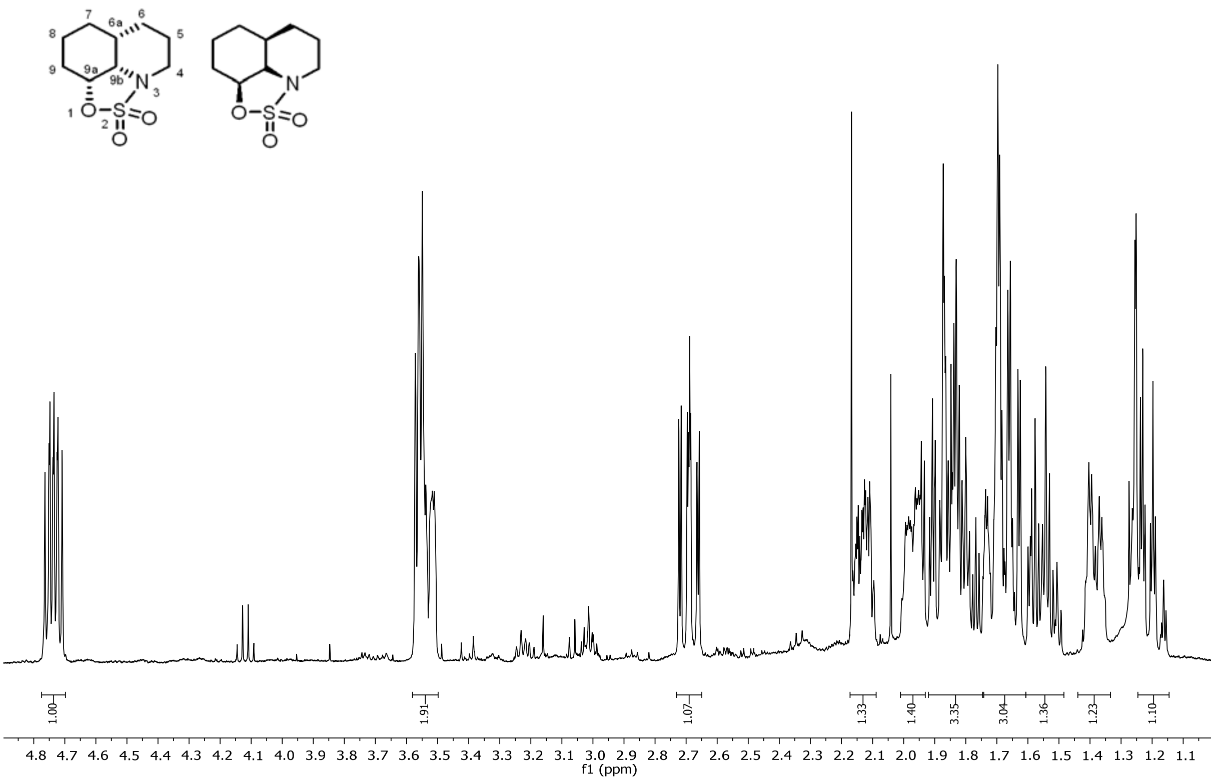

^{1}H NMR spectrum ($CDCl_3$) of (±)-**12**.

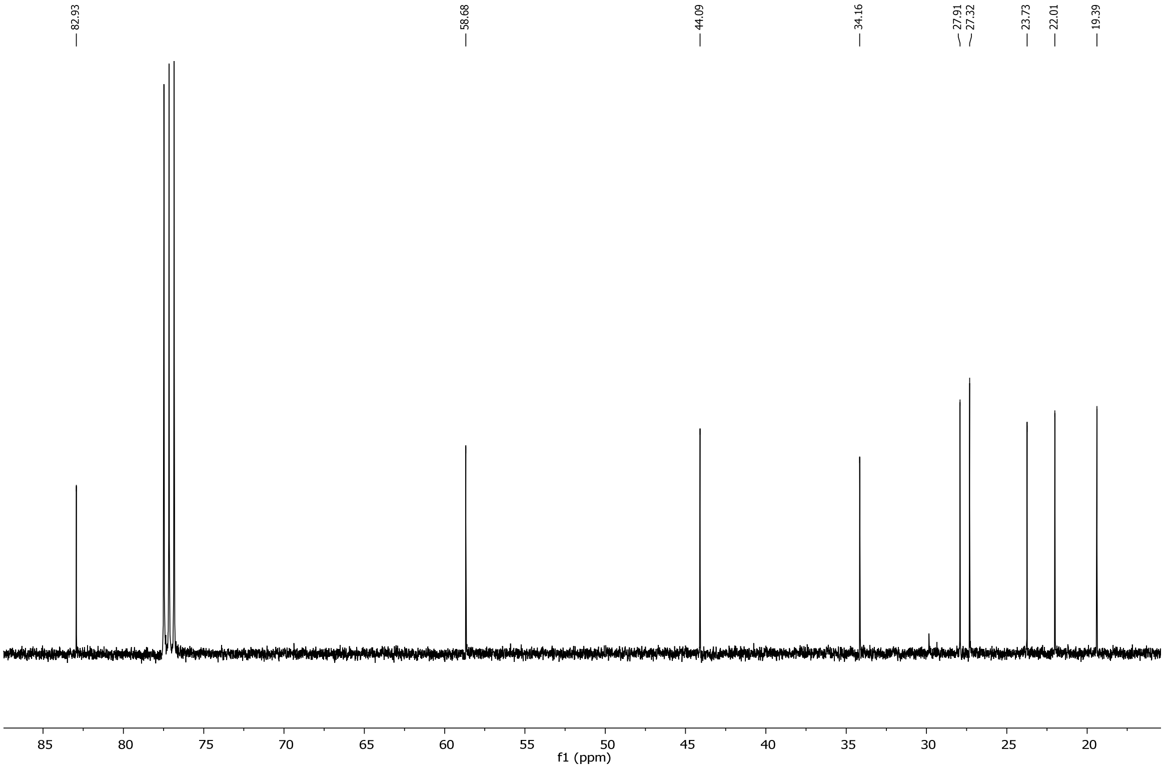

^{13}C NMR spectrum ($CDCl_3$) of (±)-**12**.

(4a*RS*,8*SR*,8a*SR*)-8-(Pyrrolidin-1-yl)-decahydroquinoline ((±)-13)

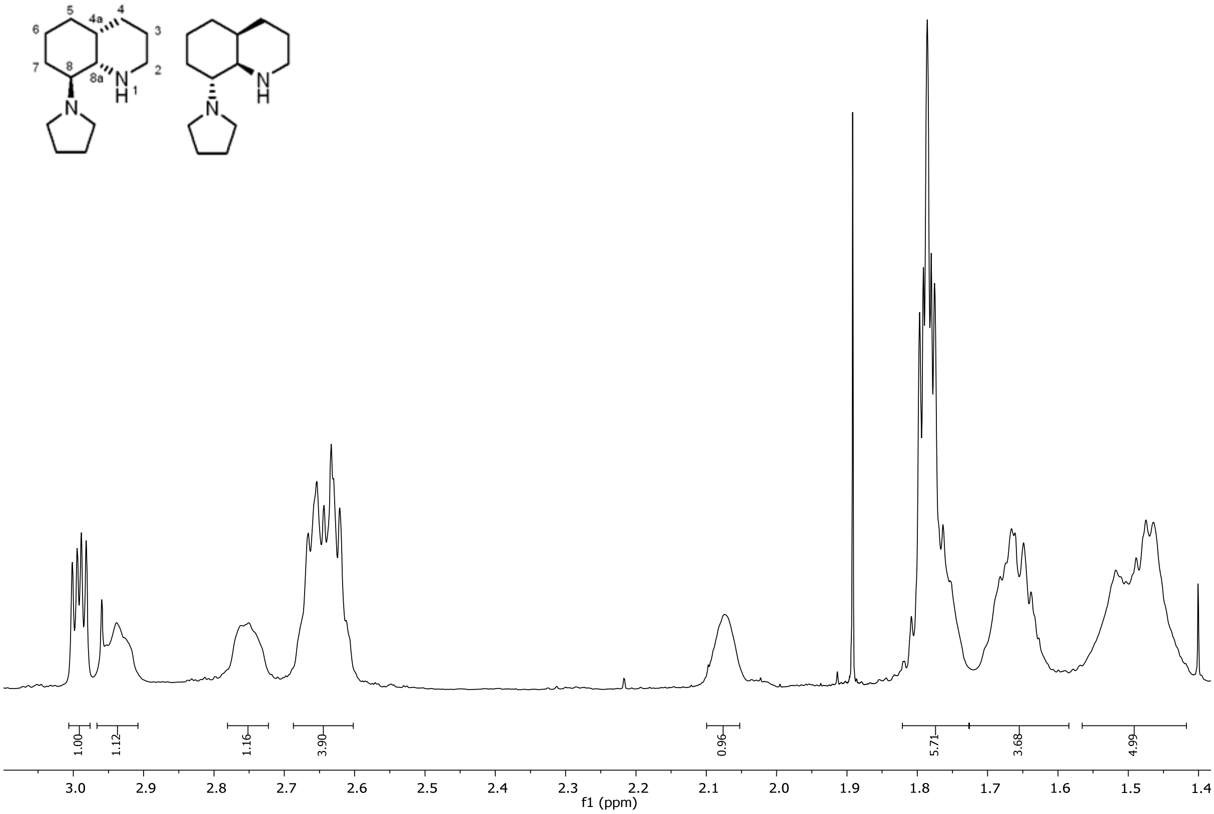

^{1}H NMR spectrum (CD$_3$OD, 26 °C) of (±)-**13**.

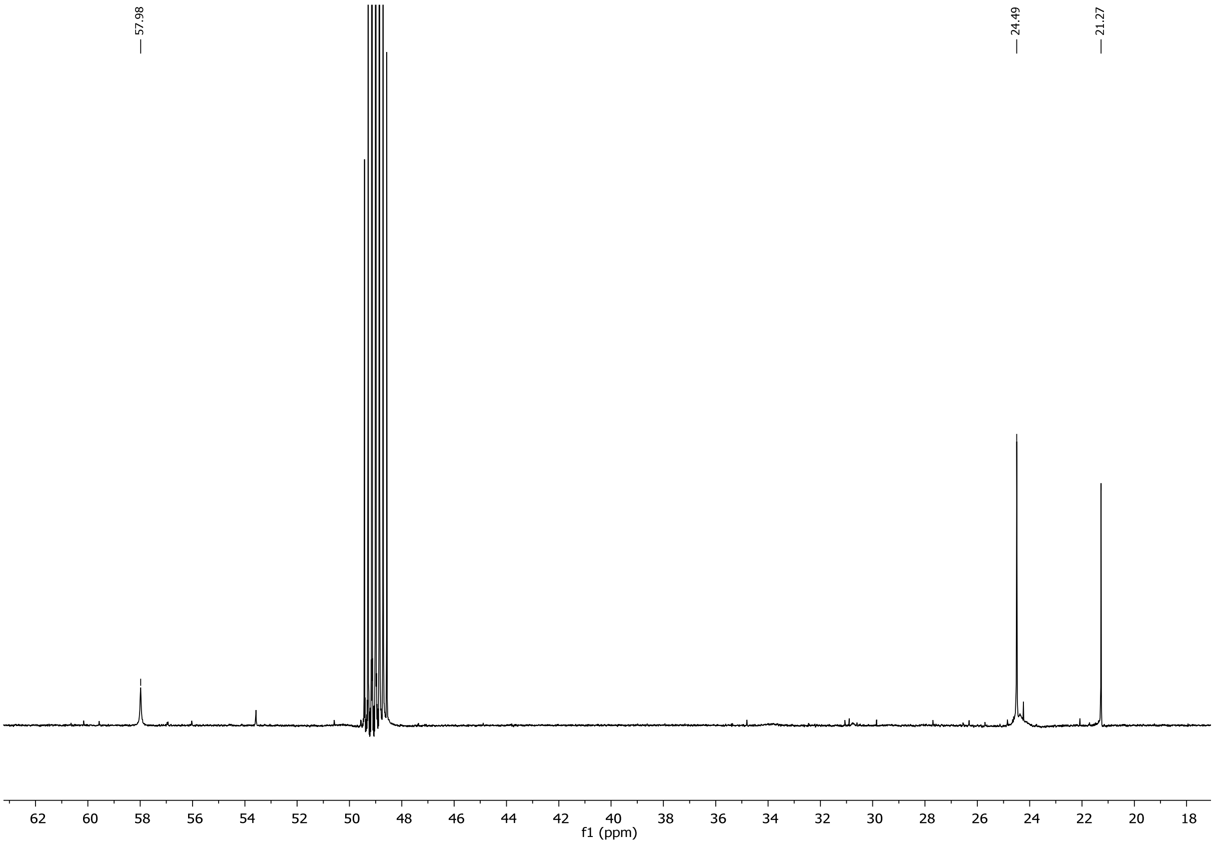

^{13}C NMR spectrum (CD$_3$OD, 26 °C) of (±)-**13**.

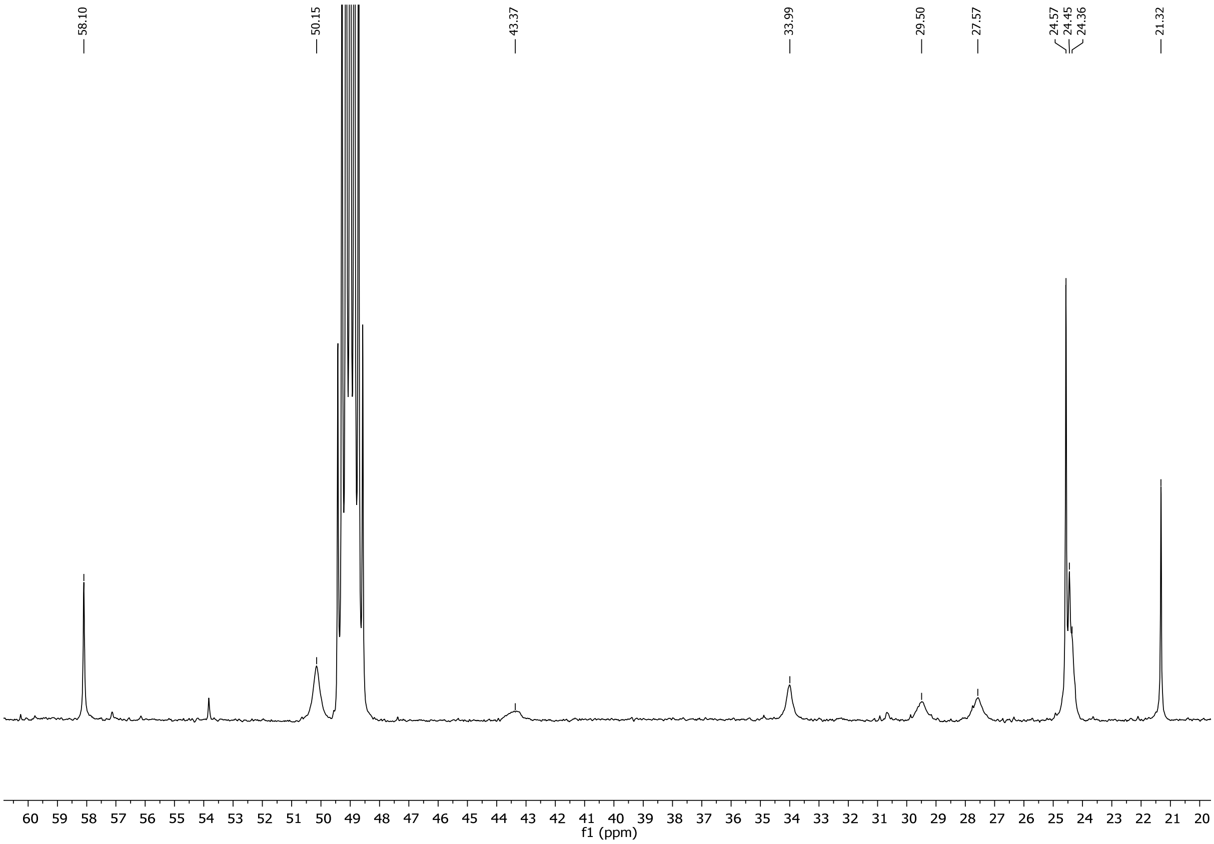

^{13}C NMR spectrum (CD_3OD, 50 °C) of (±)-**13**.

2-(3,4-Dichlorophenyl)-1-[(4a*RS*,8*SR*,8a*SR*)-8-(pyrrolidin-1-yl)-3,4,4a,5,6,7,8,8a-octahydroquinolin-1(2*H*)-yl]ethan-1-one ((±)-4)

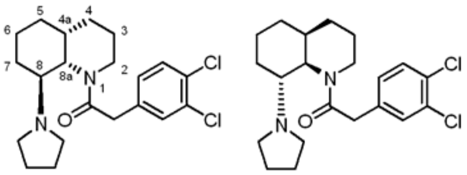

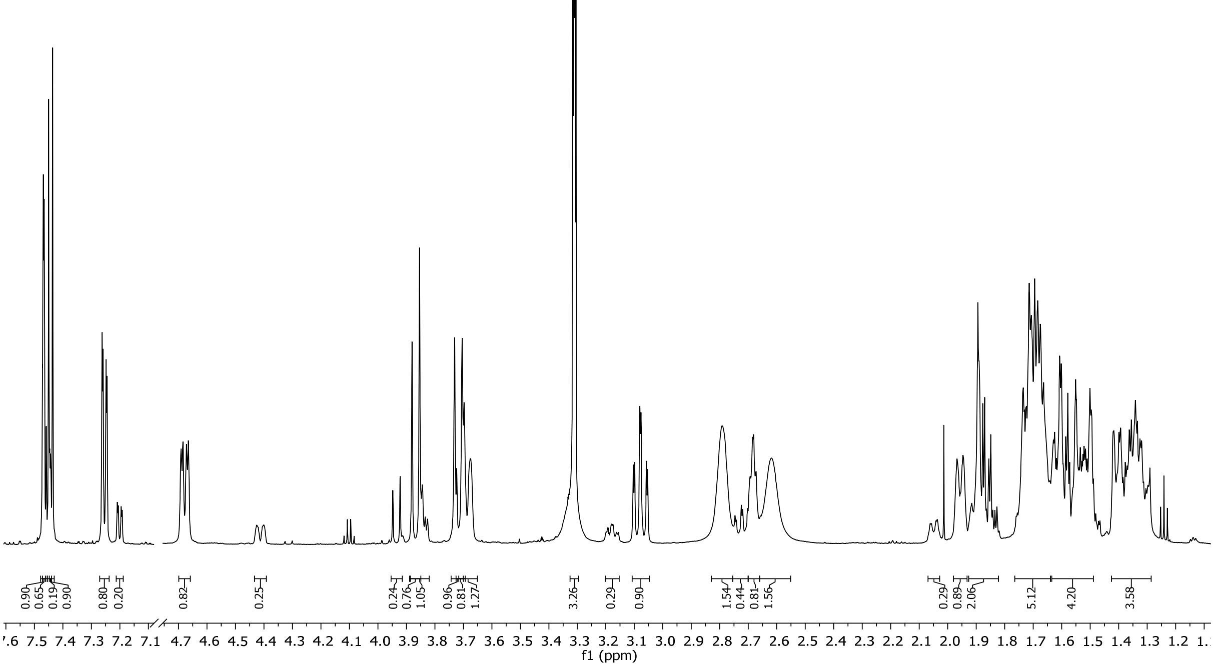

^{1}H NMR spectrum (CD_3OD) of (±)-**4**.

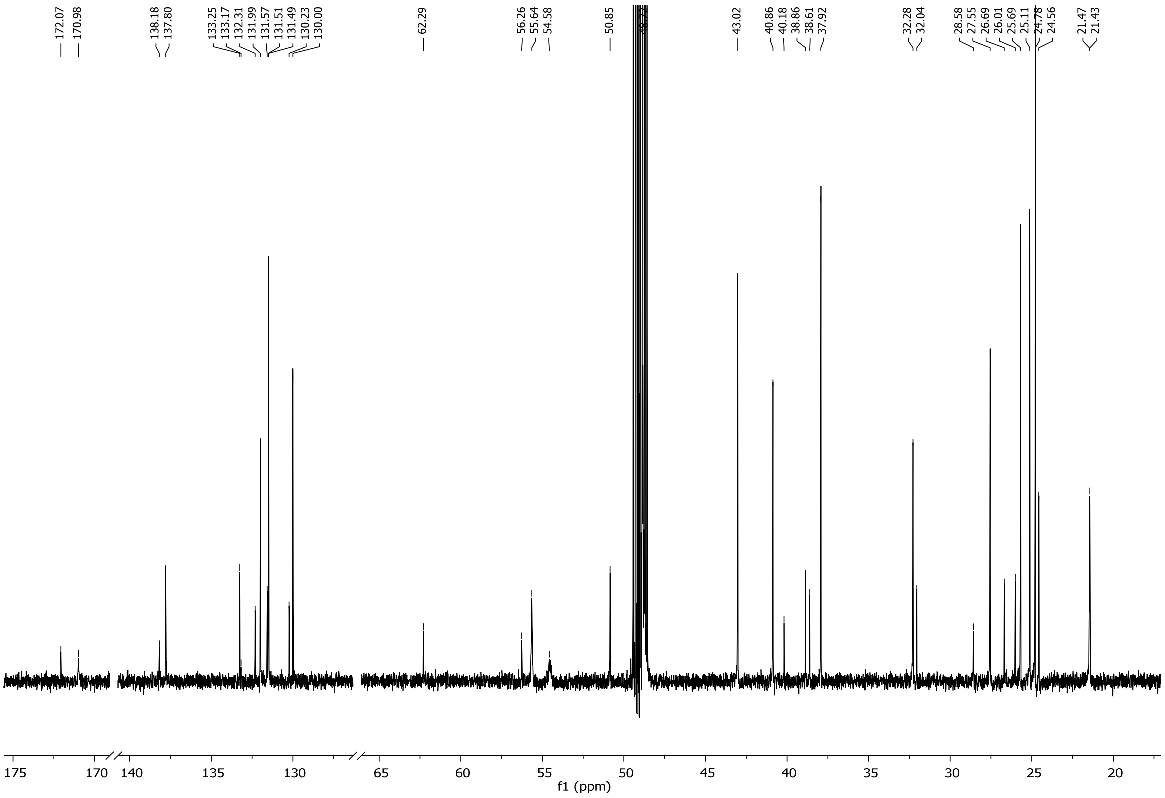

^{13}C NMR spectrum (CD_3OD) of (±)-**4**.

2-(3,4-Dichlorophenyl)-1-[(4a*R*,8*S*,8a*S*)-8-(pyrrolidin-1-yl)-3,4,4a,5,6,7,8,8a-octahydroquinolin-1(2*H*)-yl]ethan-1-one (4)

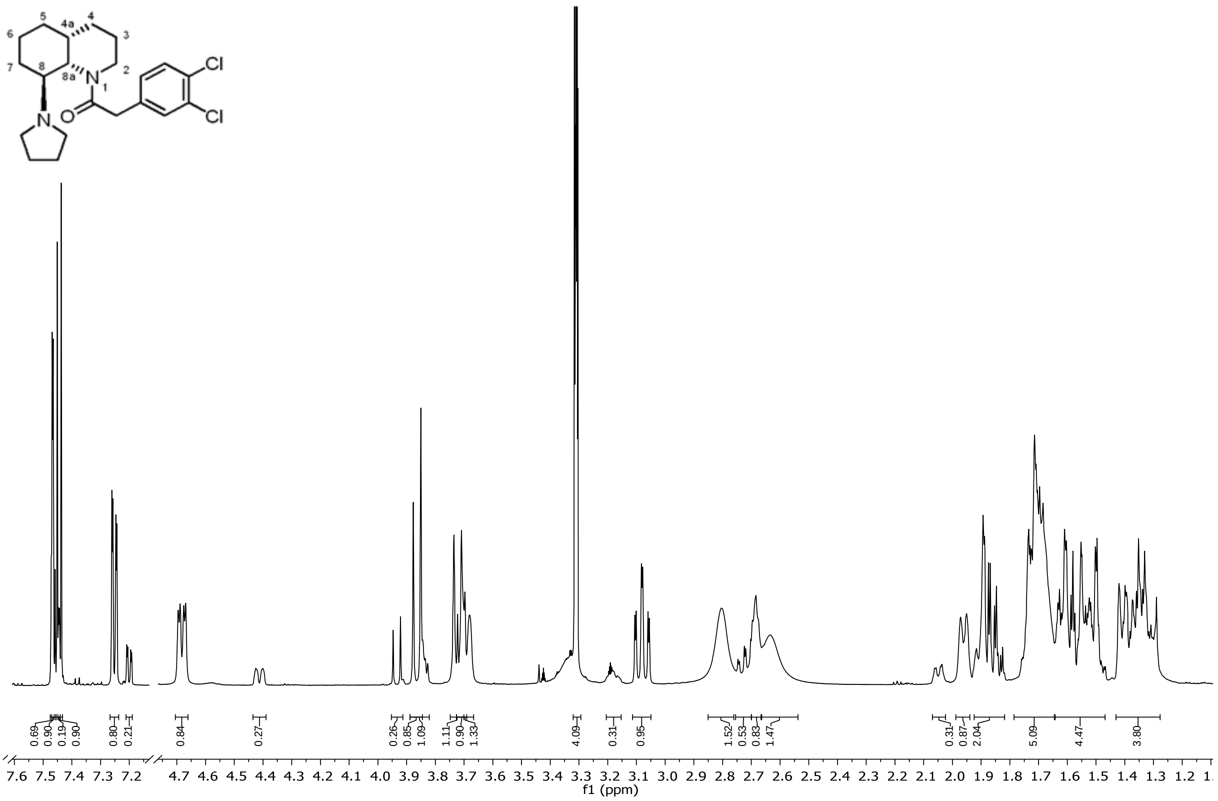

^{1}H NMR spectrum (CD_3OD) of **4**.

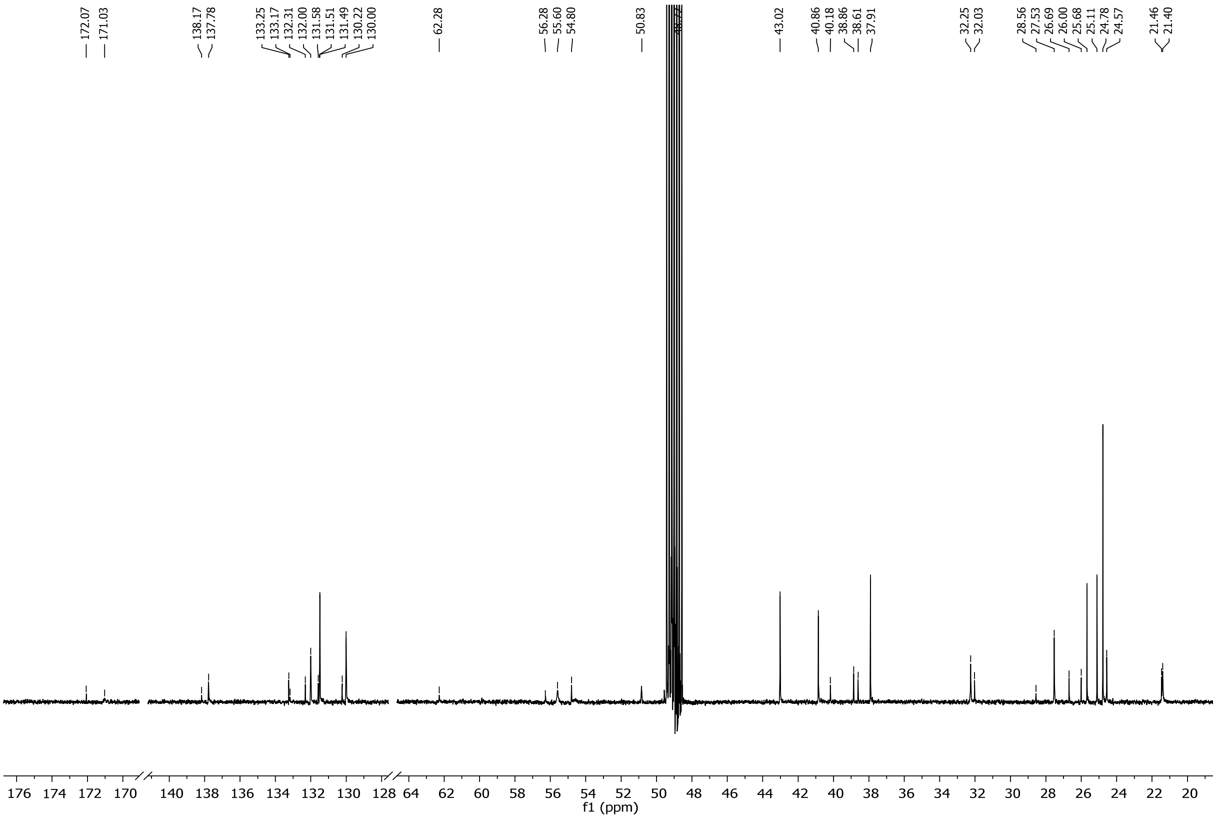

^{13}C NMR spectrum (CD_3OD) of **4**.
